# Circulating Tumor Cells Predict Response to the DLL3-Targeting Bispecific Antibody Tarlatamab

**DOI:** 10.1158/2159-8290.CD-25-1483

**Published:** 2026-01-14

**Authors:** Avanish Mishra, Catherine B. Meador, Kruthika Kikkeri, Quinn Cunneely, Maoxuan Lin, Thomas J. Carmona-LaSalle, Shih-Bo Huang, Remy Bell, Victor Putaturo, Weikun Xia, Joyce H. Liang, Jacy Fang, Sarah San Vicente, Caroline E. Zielinski, Subba R. Digumarthy, Yin P. Hung, Beow Y. Yeap, Jon F. Edd, Michael S. Lawrence, Moshe Sade-Feldman, Debattama R. Sen, Mehmet Toner, Shyamala Maheswaran, Justin F. Gainor, Daniel A. Haber

**Affiliations:** 1 Center for Engineering in Medicine and Surgery, Massachusetts General Hospital and Harvard Medical School, Charlestown, Massachusetts.; 2 Krantz Family Center for Cancer Research, Massachusetts General Hospital, Mass General Brigham Cancer Institute and Harvard Medical School, Charlestown, Massachusetts.; 3 Department of Surgery, Massachusetts General Hospital, Mass General Brigham and Harvard Medical School, Boston, Massachusetts.; 4 Division of Hematology Oncology and Department of Medicine, Massachusetts General Hospital, Mass General Brigham Cancer Institute and Harvard Medical School, Boston, Massachusetts.; 5 Shriners Children’s Hospital, Boston, Massachusetts.; 6 Howard Hughes Medical Institute, Chevy Chase, Maryland.; 7 Department of Radiology, Massachusetts General Hospital, Mass General Brigham and Harvard Medical School, Boston, Massachusetts.; 8 Department of Pathology, Massachusetts General Hospital, Mass General Brigham and Harvard Medical School, Boston, Massachusetts.

## Abstract

The bispecific antibody tarlatamab recruits T cells to cancers expressing the neuroendocrine epitope delta-like ligand 3 (DLL3). Tarlatamab is effective in small cell lung cancer (SCLC), but clinical outcomes vary, and no biomarkers enable patient selection. Single-cell RNA sequencing of SCLC biopsies identifies heterogeneity in *DLL3* expression, and analysis of circulating tumor cells (CTC) distinguishes individual patients as predominantly DLL3^Pos^ or DLL3^Low^. In a prospective cohort of 20 patients, pretreatment DLL3 expression on CTCs predicts tarlatamab clinical benefit (85% sensitivity and 100% specificity). Necrotic CTC clusters in blood accompany treatment-induced tumor lysis. Acquired resistance to tarlatamab is associated in some cases with loss of DLL3 expression but persistence of other targetable neuroendocrine epitopes; in other patients, DLL3 is retained on CTCs but accompanied by systemic markers of T-cell dysfunction. Quantitation of DLL3-positive CTCs identifies patients likely to benefit from tarlatamab, and longitudinal monitoring may guide therapeutic decision-making at the time of acquired resistance.

**Significance:**

CTCs are abundant in SCLC, allowing noninvasive quantitation of epitopes targeted by immune therapies. Patients with at least 25% DLL3-positive CTCs derive clinical benefit from a bispecific antibody targeting this epitope; those with a lower fraction rarely respond. CTC scoring may thus enable stratification of patients for antibody-mediated therapeutics.

## Teaser

A high fraction of circulating tumor cells in small cell lung cancer expressing the neuroendocrine marker DLL3 predicts clinical response to the DLL3-targeted bispecific antibody tarlatamab, providing a liquid biopsy approach to guide cancer immunotherapy.

## Introduction

Small cell lung cancer (SCLC) is an aggressive neuroendocrine malignancy characterized by rapid proliferation and early metastasis, with most patients presenting with advanced disease [extensive stage SCLC (ES-SCLC)] at diagnosis. First-line treatment of ES-SCLC with platinum-doublet chemotherapy combined with an immune checkpoint inhibitor (ICI) typically achieves a robust initial response, but recurrence inevitably occurs, typically within 1 year (1, 2). In contrast to the multiple genetically targeted and immune-based treatments for non–small cell lung cancer (NSCLC), available treatment options for relapsed ES-SCLC are limited and generally associated with poor clinical outcomes, pointing to the unmet need for improved therapeutic options (1–5).

SCLC is genetically homogeneous, with near-universal loss-of-function mutations in tumor suppressors *TP53* and *RB1* but without recurrent, targetable activating mutations (3, 4). Surgical resections are rare in SCLC, as are serial tumor biopsies, with most cases treated empirically, based on a single initial diagnostic needle biopsy. Recently, therapeutic antibodies targeting cell surface epitopes have emerged as an important therapeutic strategy in SCLC, supported by the unique tumor-associated neuroendocrine markers, which are not shared by normal adult tissues. Among these, delta-like ligand 3 (DLL3) encodes an inhibitory Notch pathway ligand reported to be highly expressed on the cell surface of neuroendocrine tumors, including more than 80% of SCLC, and absent in normal adult tissues (5–7). The DLL3 bispecific T-cell engager (BiTE) tarlatamab was granted accelerated FDA approval for the treatment of relapsed SCLC, and it is now considered a new standard of care for treatment-refractory advanced SCLC. This approval was based on data from the registrational phase II DeLLphi-301 trial of tarlatamab in which objective responses were observed in 32% to 40% of patients with previously treated SCLC (median two prior lines of therapy), with more than half of the responding patients remaining on therapy for more than 12 months (8, 9). More recently, in the phase III DeLLphi-304 trial, tarlatamab was shown to produce significant improvement in overall survival and progression-free survival, compared with chemotherapy, among patients with ES-SCLC who had been previously treated with chemotherapy (10).

Despite the clinical success of tarlatamab, more than 50% of patients with SCLC will progress within 6 months of initiating therapy (10). In addition, the risk of cytokine release syndrome (CRS) mandates hospital admission for all patients receiving their first two doses, which together with the unpredictability of response, limits the general availability of tarlatamab for patients with SCLC. To date, efforts to identify predictive biomarkers for tarlatamab have largely centered on immunohistochemistry (IHC) staining, but testing of archival biopsy specimens has found near-universal expression of DLL3 in this tumor type (11). For example, in the initial phase I study of tarlatamab, Paz Ares and colleagues reported DLL3 staining (>1%) in 95% of tumor specimens, and studies to date have reported objective responses among patients with both DLL3-positive and DLL3-negative tumors (6, 8). Thus, the current FDA label for tarlatamab does not include a biomarker selection strategy.

Four molecularly defined subtypes of SCLC have been identified, with different levels of neuroendocrine differentiation based on expression signatures linked to key transcriptional regulators. However, intratumoral heterogeneity in these signatures, along with switching from one subtype to another, has also been reported (12, 13). Altered expression of DLL3 as a function of prior chemotherapy has also been observed in some cases (14), pointing to the potential need for timed pretreatment evaluation to guide administration of tarlatamab and other DLL3-targeted therapeutics.

Although serial tumor biopsies are uncommonly obtained in SCLC, the abundant shedding of circulating tumor cells (CTC) into the bloodstream presents an opportunity for the measurement of tumor burden through noninvasive liquid biopsy (15–22). *Ex vivo* cultures of CTCs from patients with SCLC have enabled co-clinical trial experiments in mice, testing response to established and novel drug regimens (23, 24). As intact cancer cells in circulation, CTCs present the ideal liquid biopsy for quantitation of cell surface marker expression, a measurement that is not feasible using ctDNA-based tests. However, quantitative testing of CTCs for expression of relevant cell surface markers requires their isolation without any selection bias (22). Neuroendocrine cancers generally express low levels of epithelial cell adhesion molecule (EpCAM), the epithelial cell surface marker most commonly used to enrich CTCs (25), with some neuroendocrine CTC populations completely lacking in EpCAM expression (26). Furthermore, another commonly used CTC enrichment technology, based on their presumed larger cell size compared with normal leukocytes (27), is challenged by the characteristic small size of SCLC cells, which approximates that of lymphocytes in the blood (26).

We have previously described a CTC enrichment platform (CTC-iChip) that applies highly efficient microfluidic cell sorting technology to remove normal hematopoietic cells from blood samples, using inertial microfluidic depletion of red blood cells (RBC), combined with the removal of white blood cells (WBC) through magnetically conjugated antibodies against widely shared leukocyte antigens. This “negative depletion technology” isolates untagged CTCs without bias for either cell surface markers or cell size (28–31). Here, we established a staining approach for single-cell measurements of DLL3 protein expression on CTCs, enabling noninvasive monitoring of patients prior to initiation of tarlatamab treatment and longitudinally thereafter. Contrary to IHC-based diagnostic testing of archival biopsy specimens, we find that DLL3 protein expression on CTCs is highly variable across different patients with advanced SCLC, with individual patients having predominantly DLL3-positive or DLL3-low CTCs, a pattern that is highly predictive of tarlatamab response. Acquired resistance to tarlatamab in patients who present with predominantly DLL3-positive CTCs is associated either with loss of DLL3 expression on CTCs or with persistence of epitope expression but coincident evidence of systemic T-cell dysfunction. Importantly, the reduction in DLL3 expression associated with acquired tarlatamab resistance is not accompanied by loss of other neuroendocrine or SCLC-enriched epitopes that remain targetable in such cases.

## Results

### Clinical Cohort: Patients with ES-SCLC Treated with Tarlatamab

To evaluate the relationship between clinical outcomes and DLL3 expression on CTCs, we collected baseline and longitudinal blood specimens for CTC analysis from 32 patients with ES-SCLC treated at our institution (cohort A). Pretreatment blood sampling was performed on the day of initiation of tarlatamab treatment and subsequently at predefined on-treatment intervals [cycle (C) 1 day (D) 2, C1D8, and C1D9], at the time of restaging scans, and at disease progression. Baseline clinical and pathologic features of this cohort are shown in Supplementary Table S1, and representative IHC images of the cohort are presented in Supplementary Fig. S1. All patients had previously received platinum-doublet chemotherapy, and the majority (96%) had received prior ICI. The median number of prior lines of therapy was 2 (range 1–5). At the time of data cutoff (October 27, 2025), the median time on treatment was 145.5 days (range: 7–291). To more closely recapitulate the DeLLphi-301 population, we defined a response-evaluable population using prespecified inclusion criteria as follows: (i) pathologically confirmed *de novo* SCLC (i.e., excluding histologic transformation from NSCLC to SCLC), (ii) absence of untreated/active central nervous system (CNS) metastases (due to lack of adequate data for CNS activity of tarlatamab), (iii) measurable lesions as defined by Response Evaluation Criteria in Solid Tumors (RECIST) version 1.1 (32), and (iv) adequate follow-up for response assessment. In total, 12 patients were excluded from the response-evaluable population due to inadequate follow-up imaging for response assessment (*n* = 2), inadequate baseline sample collection and CTC assessments (*n* = 3), active brain metastases (*n* = 2), and nonclassical SCLC cases (including extrapulmonary neuroendocrine carcinomas and transformed SCLC; *n* = 2 and *n* = 3, respectively) as shown in Supplementary Table S2. The remaining 20 patients met the inclusion criteria for the evaluation of CTC DLL3 expression and its correlation with the clinical response evaluation.

### Scoring of CTCs for DLL3 Expression Using Multispectral Imaging

**Figure 1.**
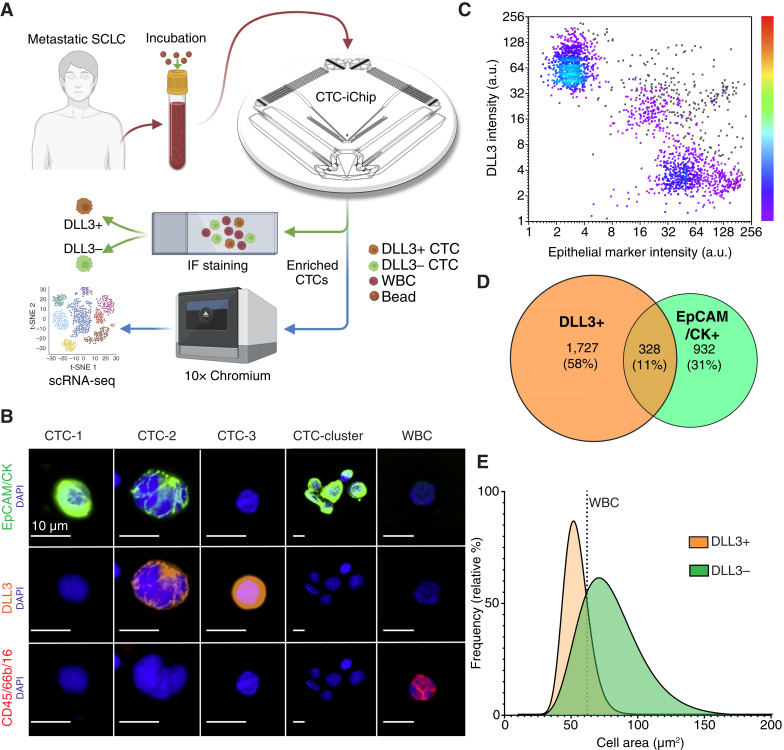
CTC enrichment and phenotypic classification in SCLC blood samples. **A,** Schematic representation of CTC-iChip microfluidic processing of blood samples from patients with SCLC receiving tarlatamab (cohort A). Whole blood (10–20 mL) is incubated with antibodies against WBCs conjugated with magnetic beads and passed through the CTC-iChip, removing RBCs, platelets, and unbound beads through initial size-based separation, followed by magnetic sorting of remaining nucleated cells to remove WBCs. The CTC-enriched cell product is stained using a multiplexed IF panel and analyzed using image-based CTC classification. In selected cases, samples with highly abundant CTCs may be subjected to 10× scRNA-seq. **B,** Representative fluorescence microscopy of CTCs stained with 4′,6-diamidino-2-phenylindole (DAPI) (nuclear; blue); a cocktail of antibodies against EpCAM, pan-CK, and CK19 (epithelial markers; green); antibody against DLL3 (neuroendocrine marker; orange); and a cocktail of antibodies against hematopoietic cells (CD45, CD66b, and CD16; red). Three single CTCs with different expression patterns are shown, along with a CTC cluster and a representative WBC. **C,** FACS plot of single CTCs isolated through tumor epitope-independent depletion of hematopoietic cells (total 2,987 CTCs from 20 untreated patients) showing the relative intensity of staining for the targeted neuroendocrine marker DLL3 versus epithelial markers (EpCAM, pan-CK, and CK19). **D,** Quantification of single-cell expression from **C**, showing the number of CTCs expressing DLL3, epithelial markers, or both. Only a small subset of CTCs expresses both markers, highlighting heterogeneity in surface marker expression within the CTC population. **E,** Size distribution of CTCs expressing only DLL3 (*N* = 1,134) versus those expressing only epithelial markers (*N* = 503), with dotted line indicating the average size of WBCs (61 µm^2^). Cell area is calculated and plotted as a percentage of all cells expressing either DLL3 or epithelial markers. t-SNE, t-distributed stochastic neighbor embedding.

For each time point, we processed 10 to 20 mL of whole blood through the CTC-iChip microfluidic leukocyte-depletion platform, enriching for SCLC cells in circulation, independent of either cell size or tumor cell–specific epitopes (Fig. 1A; Supplementary Fig. S2A; ref. 33). Blood specimens were lightly fixed at the time of collection (Streck) and processed within 72 hours of collection. Following a mean 10^4^ enrichment, cells were stained for the targeted epitope DLL3; a combination of EpCAM, pan-cytokeratin (pan-CK), and CK19 as epithelial markers; and a combination of hematopoietic markers CD45, CD66b, and CD16 to exclude contaminating leukocytes. The narrow wavelength and quantifiable scoring provided by multispectral imaging make it possible to assess the relative expression of the three fluors without signal overlap. NCI-H82, DMS79, and A549 cancer cell lines, along with WBCs, were used as positive and negative controls to calibrate the DLL3 signal measured on CTCs (Fig. 1B and C; Supplementary Fig. S2B and S2C). CTCs were defined as CD45/66b/16-negative cells that were positive for EpCAM/pan-CK/CK19 and DLL3, EpCAM/pan-CK/CK19 alone, or DLL3 alone (Fig. 1B; Supplementary Fig. S3A–S3C). A median of 0.2 and a mean of 0.18 marker positive cells/mL (range 0–3/10 mL) were evident in control healthy donors without cancer (*N* = 5).

Among the 20 evaluable patients, the median number of CTCs at pretreatment baseline was 2 CTCs/mL of blood (40 CTCs/20 mL collected; range 2 to 1,416 CTCs/20 mL), with microfluidic sorting achieving a mean purity of 0.19% CTCs among contaminating leukocytes (Supplementary Table S3). Remarkably, DLL3 protein expression on individual CTCs is either strongly positive or negative, with few intermediate levels of expression (Fig. 1C). Some DLL3-positive CTCs also stain for epithelial proteins (EpCAM/pan-CK/CK19), whereas others are negative for epithelial markers and are only identified by virtue of DLL3 staining. Among all 2,987 CTCs from the entire cohort that were scored at the pre-tarlatamab baseline time point, 1,727 (58%) were positive for only DLL3, 932 (31%) were positive for only epithelial proteins, and 328 (11%) were positive for both DLL3 and epithelial markers (Fig. 1D). The mean size of SCLC CTCs is comparable with that of lymphocytes (10.5 µm diameter; 77.7 μm^2^ cell area), with DLL3-positive CTCs being even smaller than DLL3-negative CTCs (Fig. 1E; Supplementary Fig. S4A–S4C). Thus, using a CTC enrichment technology that is unbiased for the expression of epithelial markers or for cell size to identify SCLC cancer cells in the blood circulation, we find that the majority of CTCs staining for the tarlatamab target DLL3 lack detectable expression of epithelial proteins. Although small cancer cells are characteristic of SCLC, these CTCs, identified solely by their expression of a neuroendocrine marker, are even smaller in cell diameter than SCLC cancer cells that express epithelial markers without DLL3 (Fig. 1E).

### Single-Cell RNA Sequencing Analysis of SCLC Tumors

**Figure 2.**
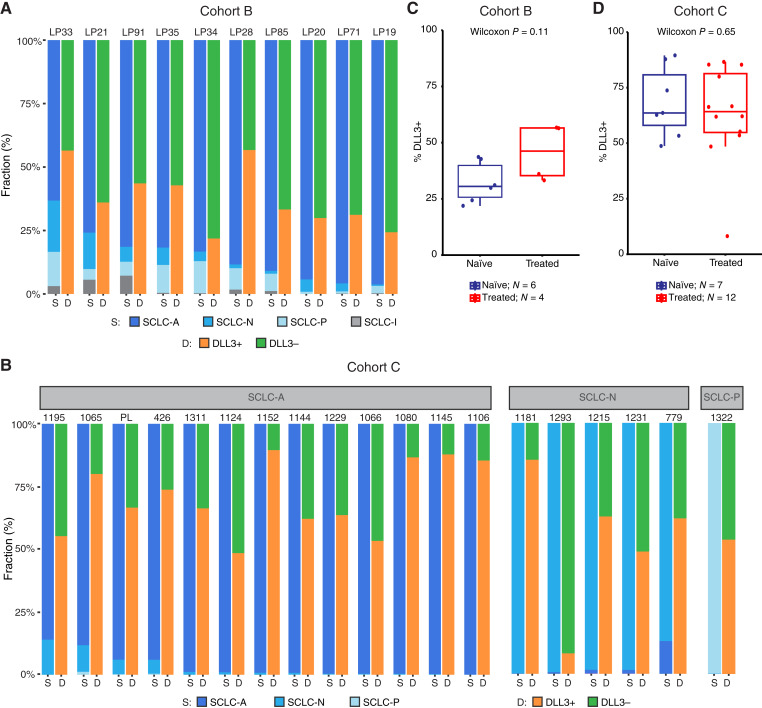
scRNA expression of *DLL3* across SCLC tumors with different molecular subtypes. **A,** Bar graphs showing the fraction of single cells within a SCLC tumor expressing RNA signatures of the four different molecular subtypes, SCLC-A, SCLC-N, SCLC-P, and SCLC-I (blue shades, left bar), paired with the matching fraction of SCLC cells expressing *DLL3* (orange) or no *DLL3* (green). In this cohort, all tumors are classified as predominantly SCLC-A but have subpopulations of single cells with other molecular signatures. For each tumor (*N* = 10, cohort B), cancer cells are first identified by RNA-inferred CNV, followed by determination of molecular subtype classification and *DLL3* expression. **B,** Molecular subtype classification and *DLL3* expression across single cells from a publicly available SCLC tumor dataset [*N* = 19, cohort C; ref. (34)]. In this cohort, tumors are classified as predominantly SCLC-A, SCLC-N, or SCLC-P, with more limited single-cell heterogeneity across molecular signatures. For CNV-confirmed single cancer cells within each tumor, the matched bar shows *DLL3*-positive (orange) and *DLL3*-negative (green) cells. **C,** Fraction of *DLL3*-positive single cells within treatment-naïve versus chemotherapy-treated SCLC tumors in cohort B (Wilcoxon *P* = 0.11). **D,** Fraction of *DLL3*-positive cells within untreated and chemotherapy-treated groups in SCLC tumors from cohort C (Wilcoxon *P* = 0.65). D, DLL3; S, subtype; LP, Lung Patient; PL, Plueral Effusion.

The fraction of CTCs that stain negative for DLL3 is unexpected, given the reported near-universal expression of this neuroendocrine marker in SCLC, leading us to analyze primary tumors at the single-cell level using RNA sequencing (RNA-seq). We examined single SCLC tumor cells within biopsies obtained from 10 patients with ES-SCLC treated at our institution (cohort B), as well as a published database of 19 SCLC tumors (cohort C; ref. 34). RNA-based molecular studies have recently annotated SCLC into four classes, based on expression patterns linked to key transcriptional regulators: two neuroendocrine subtypes are correlated with transcriptional signatures of either ASCL1 (SCLC-A; approximately 70% of cases) or NeuroD1 (SCLC-N; 18%), and two more rare subtypes are associated with either a POU2F3 transcriptional signature (SCLC-P; 10%) or a more general inflammatory signature (SCLC-I; 2%; refs. 12, 13). To better understand the pattern of *DLL3* expression across these subtypes, we first performed 10× single-cell RNA-seq (scRNA-seq) on biopsy specimens from cohort B (six treatment-naïve; four previously treated; Supplementary Data S1). We used cancer-associated aneuploidy to ascertain the identity of individual tumor cells within the biopsy specimens through RNA-based inferred copy-number variation (CNV; Supplementary Figs. S5A–S5D, S6, and S7). In comparison with standard clinical histologic identification of cancer cells within a mixed tissue section that includes both cancer and stromal cells, we note that the use of molecular CNV-driven scoring of individual cancer cells may provide a different denominator for the total number of tumor cells that are used to calculate the fraction of cells positive for DLL3. Also, to ensure against technical “dropout” of reads from rare transcripts in scRNA-seq analyses, we applied correctional algorithms based on the detection of multiple Notch pathway transcripts that are coregulated with DLL3 (Supplementary Fig. S8A and S8B; Supplementary References). All specimens in cohort B were defined as predominantly neuroendocrine SCLC-A subtype, with scRNA-seq analysis showing 63% to 96% of cells with the SCLC-A expression signature. However, each tumor also contained smaller fractions of cells representing SCLC-N, SCLC-P, and/or SCLC-I signatures (Fig. 2A), consistent with recent reports describing molecular intratumoral heterogeneity in SCLC (12, 35–38). All tumors in cohort B show heterogeneity for *DLL3* expression, with *DLL3*-positive cancer cells comprising a median of 35% (range 22%–57%; expression defined by the presence of at least one unique molecular identifier (UMI) for *DLL3* in a cell; Fig. 2A).

To confirm and extend these results, we reanalyzed a published dataset of 19 SCLC cases (34), including different predominant molecular signatures SCLC-A (*n* = 13), SCLC-N (*n* = 5), and SCLC-P (*n* = 1; cohort C; Fig. 2B). A median of 64% (range: 8%–90%) of all single cancer cells from these biopsies express *DLL3* mRNA. Interestingly, in both cohorts B and C, the fraction of individual cancer cells expressing *DLL3* transcripts is not significantly correlated with the relative proportion of cells with various molecular subtype signatures (Fig. 2; Supplementary Figs. S9A and S9B and S10; and Supplementary References). However, pseudobulk expression of *DLL3* mRNA—aggregated gene expression profile of all cancer cells for each tumor—is elevated in SCLC-A tumors compared with either SCLC-N or SCLC-P tumor (Supplementary Fig. S11A and S11B). Thus, scRNA-seq analysis confirms the quantitatively increased levels of *DLL3* expression associated with the ASCL1-driven SCLC-A molecular subtype, but it also shows that SCLC tumors harbor a mixture of DLL3-positive and DLL3-negative cells independent of the molecular subtypes, suggesting a shared lineage-dependent feature.

Diagnostic biopsies for SCLC are obtained prior to treatment initiation and are rarely repeated over the course of treatment, which is largely empirical. It is therefore unclear whether multiple cycles of chemotherapy with or without ICI therapy generate selective pressure affecting the tumor expression of *DLL3*. Together, SCLC cohorts B and C include 13 treatment-naïve cases and 16 cases treated with chemotherapy and ICI. At the single-cell level, our analysis does not indicate a difference in the fraction of *DLL3*-positive cancer cells between treatment-naïve and treated cases (cohort B: median 31% DLL3-positive in treatment-naïve cases vs. 46% in treated cases, *P* = 0.11; cohort C: 63.6% in treatment-naïve vs. 64.2% in treated cases, *P* = 0.65; Fig. 2C and D). Altogether, these findings confirm the heterogeneity in *DLL3* expression within SCLC at the single-cell level, and they indicate that neither transcriptional signature–defined molecular subtypes nor prior chemotherapy and immune checkpoint treatment have a major effect on the fraction of individual cancer cells expressing *DLL3* within SCLC tumors.

### Pretreatment DLL3 Expression on CTCs Predicts Response to Tarlatamab Therapy

**Figure 3.**
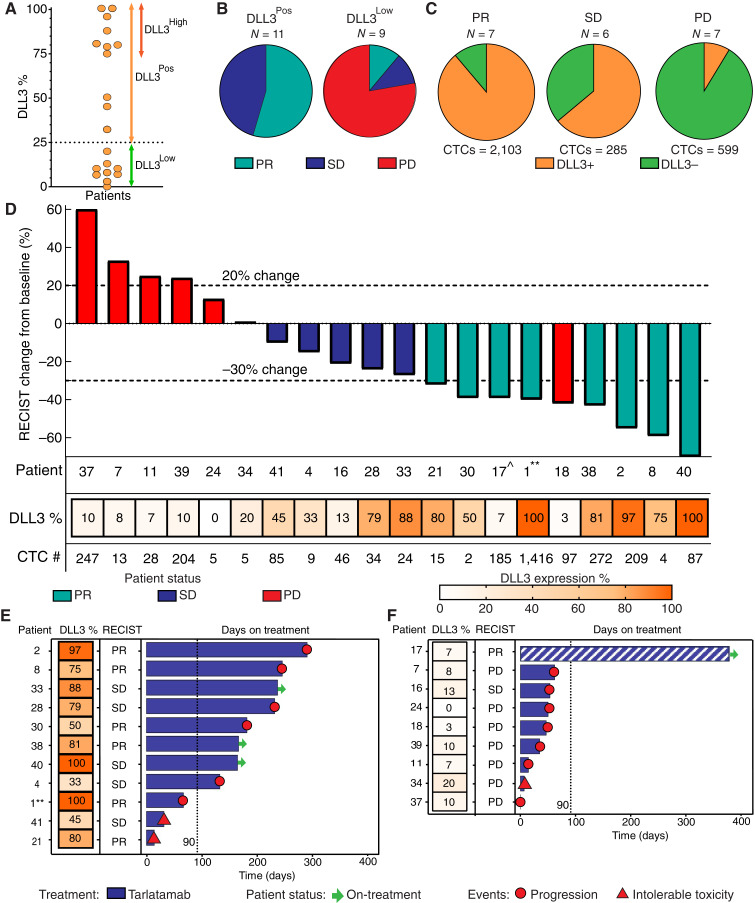
Correlation between pretreatment CTC expression of DLL3 and clinical response to tarlatamab. **A,** Percentage of CTC staining for DLL3 by IF imaging across individual patients at pretreatment baseline (cohort A, *N* = 20). A 25% cutoff was used to stratify patients into DLL3^Pos^ and DLL3^Low^ groups. **B,** Venn diagram showing the fraction of DLL3^Pos^ (*N* = 11) and DLL3^Low^ (*N* = 9) patients with either PR (teal), SD (blue), or PD (red) following tarlatamab therapy. Patients are categorized by their best RECIST-defined systemic response. **C,** Venn diagram showing the distribution of DLL3-positive (orange) and DLL3-negative (green) CTCs among patients stratified by their clinical response as either PR (*N* = 7), SD (*N* = 6), or PD (*N* = 7). **D,** Waterfall plot showing best systemic response (RECIST) for each patient, along with the percentage of DLL3-positive CTCs and the total number of CTCs at pretreatment baseline. Direction of bar represents treatment-induced change in measured lesion, and bars are color-coded based on overall patient response (PR, SD, and PD, *N* = 20). **E** and **F,** Swimmer’s plot showing longitudinal treatment timelines for DLL3^Pos^ patients (*N* = 11; **E**) and DLL3^Low^ patients (*N* = 9; **F**). Bars represent time on tarlatamab treatment for each patient, with key clinical milestones (progression, intolerable toxicity, or ongoing treatment) annotated. Patient 17 is indicated in dashed blue and white lines to highlight the aberrant DLL3 splicing isoform. Color-coded fraction of DLL3-positive CTCs for each patient is shown on the left, as is the RECIST classification. ^**^CNS-only progression with systemic PR; ^aberrant DLL3 splicing isoform.

Among the 20 patients in cohort A who met the criteria for response evaluation outlined above, 7 (35%) had a systemic partial response (PR), 6 (30%) had stable disease (SD), and 7 (35%) had progressive disease (PD) as their best overall response (Fig. 3). Of note, one patient (patient 1) had a systemic PR based on a 40% decrease in the sum of target lesions but also had new CNS-only progression on first response assessment. Our observation of systemic objective responses (PR) in 35% of patients and of clinical benefit (PR and SD) in 65% of patients is consistent with the antitumor activity of tarlatamab in the DeLLphi-301 study (8).

Compared with the relatively uniform fraction of *DLL3-*expressing tumor cells across biopsies from different patients (cohorts B and C), analysis of CTCs from the 20 patients in cohort A shows a clearly divergent pattern, with some patients having a low fraction of DLL3-positive cells and others having very high fractions. Applying a previously annotated cutoff of 25% DLL3-positive cancer cell fraction (6, 39), 9 (45%) patients in cohort A have fewer than 25% DLL3-positive CTCs (DLL3^Low^; median: 8%, range: 0–20), whereas 11 (55%) patients have more than 25% DLL3-positive CTCs (DLL3^Pos^; median: 76%, range: 33–100). Of note, 8 of the 11 DLL3^Pos^ patients have very high DLL3-positive CTC fractions (DLL3^High^; Fig. 3A).

Matched tumor biopsies for the CTCs from patients in cohort A were not available for scRNA-seq analysis, but the observed interpatient heterogeneity in DLL3 expression on CTCs differs from the relatively uniform single-cell expression fraction evident in tumor biopsies from cohorts B and C. This difference may reflect biological properties as well as technical considerations. CTCs are likely enriched for invasive cancer cell populations that drive tumor progression, whereas tumor biopsies include a more complex tumor microenvironment. Furthermore, immunofluorescence (IF)-based imaging of single cells in the circulation enables more quantifiable scoring compared with IHC staining of tumor biopsy specimens. Indeed, in previous studies, tissue-based DLL3 IHC scoring proved to be an unreliable marker in predicting response to tarlatamab, with responses observed both among patients with DLL3-positive and -negative tumor samples (8). We therefore tested CTC-based scoring for pretreatment patient stratification and then longitudinally measured its expression in CTCs through the acquisition of drug resistance.

We applied the 25% DLL3-positive CTC cutoff to evaluate tarlatamab responses across the 20 patients in cohort A. Collectively, among 11 patients who were scored as DLL3^Pos^, all (100%) experienced clinical benefit with tarlatamab treatment, including 6 (55%) patients with PR and 5 (45%) with SD (Fig. 3B). In contrast, of the nine patients scored as DLL3^Low^, 7 (78%) patients had PD, 1 (11%) patient had SD, and 1 (11%) patient had PR (Fig. 3B). Notably, among the 11 DLL3^Pos^ cases, the subset of 8 DLL3^High^ patients (>75% DLL3-positive CTCs) included 6 (75%) PR and 2 (25%) SD, whereas all 3 (100%) patients with intermediate DLL3 positivity (25%–75%) had SD. Taken together, scoring DLL3^Pos^ versus DLL3^Low^ patients for predicting clinical benefit (PR and SD) from tarlatamab has a sensitivity of 85% [95% confidence interval (CI): 55%–98%], a specificity of 100% (one-sided 97.5% CI: 59%–100%), and a positive predictive value of 100% (one-sided 97.5% CI: 72%–100%).

We also evaluated DLL3 expression on CTCs stratified by clinical response. The 7 patients with PR had a total of 2,103 CTCs, of which 1,868 (89%) were DLL3-positive; the 6 patients with SD had a total of 285 CTCs, including 182 (64%) DLL3-positive; and the 7 patients with PD had a total of 599 CTCs, of which only 52 (9%) were DLL3-positive (Fig. 3C). Individual clinical responses are illustrated as a waterfall plot, along with time on treatment plots (Fig. 3D–F). Whereas all 11 DLL3^Pos^ patients had either PR or SD, 2 of 9 DLL3^Low^ patients (patients 17 and 18) also had tumor shrinkage. These cases are noteworthy: Patient 18 is classified as PD because of on-treatment development of new metastases despite shrinkage of the target measurable lesion. This may reflect heterogeneity across metastatic lesions. Patient 17 is of particular interest, given a sustained PR despite abundant DLL3-negative CTCs. Remarkably, PCR and sequencing of the *DLL3* transcript from this patient’s CTCs reveal the complete absence of a normal splice variant within the cytoplasmic tail (Supplementary Figs. S12A–S12C). Although this patient-specific abnormality does not alter the therapeutic effect of tarlatamab, which targets the extracellular domain of DLL3, the splicing alteration flanks the cytoplasmic recognition site for the DLL3 diagnostic antibody used in this study, and it seems to preclude DLL3 scoring in our assay. Although this case may be an outlier, it suggests that optimal DLL3 scoring should employ a matched diagnostic and therapeutic antibody. Taken together, our cohort study suggests that pretreatment baseline DLL3 expression on CTCs is highly predictive of response to tarlatamab.

Of the 20 cases in cohort A, 18 had archival formalin-fixed, paraffin-embedded (FFPE) diagnostic biopsies available for analysis. Consistent with prior studies (8), IHC staining for DLL3 in these specimens was poorly predictive of clinical response (Supplementary Table S4). As noted above, the predictive value of diagnostic tissue–based IHC may be limited by both biological and technological factors compared with IF-based single-cell imaging of CTCs.

### CTC Markers of Tumor Lysis following Tarlatamab Treatment

**Figure 4.**
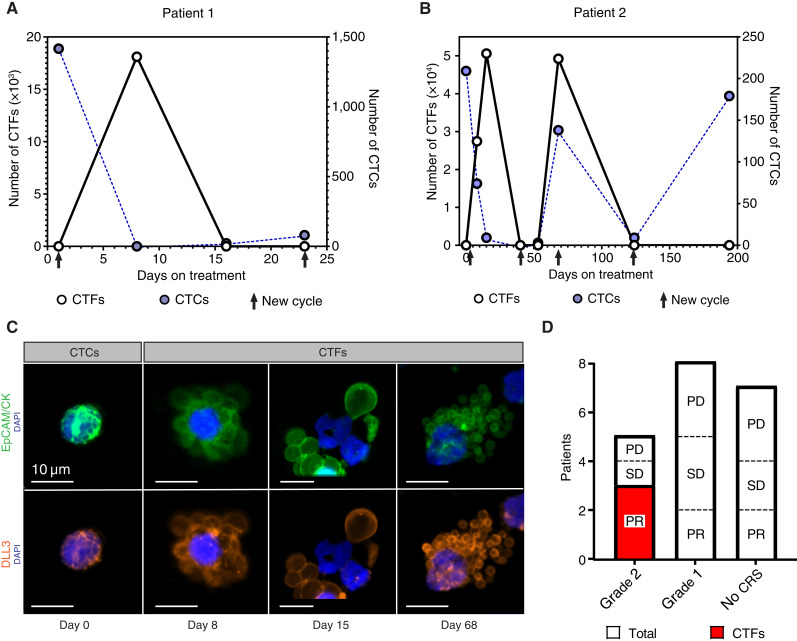
CTFs in the blood of patients with SCLC with CRS following tarlatamab treatment. **A** and **B,** Quantitation of CTFs (black; left *y*-axis) and intact CTCs (blue; right *y*-axis) for patient 1 (**A**) and patient 2 (**B**) over longitudinal treatment course (arrow heads: tarlatamab cycles). **C,** Representative IF images of a CTC (pretreatment, day 0) and CTFs (days 8 through 68) from patient 2, following staining for epithelial markers (green: EpCAM, pan-CK, and CK19) and DLL3 (orange). The staining panel also includes nucleus marker DAPI (blue) and the hematopoietic markers CD45, CD66b, and CD16 (red, negative control). CTFs include single cells with fragmented cytoplasm, extruding nuclei, and anucleated clusters of cellular remnants, staining for either DLL3, epithelial markers, or both. **D,** CRS severity and association with CTFs. Bar plot shows the distribution of patients with or without CTFs stratified by CRS grade. Additionally, the number of patients with PR, SD, and PD in each CRS grade is labeled. Among patients with grade 1 CRS (*N* = 8), none exhibited detectable CTFs. In contrast, three patients demonstrated the presence of CTFs in the grade 2 CRS group (*N* = 5).

One of the primary complications of tarlatamab treatment is CRS, which presents as an inflammatory reaction including fever, hypotension, hypoxemia, and, more rarely, mental status changes (8, 10). Low-grade CRS may occur in as many as half of patients treated with tarlatamab, but all treated patients are currently hospitalized for observation for 24 hours following the first two therapeutic infusions, given the unpredictable risk of high-grade CRS. Of the 20 originally enrolled patients in cohort A, 13 experienced CRS, which was characterized as grade 1 in 8 (40%) patients and up to grade 2 in 5 (25%) patients. In three of the five patients with grade 2 CRS, blood samples taken following tarlatamab infusion (C1D2, C1D8, and C1D15) contained large numbers of cellular fragments [which we refer to as circulating tumor fragments (CTF)] staining for epithelial markers and DLL3 (Fig. 4A–C; Supplementary Fig. S13). Notably, all three of these patients (patient 1, 2, and 8) had a PR on tarlatamab. The two other patients who had grade 2 CRS but did not have CTFs in the blood also did not have tumor shrinkage on tarlatamab (patient 16: SD and patient 39: PD; Fig. 4D). None of the eight patients who had less than grade 2 CRS showed CTFs in blood samples taken at the same intervals after treatment. Thus, in this small cohort, the transient appearance of CTFs in the blood was only evident in patients who had both grade 2 CRS and evidence of tumor response (PR). When present, CTFs outnumber pretreatment CTCs by 10- to 1,000-fold; CTFs include single-tumor cells extruding their nucleus, anucleated single cells, clusters of cells, and cell remnants. We hypothesize that they represent massive tumor cell killing within tissues by recruited T cells, with spilling of cancer cell fragments into the vasculature. The timing of CTF spilling into the bloodstream is shown in two representative cases: In patient 1, who had both grade 2 CRS and immune effector cell–associated neurotoxicity syndrome (ICANS), up to 50,000 individual cell fragments were imaged at a time that closely followed the initial tarlatamab-induced drop in CTCs (Fig. 4A). Patient 2, who also had grade 2 CRS, underwent a cyclical increase and decrease in CTC numbers, timed to each of the serial tarlatamab infusions, with a near-synchronous shedding of CTFs (Fig. 4B; Supplementary Fig. S14). Thus, in a subset of patients, early monitoring of CTCs following initiation of tarlatamab therapy indicates a very rapid targeting and killing of cancer cells by activated immune cells, which may be linked to systemic toxicity.

### Loss or Persistence of DLL3 Expression on CTCs at Disease Progression on Tarlatamab

**Figure 5.**
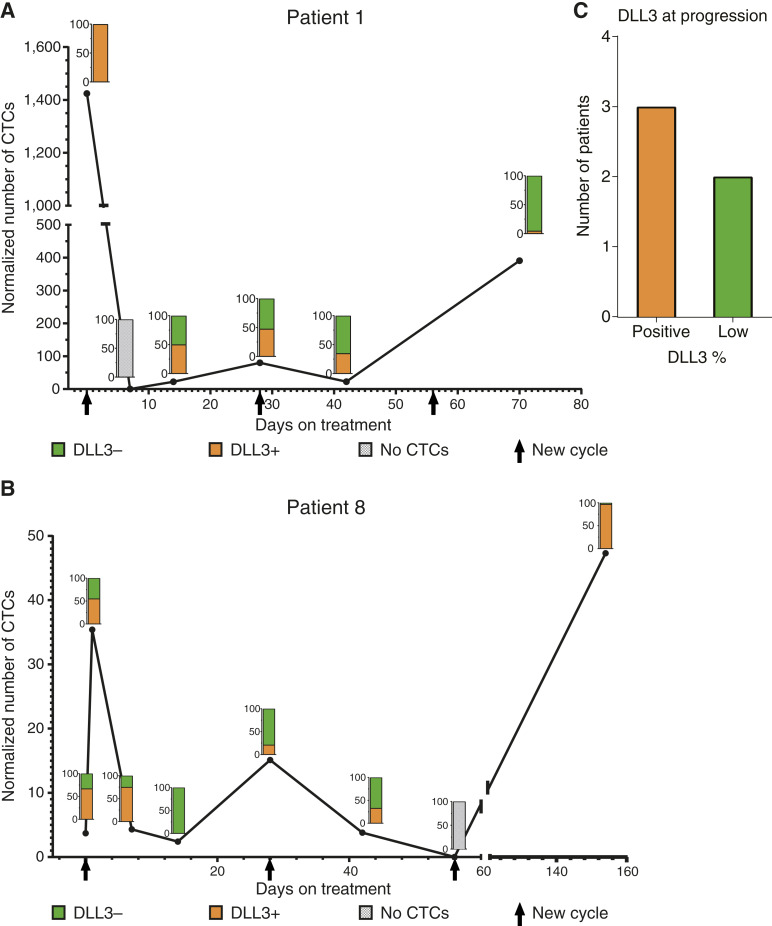
Differential CTC expression of DLL3 upon acquired resistance to tarlatamab. **A,** Representative case (patient 1) showing total CTC enumeration and DLL3 staining in a DLL3^High^ patient with initial profound response to tarlatamab, whose CTCs show complete loss of expression of the targeted epitope at the time of acquired resistance and disease progression. CTC counts are normalized to 20 mL, with the percentage DLL3-positive (orange) versus DLL3-negative (green) shown for each time point during the course of treatment (arrowheads: tarlatamab cycles). **B,** Another representative tarlatamab-responsive DLL3^High^ case (patient 8) in which increasing numbers of CTCs at the time of acquired drug resistance show universally persistent expression of the targeted DLL3 epitope. **C,** Relative fraction of DLL3^Pos^ patients who initially benefited from tarlatamab (PR or SD), whose CTCs at the time of cancer progression are either above (positive) or below (low) the median 25% cutoff for DLL3 expression (*N* = 5).

Although some patients may derive durable benefit from tarlatamab, approximately half of responders will have a duration of response less than 9 months (8). Although our study was not designed to comprehensively address acquired resistance to tarlatamab, we analyzed CTCs at the time of radiographic progression in evaluable patients who initially responded and subsequently progressed on tarlatamab in cohort A. Our analysis suggests two distinct mechanisms of resistance, as reflected by loss or persistence of DLL3 expression. Two patients illustrate these distinct outcomes: Both patient 1 and patient 8 had large numbers of CTCs at the pretreatment baseline, which were 100% and 75% DLL3-positive, respectively (Fig. 5A and B). After a precipitous on-treatment decline, CTCs remained low for approximately 40 and 50 days, respectively, before increasing in number at the time of disease progression. For patient 1, 95% of CTCs at disease progression had become DLL3-negative, consistent with epitope downregulation as a mechanism for treatment failure (Fig. 5A). In contrast, the increasing CTCs in patient 8 at the time of treatment failure were near universally DLL3-positive (98%; Fig. 5B). In this case, failure of immune cell killing despite persistent DLL3 expression may explain acquired resistance. In total, of the 5 evaluable DLL3^Pos^ patients who experienced initial clinical benefit on tarlatamab and subsequently developed disease progression, 3 (60%) retained DLL3 expression in the setting of acquired drug resistance and 2 (40%) showed loss of epitope expression (Fig. 5C).

### T-cell Dysfunction in Patients with Persistent DLL3-Positive CTCs at Progression

**Figure 6.**
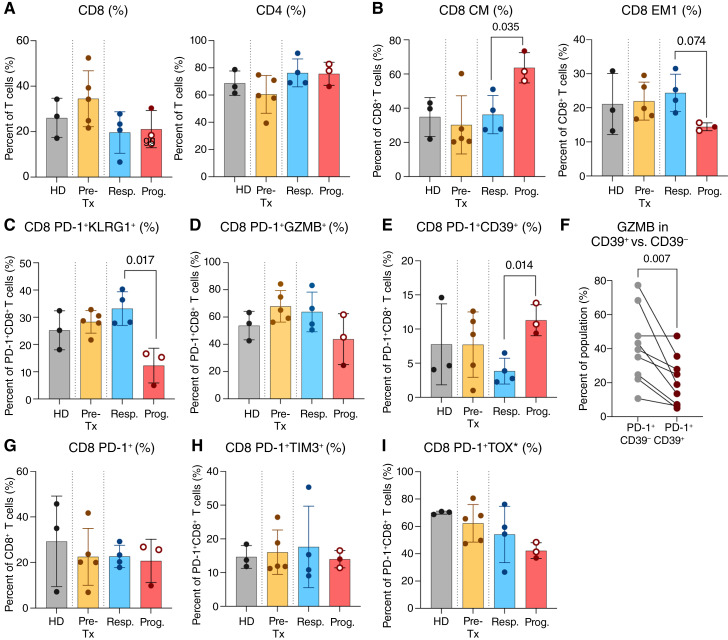
Markers of T-cell dysfunction in patients with acquired tarlatamab resistance despite persistent DLL3 expression. **A,** Box plot representing flow cytometric analysis of PBMCs showing the percentage of CD8^+^ (left) or CD4^+^ (right) live T cells. Blood specimens analyzed from healthy donors (HD: gray, *N* = 3), tarlatamab-untreated advanced SCLC (Pre-Tx: orange, *N* = 5), SCLC cases at the time of tarlatamab response (Resp.: blue, *N* = 4), and patients with SCLC with acquired resistance to tarlatamab despite persistent DLL3 expression in CTCs (Prog.: red, *N* = 2, open circles denote two distinct measurements from one case). For **A–E** and **G–I,** multiple measurements from a single patient (where available) were averaged for statistical comparison using the Student unpaired *t* test between Resp. vs. Prog. groups. **B,** Percentage of central memory (CM) CD8^+^ T cells (phenotype: CD45RA^−^CCR7^+^CD28^+^) versus effector memory subtype 1 (EM1) CD8^+^ T cells (phenotype: CD45RA^−^CCR7^−^CD28^+^). **C,** Percentage of KLRG1^+^ cells within activated (PD-1^+^) CD8^+^ T cells. **D,** Percentage of GZMB^+^ cells within activated (PD-1^+^) CD8^+^ T cells. **E,** Percentage of CD39^+^ cells within activated (PD-1^+^) CD8^+^ T cells. **F,** Percentage of GZMB^+^ cells within PD-1^+^CD8^+^ T cells, with and without CD39 expression, across all SCLC samples. Statistical comparison between CD39^+^ (dark red) and CD39^−^ (gray) cells was done using the Student paired *t* test (*N* = 9 SCLC cases, including Pre-Tx, Resp., and Prog.). Samples with fewer than 50 recorded T cells in either the CD39^+^ or the CD39^−^ group were excluded. **G,** Percentage of PD-1^+^ cells within total CD8^+^ T cells. **H,** Percentage of TIM3^+^ cells within activated (PD-1^+^) CD8^+^ T cells. **I,** Percentage of TOX^+^ cells within activated (PD-1^+^) CD8^+^ T cells.

Given the persistence of DLL3-positive CTCs at the time of progression in a subset of patients with acquired resistance to tarlatamab, we tested peripheral blood mononuclear cells (PBMC) for evidence of T-cell dysfunction. In addition to patient 8, we studied PBMCs from patient 2, with DLL3-positive tarlatamab progression (measured at two time points, 32 days apart), comparing them with samples collected from four different patients during tarlatamab response (patients 17, 28, 29, and 30), five pretreatment blood samples from unrelated patients with advanced SCLC, and three specimens from age-matched (>50 years) healthy blood donors. We performed flow cytometric analysis of PBMCs using a panel designed to detect differences in T-cell memory subsets, markers of functionality and cytotoxicity, and exhaustion-associated markers (Supplementary Figs. S15 and S16). The CD4^+^-to-CD8^+^ ratio was relatively stable across all cases, independent of treatment or progression, but we observed a marked redistribution of T-cell memory subsets in both the CD4 and CD8 compartments (Fig. 6A). Compared with both untreated controls and tarlatamab-responding patients, patients with DLL3-positive PD had a significant increase in central memory (CD45RA^−^CCR7^+^CD28^+^) CD8^+^ T cells (*P* = 0.035), coupled with a trend toward reduction in effector memory (CD45RA^−^CCR7^−^CD28^+/−^) CD8^+^ T cells (*P* = 0.074), especially in the CD8^+^ compartment (Fig. 6B; Supplementary Fig. S15). Central memory T cells have lower immediate effector function potential, including cytokine expression and direct cytotoxicity, than effector memory T cells, suggesting that T cells from these patients have diminished capacity to clear tumor cells. Consistent with this, activated T cells from DLL3-positive progressing cases were less effector-like in their surface marker expression, including decreased expression of KLRG1, a marker of terminally differentiated effector cells, on both activated (PD-1^+^) CD4^+^ and CD8^+^ T cells (Fig. 6C; Supplementary Fig. S15). Moreover, CD8^+^ T cells in these cases had a tendency toward reduced expression of granzyme B (GZMB), the key cytotoxic molecule used by CD8^+^ T cells to kill target tumor cells, although this did not reach significance in this limited sample set (Fig. 6D).

Although we observed evidence of T-cell dysfunction in DLL3-positive cases progressing on tarlatamab, this did not extend to classical markers of T-cell exhaustion. CD39, which plays a critical role in generating immunosuppressive adenosine in the tumor microenvironment, was increased in CD8^+^ T cells from DLL3-positive progressing cases (Fig. 6E; Supplementary Fig. S17A–S17C). CD39^+^ T cells across all patients with SCLC also had significantly lower levels of expression of the cytotoxic GZMB molecule relative to their activated PD-1^+^CD39^neg^ counterparts (Fig. 6F). However, we did not observe increased expression of other classical markers of T-cell exhaustion, such as PD-1, TIM3, or TOX, in DLL3-positive progressing cases (Fig. 6F–I; Supplementary Fig. S17D–S17F). Taken together, these observations suggest that T cells from patients progressing on tarlatamab despite preserved DLL3 expression on tumor cells do not exhibit classical immune exhaustion, but rather, they exhibit an alternate form of dysfunction, with a shift from classical effector phenotypes to suppressed, memory-like features. Persistent DLL3 expression in patients with acquired resistance to tarlatamab associated with T-cell dysfunction raises the possibility that either orthogonal DLL3-targeting approaches (e.g., DLL3 radioligands) or other immunomodulatory strategies might restore T-cell killing (40).

### Persistent Expression of Other Neuroendocrine Markers following Downregulation of DLL3

**Figure 7.**
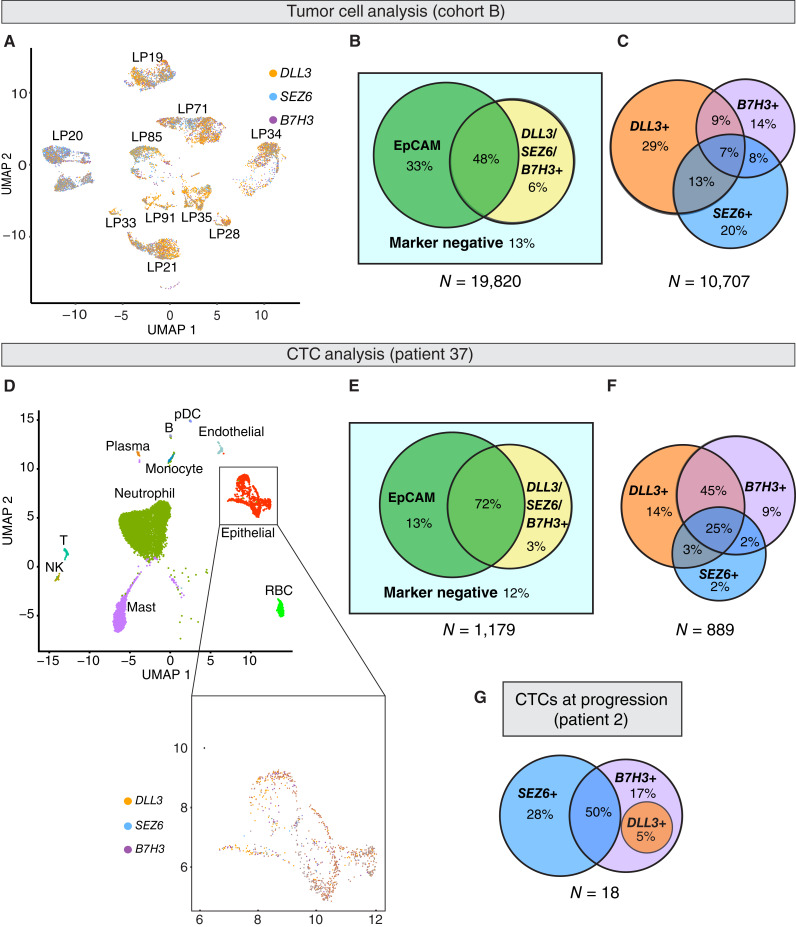
Coexpression of targetable SCLC epitopes *DLL3*, *SEZ6*, and *B7H3* in individual tumor cells and CTCs. **A,** Uniform Manifold Approximation and Projection for Dimension Reduction (UMAP) of 10× scRNA-seq data from SCLC tumors (cohort B), showing independent clustering of each of 10 tumors, with each tumor having cells expressing *DLL3* (orange), *SEZ6* (blue), and *B7H3* (CD276; purple). Single-tumor cells within SCLC biopsies were first identified by RNA-inferred CNV, followed by scoring for expression of the three epitopes. **B,** Venn diagram including all CNV-confirmed cancer cells within the 10 tumors (*N* = 19,820 cancer cells), showing the distribution of single cells expressing EpCAM only (green) versus any of the three epitopes *DLL3*, *SEZ6*, or *B7H3* (yellow) or the intersection of cells expressing both epithelial and targeted epitopes. CNV-confirmed cancer cells expressing neither epithelial markers nor targetable epitopes are shown in light blue. **C,** Venn diagram of CNV-confirmed cancer cells within the 10 SCLC tumors with expression of any of the targetable epitopes showing the overlap in single-cell expression for each of the three epitopes, *DLL3* (orange), *SEZ6* (blue), and *B7H3* (purple). Numbers represent all cancer cells across the 10 tumors, with individual per-tumor comparisons shown in Supplementary Figs. S18 and S19. **D,** UMAP of 10× scRNA-seq of blood cells (patient 37), following microfluidic depletion of hematopoietic cells. CTCs, identified by RNA-inferred CNV, are shown in red, alongside contaminating blood cells (neutrophils, monocytes, mast cells, B cells, plasma cells, plasmacytoid dendritic cells (pDC), endothelial cells, and RBCs). The inset shows magnification of the CTC clustering, with colored expression denoting the targetable epitopes *DLL3*, SEZ6, and *B7H3*. **E,** Venn diagram of all CNV-confirmed CTCs from patient 37 (*N* = 1,179 CTCs), showing the distribution of expression of epithelial markers (green) versus any of the three targetable markers (yellow). CTCs expressing neither of these markers are shown in light blue. EpCAM RNA expression is detectable in most CTCs using RNA-seq, whereas it is below detection using antibody staining for protein expression. **F,** Venn diagram showing the overlap in single-cell expression of *DLL3*, *SEZ6*, and *B7H3* among CTCs from patient 37. **G,** 10× RNA-seq analysis of 18 CTCs expressing any targetable epitope from patient 2, drawn at the time of acquired tarlatamab resistance and disease progression. The Venn diagram confirms loss of *DLL3* expression in this DLL3^High^ patient, with only one remaining *DLL3*-positive CTC, but other CTCs expressing either *SEZ6*, *B7H3*, or both. LP, Lung Patient.

In the subset of cases demonstrating downregulation of DLL3 expression on CTCs upon acquired resistance to tarlatamab, we sought to determine whether the effect is limited to this epitope or, alternatively, whether it may represent a broader alteration in cell differentiation resulting in downregulation of multiple other lineage-associated and potentially targetable epitopes. In addition to DLL3, the neuroendocrine cell surface epitope SEZ6 is under investigation for antibody targeting in SCLC (NCT03639194), as is the immune checkpoint protein B7H3/CD276 (hereafter B7H3; NCT06498479 and NCT05280470), but the pattern of expression of these targetable epitopes compared with DLL3 within single SCLC tumor cells has not been defined. To first determine the extent of overlap between these three epitopes within single SCLC tumor cells, we interrogated the 10 biopsies that we had subjected to 10× RNA-seq (cohort B; Fig. 7A). Across these tumor specimens, we used RNA-seq–inferred CNV as a marker of aneuploidy to separate single cancer cells from reactive stromal cells, revealing that a median of 54% of all CNV-confirmed single cancer cells express any of the three epitopes (Fig. 7B; Supplementary Data S2). Among these, a median of 35% of single SCLC cells express *DLL3* compared with 18% for *SEZ6* and 17% for *B7H3* (Fig. 7C; Supplementary Fig. S18; Supplementary Data S2). Only a median of 28% of *DLL3*-positive cells coexpress the second neuroendocrine marker SEZ6, 28% of *DLL3*-positive cells also have *B7H3* RNA, and only 9% coexpress all three markers together (Fig. 7C; Supplementary Fig. S19; Supplementary Data S2). Comparable results are produced by reanalysis of a previously published dataset (34), which has a higher fraction of *DLL3*-positive cells (median 64% of cancer cells; Supplementary Data S2; Fig. 2A and B). Analyses of overlapping expression patterns for the three epitopes are presented both within individual tumors and across the entire pooled dataset (Fig. 7B and C; Supplementary Figs. S18–S21; Supplementary References). Taken together, the large fraction of single SCLC cells with nonoverlapping expression patterns raises the possibility of selectively targeting each of the three epitopes, either simultaneously or sequentially.

To extend these observations to CTCs, we obtained 20 mL blood specimens from two patients prior to tarlatamab treatment (patient 36 and patient 37; Supplementary Fig. S22A–S22C; Fig. 7D, respectively), depleted hematopoietic cells using the CTC-iChip, and subjected the CTC-enriched cell population to 10× RNA-seq. The application of 10× RNA-seq to microfluidically enriched CTCs is more challenging than the analysis of tumor biopsies, given the small number of isolated CTCs admixed with abundant contaminating leukocytes. Nonetheless, using RNA-based inferred CNV to genetically identify aneuploid CTCs within the microfluidically enriched cell population, we annotated 1,179 CTCs in a case with abundant CTCs (patient 37), totaling 8% of all cells in the enriched product (Fig. 7D). Of all CNV-confirmed CTCs in this case, 75% were positive for any of the three markers, *DLL3*, *SEZ6*, and *B7H3* (Fig. 7E), and among these positive cells, 772 (87%) were positive for *DLL3* transcripts, 280 (32%) were positive for *SEZ6*, and 728 (81%) were positive for *B7H3.* Coexpression of both *DLL3* and *SEZ6* was present in 28% of CTCs, coexpression of *DLL3* and *B7H3* in 70% of CTCs, and all three epitopes were coexpressed in 25% of CTCs (Fig. 7F). Comparable results were observed in CTCs from patient 36, which were fewer in total number (Supplementary Fig. S22A–S22C)***.*** Taken together, both tumor and CTC-based single-cell analyses identify DLL3, SEZ6, and B7H3 as targetable epitopes that are only partially overlapping in expression across SCLC tumor cells.

Given the absence of detectable EpCAM/CK protein staining in a subset of DLL3-positive CTCs (Fig. 1D), we also interrogated the 10× scRNA-seq CTC data for coexpression of DLL3 and epithelial markers, comparing them with SCLC tumor specimens. Across all CNV-confirmed CTCs from patient 37, isolated through microfluidic depletion of hematopoietic markers and without bias for epitope expression or cell size, 65% expressed *DLL3* and either *EpCAM* or *CK* (*CK 4*, *5*, *6*, *8*, *10*, *13*, and *18*) transcripts, whereas 27% expressed at least one of these epithelial markers without *DLL3* RNA. Only 0.3% of CTCs had *DLL3* transcripts in the absence of any *EpCAM* or *CK* RNA (Supplementary Fig. S23A–S23C). A comparable distribution was noted from scRNA-seq analysis of SCLC tumors from cohort B and cohort C (Supplementary Fig. S23). In addition, almost all DLL3-positive cells are indeed epithelial cells in patient 37 and tumors in cohort B (Supplementary Fig. S24A and S24B). Thus, together with the InferCNV data, the presence of even rare EpCAM and CK transcripts confirms that all *DLL3*-expressing CTCs are of epithelial cancer cell origin. Low EpCAM and CK mRNA expression in a fraction of CTCs may render these markers undetectable with the less sensitive IF protein staining assays used to score CTCs, explaining the large fraction of DLL3-positive but EpCAM/CK-negative CTCs.

Finally, to determine whether the tarlatamab-induced loss of DLL3 expression is specific to that epitope or extends to other neuroendocrine epitopes, we analyzed patient 2 (cohort A), who had sufficient CTCs at the time of acquired drug resistance to allow for 10× RNA-seq following microfluidic CTC enrichment. Consistent with the absence of DLL3 protein expression on CTCs when this patient developed clinical progression, *DLL3* RNA expression was virtually abrogated, with only 1 CTC-positive for *DLL3* transcript of 21 CTCs (5%; Supplemental Fig. S12). However, 18 of the 21 (86%) CTCs at progression expressed either *SEZ6* or *B7H3*, with 28% of these CTCs having only *SEZ6*, 17% only *B7H3*, and 50% expressing both *SEZ6* and *B7H3* (Fig. 7G). Thus, the downregulation of *DLL3* expression at the time of acquired resistance to tarlatamab in this patient was not accompanied by suppression of other neuroendocrine or targetable epitopes, supporting the possibility of alternative antibody targeting.

## Discussion

The neuroendocrine epitope DLL3 has emerged as a compelling target for SCLC therapeutics, due to its high expression on the surface of tumor cells and its minimal expression in normal tissues. Earlier-generation DLL3-targeted therapies, such as the antibody–drug conjugate (ADC) rovalpituzumab tesirine, showed limited efficacy and intolerable toxicity in clinical trials (41–44). In contrast, the DLL3 bispecific antibody tarlatamab has demonstrated both compelling efficacy and tolerability, leading to accelerated FDA approval for treatment-refractory SCLC (8). Nonetheless, fewer than half of patients with SCLC derive durable clinical benefit from tarlatamab, and in the absence of a predictive biomarker all patients are treated empirically. Our finding that CTC expression of DLL3 is predictive of clinical response in advanced SCLC offers a path toward patient stratification for this potentially effective treatment.

DLL3 expression is not universal across neuroendocrine cancers, emphasizing the need for an individualized pretreatment analysis at single-cell resolution. Among liquid biopsies, isolation of CTCs enables protein quantification, which is not possible using ctDNA-based assays, and it requires SCLC cell enrichment strategies from blood specimens that do not rely on expression of the epithelial surface marker EpCAM, which is variable in SCLC. Similarly, CTC enrichment from patients with SCLC cannot rely on the presumed larger diameter of cancer cells compared with leukocytes, as small cell size is a defining characteristic of this neuroendocrine cancer. The microfluidic hematopoietic cell depletion platform applied here is therefore well suited for SCLC, enriching CTCs based on the absence of hematopoietic markers (i.e., negative depletion), without bias for either tumor marker or cell size. This strategy allows for 10^4^ enrichment of CTCs from a blood specimen through removal of normal blood cells, followed by simultaneous staining for both the targeted DLL3 epitope and epithelial markers. Compared with most solid tumors, SCLC displays high intravascular invasiveness and metastatic propensity, associated with a high number of CTCs in the bloodstream. Cell-based liquid biopsies may thus guide more precise, protein-targeted immunologic therapies in SCLC, much as ctDNA blood analyses have helped shape the application of genetically targeted, small-molecule therapies in NSCLC.

SCLC was initially thought to have virtually universal DLL3 expression, and indeed standard IHC measurements of diagnostic biopsies score greater than 90% of cases as positive (6, 11). However, through scRNA-seq of tumor cells, we find significant heterogeneity, with a range extending from 8% to 90% of cancer cells within individual tumors being *DLL3*-positive. This *DLL3*-positive cancer cell fraction seems to be stable across different molecularly defined subtypes of SCLC, and it does not differ between treatment-naïve cases and those that have progressed on chemotherapy and ICIs. Most importantly, when analyzing CTCs from patients with treatment-refractory SCLC who are eligible for tarlatamab, we find clear stratification into cases being either DLL3^Pos^ or DLL3^Low^, rather than the more uniform distribution in epitope expression evident in primary diagnostic tumor biopsies. This apparent biphasic pattern and its strong correlation with clinical response to tarlatamab suggests that CTCs analyzed at the time of treatment initiation may represent a more therapeutically relevant cell population than is found in primary diagnostic tumor biopsies. Applying the numerical cutoff of 25% DLL3-positive CTCs, we find that 11 of 11 (100%) DLL3^Pos^ patients show clinical benefit (6 PR and 5 SD) from tarlatamab, with all the PR cases having >75% DLL3 positivity (DLL3^High^) scores. In contrast, of the 9 DLL3^Low^ patients, only 2 (22%) derived clinical benefit from tarlatamab (1 PR and 1 SD), with the PR case having an unusual *DLL3* RNA splicing pattern that precluded diagnostic scoring without affecting therapeutic response. If confirmed in larger studies, these findings may provide a critical tool for patient stratification prior to therapy with DLL3-targeting antibody-based therapies. This may be particularly important given ongoing efforts to move tarlatamab from the second-line to first-line setting in ES-SCLC (e.g., NCT06211036), as well as current studies testing tarlatamab in limited-stage SCLC (NCT06117774).

We note that our cohort excluded patients with brain metastases as tarlatamab may have different levels of activity in systemic versus CNS disease, and addressing its efficacy in brain metastases remains a critical challenge. Importantly, DLL3 is a targetable cell surface epitope shared by multiple cancers that exhibit neuroendocrine differentiation, including subsets of endocrine and gastrointestinal tumors, treatment-induced lineage transformation in NSCLC, and aggressive transformation in prostate cancer. Tarlatamab remains investigational for these additional indications, and further CTC analyses will be required, both to ascertain the pattern of DLL3 expression in these cancers and its correlation with clinical response.

An unexpected finding in our closely timed longitudinal analysis of blood specimens following tarlatamab treatment was the transient appearance of massive numbers of CTFs in the blood, coinciding with the expected peak of tumor cell killing and the onset of CRS. CTFs, numbering up to 50,000 individual imaging events within 20 mL of blood, vastly outnumber CTCs and seem to peak shortly after the treatment-induced decline in CTCs. We postulate that CTFs represent necrotic tumor cells shed into the bloodstream, at a time when the systemic tumor burden is very high and initial doses of tarlatamab trigger massive T-cell killing of SCLC. As CTFs begin to accumulate as early as the second day following tarlatamab initiation, further studies will be required to determine whether their appearance is predictive of severe CRS and the need for inpatient hospitalization.

Given the variable duration of response in patients benefiting from tarlatamab, we also studied mechanisms of treatment failure. In patients who experienced a profound initial response followed by acquired resistance, we observed two divergent scenarios. In a subset of cases, strong DLL3 expression in pretreatment CTCs gave way to DLL3-negative CTCs at the time of disease progression on therapy, consistent with epitope downregulation. In one such case in which drug-resistant CTCs were sufficiently abundant for analysis by 10× scRNA-seq, we found persistent expression of another neuroendocrine marker, SEZ6, suggesting that loss of DLL3 may be specific to that epitope and hence not accompanied by a broader lineage-specific loss of other neuroendocrine markers. Indeed, single-cell analysis of SCLC tumors as well as SCLC CTCs indicates that two other epitopes currently under therapeutic investigation for SCLC, SEZ6, and B7H3 have varied overlap in expression across individual cancer cells. Whether sequential or simultaneous targeting of distinct markers in SCLC leads to optimal clinical responses remains to be seen in future trials.

In another subset of patients with DLL3-positive CTCs who experienced a profound initial response to tarlatamab, we find that disease progression occurs despite continued DLL3 expression by tumor cells. In such cases, characterization of peripheral blood T cells shows a consistent dysfunctional pattern, with reduced expression of effector markers and increased memory phenotypes. This alteration in T-cell differentiation and tumoricidal potential differs from the characteristic T-cell exhaustion phenotype, but it is consistent with prior reports on chimeric antigen receptor (CAR)-T cell failure. In the KTE-X19 trial of CD19-specific CAR-T cells in mantle cell lymphoma, 93% of patients had detectable CD19 at relapse, suggesting that T-cell dysfunction was a key contributor to treatment failure (45). Conversely, the expansion of effector T cells expressing cytotoxic molecules over central memory populations was associated with better response in the landmark ZUMA-1 trial of CD19 CAR-T cells in diffuse large B-cell lymphoma (46). Elevated CD39 expression, as observed in our tarlatamab-refractory patients, has also been associated with T-cell dysfunction in CAR-T cells (47, 48) and in the settings of chronic viral infections (49, 50) and advanced solid tumors (51, 52). Although CD39 expression is well defined in classically exhausted T cells, a distinct dysfunctional CD39^+^ T-cell population lacking other canonical features of exhaustion has been associated with poor CAR T-cell expansion and disease progression in acute myeloid leukemia (53, 54). We note that our studies were limited to systemic alterations evident in PBMCs and did not analyze immune infiltrates within the tumor microenvironment. Further studies will be required to determine whether this mechanism of immune dysfunction following response to tarlatamab is reversible through coadministration of immune-stimulatory therapies. In addition, nonimmunologic therapies that target DLL3, such as radioligands or ADCs, may constitute an alternative therapeutic approach in patients with persistent tumor expression of DLL3 associated with immune dysfunction.

Our study has several limitations. This prospectively collected single-institution study will need to be validated in a larger multi-institutional trial to confirm the predictive value of DLL3 scoring in CTCs and optimize scoring cutoffs in SCLC. As future trials seek to integrate tarlatamab treatment within first-line therapy for SCLC, scoring of CTCs for DLL3 expression will also need to be tested as a predictor of clinical outcomes to alternative regimens. Beyond SCLC, the utility of DLL3 staining on CTCs in common cancers that may acquire neuroendocrine features, such as prostate cancer, will need to be ascertained. The CTC isolation and imaging technology deployed here is for research use only, and it will need to be further standardized for broad clinical deployment. Given the small number of cases studied here, the clinical significance of CTF detection as a marker for tumor lysis and CRS risk will need to be confirmed, as will the altered T-cell functional markers that we observed in the small number of patients with acquired tarlatamab resistance despite persistent DLL3 CTC expression. Finally, although our studies of acquired tarlatamab resistance raise the possibility of targeting other neuroendocrine epitopes in SCLC that have lost DLL3 expression or of deploying immune-independent theranostics in those with persistent DLL3 expression, these approaches will need to be tested in DLL3-informed clinical trials.

In summary, we report the clinical application of CTC-based quantitation for DLL3 expression in advanced SCLC, both in stratifying patients likely to respond to the bispecific antibody tarlatamab and in dissecting distinct mechanisms of acquired resistance that may lead to different therapeutic considerations. For immune-based cancer therapies that are uniquely dependent upon epitope expression by cancer cells, CTC-based measurements thus provide a robust noninvasive measurement that may help guide therapeutic interventions.

## Methods

### Patient Cohort and Sample Collection

We identified patients with biopsy-confirmed SCLC treated with tarlatamab at Massachusetts General Hospital (MGH) between July 1, 2024, and June 30, 2025. We analyzed patient demographics, treatment history, and clinical outcomes. Clinical response criteria were assessed as per RECIST version 1.1 (32). Peripheral blood samples were collected on C1D1, C1D2, C1D8, C2D1, and C2D15 and subsequently at time points associated with each restaging scan from patients using one or two vacutainers, filled either with ethylenediaminetetraacetic acid (EDTA) or Streck preservative. CTC enrichment and analysis were performed as outlined in the methods below. All patient data and samples were collected following written informed consent from patients, and studies were conducted in accordance with recognized ethical guidelines (US Common Rule). The study was approved under an Institutional Review Board (IRB)–approved protocol (DF-HCC protocol 13-416).

### IHC Staining

We performed IHC analysis for DLL3, ASCL1, NeuroD1, and POU2F3 in all cases where tissue was available (with at least 100 tumor cells on each slide) using laboratory-developed tests validated in a Clinical Laboratory Improvement Amendments–certified laboratory. IHC was performed on 5-micron FFPE tissue sections using an automated staining system BOND Rx or BOND III (Leica Biosystems) and BOND Polymer Refine Detection Kit (Leica Biosystems, DS9800) for the following targets (clone, vendor, catalog number, and dilution): ASCL1 (rabbit monoclonal, E5S4Q; Cell Signaling Technology; 10585; 1:100), NeuroD1 (rabbit monoclonal, EPR17084; Abcam; ab205300; 1:50), POU2F3 (rabbit monoclonal, E5N2D; Cell Signaling Technology; 36135; 1:500), and DLL3 (rabbit monoclonal, SP347; Ventana/Roche; 790–7016; prediluted). Antigen retrieval was based on EDTA-based pH 9 epitope retrieval buffer ER2 (Leica Biosystems) for 40, 40, 40, and 80 minutes for ASCL1, NeuroD1, POU2F3, and DLL3, respectively; the duration of the primary antibody incubation was 30 minutes each. The protocol (including antibody clone, dilution/incubation, and antigen retrieval) in this study was comparable with published protocols (12, 37). For ASCL1, NeuroD1, and POU2F3, nuclear immunoreactivity was considered; for DLL3, partial or diffuse cytoplasmic and/or membranous immunoreactivity was considered positive. IHC expression of ASCL1, NeuroD1, POU2F3, and DLL3 was evaluated blindly by an attending pathologist using an *H*-score system (0–300), with staining intensity (range 0–3; 0: absent; 1: weak; 2: medium; 3: strong) multiplied by the percentage of positive cells (0%–100%).

### CTC-iChip Fabrication and Sample Processing

CTC-iChip microfluidic devices were produced from medical-grade cyclic olefin copolymer using variotherm injection molding by Stratec Biomedical AG for CTC isolation through a pressure-driven workflow. Blood samples were received within 6 hours of collection and processed within 24 hours. Sample volumes and complete blood count measurements were recorded prior to processing. Samples then underwent sequential incubation with antibody reagents and magnetic beads, followed by enrichment using the CTC-iChip. A 1:1 mixture of whole blood and buffer, containing magnetically labeled WBCs, was introduced into the microfluidic CTC-iChip under pressure-driven flow. The sample first passed through an array of inertial focusing modules designed to hydrodynamically separate components, effectively diverting smaller cells such as RBCs, platelets, and unbound magnetic beads to waste channels. The remaining population of larger nucleated cells, including CTCs and magnetically tagged WBCs, was inertially focused into a single-file stream for high-resolution separation. This stream was routed through two serial magnetic deflection zones, where external permanent magnets generated spatially varying magnetic fields to selectively remove labeled WBCs from the flow path. The resulting output was an enriched population of unlabeled CTCs, concentrated into a reduced elution volume relative to the input, whereas depleted fractions were directed to waste. Enriched fractions, which still contain a subset of WBCs and RBCs alongside CTCs, were collected on ice to preserve cellular morphology. To standardize downstream cytologic processing, the nucleated cell count was determined for each sample, and the suspension was divided accordingly to plate approximately 100,000 to 300,000 cells per slide. Fixation was performed using 0.5% paraformaldehyde for 10 minutes, followed by cytocentrifugation with Epredia EZ megafunnels on a Thermo Shandon Cytospin 4 at 2,000 rpm for 6 minutes. Instead of the manufacturer’s standard slides, TruBond 380 adhesive slides were used to improve cell adherence. Slides were rinsed briefly with 1× phosphate-buffered saline (PBS), then air-dried to ensure optimal retention of cells, and stored at 4°C for 1 to 2 days prior to immunostaining.

### WBC Depletion Antibody Preparation

A premade antibody cocktail was designed to enable selective depletion of WBCs via magnetic labeling. The cocktail consists of biotinylated mAbs targeting key surface markers, which are diluted in blood to the given concentrations: anti–human CD45 (Thermo Fisher Scientific, clone HI30, IgG1; 0.189 µg/million cells), anti–human CD16 (BD Biosciences, clone 3G8, IgG1; 0.0189 µg/million cells), and anti–human CD66b (Novus Biologicals, clone 80H3, IgG1; 0.0189 µg/million cells). Antibodies were diluted in sterile, 0.22 µm–filtered 1× PBS supplemented with 0.1% (w/v) bovine serum albumin (BSA, Sigma-Aldrich, #A2058) to enhance protein stability and minimize nonspecific binding. The prepared antibody solution was stored at 4°C and used within a 7-day window to ensure consistency in depletion performance.

### Magnetic Bead Preparation

Magnetic 1-µm Dynabeads MyOne Streptavidin T1 beads (Invitrogen) were employed to label WBCs preincubated with biotinylated antibodies. Prior to use, beads were washed 3 times with 0.01% Tween 20 (Thermo Fisher Scientific, BP337-100) in 1× PBS to remove residual storage buffer. Beads were then washed 3 times using 0.1% BSA in 1× PBS solution as a blocking step to reduce nonspecific interactions. After wash steps, beads were resuspended and maintained in 0.1% BSA/PBS at a final concentration of 10 mg/mL (approximately 10 million beads/µL). The prepared bead suspension was stored at 4°C and used within 1 week. In blood, final bead concentrations were titrated to 50 to 100 beads per cell, depending on the specific sample.

### Buffer Preparation

A standardized running buffer was prepared for CTC-iChip operation and associated fluidic processes, including sample dilution and wash steps. The buffer consisted of 0.2% (w/v) Pluronic F-68 (Sigma-Aldrich, cat. #K4894) dissolved in 1× PBS. The solution was mixed until the surfactant was uniformly dispersed and then passed through a 0.22-µm membrane filter (Corning, # 430517) to eliminate particulates and ensure sterility. All buffer preparations were stored at controlled room temperature and used within a 14-day window to minimize performance variability across experimental runs.

### IF Staining

Dried, stored slides were rehydrated with cold 1× PBS and blocked with a solution of 3% BSA, 1% Tween 20, and 2% normal goat serum (Abcam #ab7481) in PBS for 1 hour and stained with EpCAM, pan-CK (CK8/18), and CK19 conjugated to Alexa Fluor 488 (Cell Signaling Technology 5198S, Cell Signaling Technology 4523S, and Thermo Fisher Scientific MA5-18158), pooled WBC markers CD45, CD16, and CD66b conjugated to Alexa Fluor 647 (BioLegend 302020, BioLegend 304020, and BioLegend 305110), DLL3 labeled with Alexa Fluor 555 or Alexa Fluor 594 (Cell Signaling Technology 71804S, Abcam ab150078, and Abcam ab150084), and DAPI (Thermo Fisher Scientific 62248). Supplementary Table S5 lists the working concentration of all antibodies. We tested four concentrations (0.1, 0.25, 0.375, and 0.75 µg/mL) to select the DLL3 antibody concentration. We chose 0.375 µg/mL as the final working concentration, as this concentration yielded high DLL3 signal intensity in the DLL3-positive cell line NCI-H82 while still maintaining minimal background staining in the WBCs. This concentration is also consistent with the manufacturer’s recommended dilution range. Staining for all markers was done for 1 hour, using a solution of 0.1% BSA and 1% Tween 20 in PBS as diluent. Conjugated antibodies, DAPI, and any unconjugated primary antibodies were applied first. After two 5-minute washes, secondary antibodies were applied. Final washing was performed sequentially with 0.3% Tween 20 in PBS and 1× PBS before applying mounting media (Thermo Fisher Scientific #P36962), a coverslip, and curing overnight.

### CTC Enumeration and Marker-Based Classification

Following IF staining, whole-slide imaging was conducted using the Akoya Biosciences PhenoImager at 40× magnification (0.25 µm/pixel resolution). Slides were scanned across channels to capture nuclear and multiplexed immunofluorescent signals. Exposure parameters were predefined using reference scans from healthy donors and spiked-in positive controls to ensure consistency across samples; reduced exposure settings were applied in select cases to mitigate signal saturation, and this was accounted for in scoring. All images were collected in 8-bit format (intensity values ranging from 0 to 255). Images were then processed using the HALO image analysis platform. Nuclear segmentation was performed using DAPI to define individual cells, and a cytoplasmic mask was applied based on the spatial extent of the marker signal. For each cell, fluorescence intensity values within both nuclear and cytoplasmic compartments were extracted for all markers and exported to a structured data matrix for analysis. This dataset was then used to establish empirical marker thresholds and define inclusion/exclusion filters for candidate CTC identification. Candidate CTCs were identified based on empirically defined fluorescence intensity thresholds. Cells were classified as CTCs if they exhibited either (i) high expression of epithelial markers (EpCAM and/or CK) or (ii) elevated DLL3 expression in the absence of EpCAM/CK signal. Cells expressing hematopoietic lineage markers (CD45, CD66b, or CD16) above the negative selection threshold were excluded. Intensity values were normalized for exposure time and averaged over the segmented cell masks to enable cross-sample comparison. Automated preclassification was followed by independent manual review by at least three researchers, who evaluated candidate CTCs based on morphology, marker distribution, and expression intensity. For DLL3 classification, a consensus-based approach was implemented: Cells were considered DLL3-positive if the corresponding fluorescence intensity exceeded a threshold of 20 and exhibited a uniform or biologically plausible distribution of signal across the cytoplasmic mask. This classification method was independently reviewed and validated by three analysts to ensure consistency and reduce subjectivity in marker interpretation. Final CTC counts were normalized to a reference blood volume of 20 mL to account for variation in sample input volume.

### T Cell Flow Cytometry

PBMCs from patients with SCLC and healthy donors were thawed rapidly in warm T-cell culture medium (RPMI +10% FBS) containing DNase I (50 μg/mL; Sigma-Aldrich) to break up clumps of dying cells. Cells were counted, and approximately 2 million live cells were carried forward for staining. Cells were stained for surface markers for 22 minutes at room temperature in Brilliant Stain Buffer (BD Biosciences) using the following panel with antibodies from BD Biosciences or BioLegend: Live/Dead Near-IR (Invitrogen, 1:1,000), CD3-BUV737 (BD Biosciences, UCHT1 at 1:100), CD4-BUV496 (BD Biosciences, SK3 at 1:100), CD8-BUV395 (BD Biosciences, RPA-T8 at 1:100), CCR7-PE/Dazzle594 (BioLegend, G043H7 at 1:25), CD45RA-PE/Cy5 (BioLegend, HI100 at 1:50), IL7Ra-BV711 (BioLegend, A019D5 at 1:25), KLRG1-FITC (BioLegend, MAFA at 1:20), CD101-PerCP/Cy5.5 (BioLegend, BB27 at 1:50), PD-1–BV605 (BioLegend, EH12.2H7 at 1:25), TIM3-PE/Cy7 (BioLegend, F38-2E2 at 1:50), CD39-BV510 (BioLegend, A1 at 1:50), LAG3-BV421 (BioLegend, A15153G at 1:25), CD28-BV785 (BioLegend, CD28.2 at 1:50), and CD95-Spark Blue 574 (BioLegend, DX2 at 1:50). Cells were washed 3 times with flow cytometry staining buffer (PBS +0.5% FBS + 2 mmol/L EDTA) and then fixed and permeabilized using the Foxp3/Transcription Factor Staining Buffer set (eBioscience). Cells were stained for the following intracellular targets: TCF1-APC (Cell Signaling Technology, C63D9 at 1:25), TOX-PE (eBioscience, TXRX10 at 1:25), and GZMB–Alexa Fluor 700 (BioLegend, QA16A02 at 1:25). Cells were washed 3 times with 1× permeabilization buffer (eBioscience) and resuspended in flow cytometry staining buffer. Samples were acquired on the Cytek Aurora spectral flow cytometer. All flow data were analyzed using FlowJo version 10.10.0.

### Cohort B SCLC Tumor scRNA-seq Data

Cells were collected from 10 patients with SCLC and sequenced with 10× Genomics Chromium platform. Preprocessing of scRNA-seq data, such as demultiplexing, barcode assignment, and UMI quantification, was done using the Cell Ranger pipeline provided by 10× Genomics. The reads were aligned to the human hg38 reference genome. Quality control was performed with the following metrics: (i) ambient RNA contamination removal by SoupX (v1.6.2; ref. 55); (ii) doublet detection and removal by DoubletFinder (v2.0.4; ref. 56), followed by manual review of predicted doublets on Uniform Manifold Approximation and Projection for Dimension Reduction (UMAP) to avoid false removal of cells heavily biased to a specific cell type; (iii) cell-level metric: cells with the number of genes > 500, UMI counts >1,000, and mitochondrial ratio <0.1 were kept; (iv) gene-level metric: genes detected in ≤3 cells in each sample were excluded. Downstream analyses were performed using the Seurat package [v5.0.1; Klarman Cell Observatory, Broad Institute of MIT and Harvard (cited December 15, 2025). Available at: https://github.com/broadinstitute/inferCNV)].

Marker genes for each cluster were identified using FindAllMarkers in Seurat (v5.01) and nonnegative matrix factorization implemented in GeneNMF (v0.6.2; bioRxiv 2024.05.31.596823), and cell types were then manually annotated based on these marker genes. To identify malignant cells, CNVs of all epithelial cells were first inferred with InferCNV [v1.22.0; Klarman Cell Observatory, Broad Institute of MIT and Harvard (cited 2025 Dec 15). Available from: https://github.com/broadinstitute/inferCNV], using immune cells and stromal cells as normal cells. Total CNV score per cell was calculated as the sum of absolute processed expression value in expr.data of InferCNV output. Subclusters of epithelial cells with low total CNV scores and mixed from multiple tumors were annotated to be normal epithelial cells, and all other epithelial cells were assigned as tumor cells. SCLC subtypes of tumor cells were assigned to the most enriched signature compiled based on four subtypes that have been reported (13, 34, 57), including SCLC-A (*ASCL1*, *SOX4*, *STMN2*, and *DOC2A*), SCLC-N (*NEUROD1*, *ADCYAP1*, *NRXN1*, *SSTR2*, *ID1*, *ID3*, *SST*, and *DLK1*), SCLC-P (*POU2F3*, *ASCL2*, *CD44*, *MYC*, *KIT*, and *YBX1*), and SCLC-I (*Inflamed*, *CD274*, *PDCD1*, *CD80*, *CD86*, *CTLA4*, *CD38*, *IDO1*, *TIGIT*, *ICOS*, *LAG3*, *CCL5*, *CXCL10*, *HLA-A*, *HLA-B*, *HLA-C*, *HLA-E*, *HLA-F*, *HLA-G*, *HLA-DRA*, *HLA-DRB1*, *HLA-DQA1*, *HLA-DMA*, *HLA-DPA1*, *HLA-DMB*, *HLA-DPB1*, *HLA-DQB1*, *HLA-DOA*, and *HLA-DOB*). Signature scores were calculated at the single-cell level using the Mann–Whitney U statistic implemented in AddModuleScore_UCell in the UCell R package (v2.10.1; ref. 58). DLL3-positive cells were defined as tumor cells with at least one UMI count. NOTCH pathway genes, including *NOTCH1*, *NOTCH2*, *NOTCH3*, *NOTCH4*, *HES1*, *HEY1*, *JAG1*, and *JAG2*, were used to estimate false DLL3-negative cells due to technical dropouts. Comparisons between DLL3-positive and DLL3-negative cells were performed and visualized using R packages Seurat (v5.1.0; ref. 59), SeuratExtend (v1.1.6; bioRxiv 2024.08.01.606144), and ggplot2 (v3.5.1; ref. 60). SEZ6- and B7H3-related analyses were performed similar to DLL3.

### Cohort C SCLC Tumor scRNA-seq Data

A public SCLC epithelial scRNA-seq dataset was downloaded from the Human Tumor Atlas Network data portal at https://data.humantumoratlas.org/ (34). Data analyses, including quality control, clustering, cell-type annotation, and malignant cell detection, were described in Chan and colleagues (34). Additional filtering was applied to remove low-capturing cells with the detection of less than 5% of all genes in the data. In total, 49,242 cells from 19 patients were retained for downstream analyses. DLL3-, SEZ6-, and B7H3-related analyses were performed in the same manner as those in cohort B. Pseudobulk RNA-seq analysis was performed using the AggregateExpression method from the Seurat package.

### Cohort B SCLC CTC scRNA-seq Data

CTCs were enriched from the blood of three patients with SCLC using the CTC-iChip followed by magnetic depletion of RBCs. The enriched samples were processed with the 10× Genomics Chromium platform (Chromium GEM-X Single Cell 3' Kit v4) and sequenced on a NextSeq 2000 system with P2 XLEAP-SBS chemistry, targeting 20,000 reads per cell. Preprocessing, quality control, and downstream analyses were performed similarly to scRNA-seq of primary tumors above, except for relaxed cell-level filtering: Cells with a number of genes >300, UMI counts >500, and mitochondrial ratio <0.1 were kept to recover as many cells as possible. See quality control metrics in Supplementary Data S3.

### Statistical Analysis

Pairwise comparisons were performed using the Wilcoxon rank-sum test. For multiple-group comparisons, the Kruskal–Wallis test was applied, and *P* values were adjusted using the Holm correction. Spearman correlation coefficients were calculated to evaluate the dependence of the fraction of DLL3-positive cells on the proportions of each SCLC subtype within tumors.

## Supplementary Material

Supplementary Table S1 Supplementary Table S1 shows patient demographics and clinical characteristics for Cohort A.

Supplementary Table S2 Supplementary Table S2 shows the exclusion criteria for analysis cohort.

Supplementary Table S3 Supplementary Table S3 shows pre-treatment assessments of CTCs for Cohort A.

Supplementary Table S4 Supplementary Table S4 shows the DLL3 IHC data for Cohort A.

Supplementary Table S5 Supplementary Table S5 lists the details for antibodies used in assay. This includes supplier, RRID, and working concentrations.

Supplementary Figure S1 Supplementary Figure S1 shows representative images of DLL3 IHC staining in tumor biopsies.

Supplementary Figure S2 Supplementary Figure S2 shows the CTC-iCHIP workflow and images of staining optimization using control cancer cell lines.

Supplementary Figure S3 Supplementary Figure S3 is a gallery of CTC images.

Supplementary Figure S4 Supplementary Figure S4 shows the size distribution of the CTCs based on their marker expression profiles.

Supplementary Figure S5 Supplementary Figure S5 shows single-cell transcriptional landscape of SCLC tumor biopsies and CNV for Cohort B.

Supplementary Figure S6 Supplementary Figure S6 shows the CNV of SCLC tumor biopsies for Cohort C.

Supplementary Figure S7 Supplementary Figure 7 shows CNV of SCLC tumor biopsies for patient-2.

Supplementary Figure S8 Supplementary Figure S8 shows fraction of DLL3 negative tumor cells based on single-cell RNA-seq “drop out” of rare transcripts.

Supplementary Figure S9 Supplementary Figure S9 shows that the fraction of DLL3-positive tumor cells does not significantly correlate with the distribution of SCLC molecular subtypes in primary tumors across cohorts.

Supplementary Figure S10 Supplementary Figure S10 shows the fraction of DLL3 positive cells for subtype SCLC-A and SCLC-N.

Supplementary Figure S11 Supplementary Figure S11 shows pseudobulk DLL3 and ASCL1 expression plotted by SCLC molecular subtype, including correlation analysis and subtype-stratified comparisons across tumors.

Supplementary Figure S12 Supplementary Figure S12 shows isoform characterization of DLL3 including protein structure, expression and isoform profiles for specific patients.

Supplementary Figure S13 Supplementary Figure S13 displays a gallery of CTFs in specific patient samples following the onset of tarlatamab therapy.

Supplementary Figure S14 Supplementary Figure S14 shows longitudinal data for patient 2 and the corresponding CTC and DLL3 fractions over time.

Supplementary Figure S15 Supplementary Figure S15 shows CD4+ T cells phenotypic profiling flow data for memory, activation and exhaustion.

Supplementary Figure S16 Supplementary Figure S16 shows CD4+ T cells phenotypic profiling for individual markers in control sample HD2.

Supplementary Figure S17 Supplementary Figure S17 shows graphs of CD4+ T cells phenotypic profiles for memory, activation and exhaustion.

Supplementary Figure S18 Supplementary Figure S18 shows bar graphs which represent the expression of SEZ6 and B7H3 across SCLC tumors in Cohort B.

Supplementary Figure S19 Supplementary Figure S19 shows Venn diagrams of CNV-confirmed cancer cells and their coexpression of DLL3, SEZ6, B7H3 for tumors in Cohort B.

Supplementary Figure S20 Supplementary Figure S20 shows shows bar graphs of expression of SEZ6 and B7H3 across SCLC tumors in Cohort C.

Supplementary Figure S21 Supplementary Figure S21 shows Venn diagrams of CNV-confirmed cancer cells and their coexpression of DLL3, SEZ6, B7H3 for tumors in Cohort C.

Supplementary Figure S22 Supplementary Figure S22 displays the coexpression of DLL3, SEZ6 and B7H3 for patient 36.

Supplementary Figure S23 Supplementary Figure S23 shows Venn diagrams of the coexpression of DLL3, EpCAM, and cytokeratin in Cohort B, Cohort C, and patient 37.

Supplementary Figure S24 Supplementary Figure S24 shows the distribution of DLL3 positive cells based on cell type for Cohort B and patient 37.

Supplementary References 1 Supplementary References includes cited works for the supplementary figures.

Supplementary Data S1 Supplementary Data S1 contains characteristic descriptions for Cohort B.

Supplementary Data S2 Supplementary Data S2 shows a summary of the coexpression analysis for Cohort B and Cohort C.

Supplementary Data S3 Supplementary Data S3 shows quality control metrics.

## Data Availability

Source data are provided with this article. RNA-seq raw FASTQ files have been deposited in the European Genome-phenome Archive with accession numbers EGAS50000001400 for scRNA-seq from tumors and EGAS50000001401 for scRNA-seq from CTCs. These data are available under restricted access to protect patient information due to IRB policy; access can be obtained upon request to the corresponding authors. All code for scRNA-seq analysis in this study is available at https://github.com/mlin2017/SCLC_response_to_Tarlatamab. The CTC images are available at https://osf.io/pn65c.
