# Supplementary material for: Circulating Tumor Cells Predict Response to the DLL3-Targeting Bispecific Antibody Tarlatamab
Source: Cancer Discov. 2026 Jan 14;16(5):911–30. doi: 10.1158/2159-8290.CD-25-1483 (PMC13067943; doi:10.1158/2159-8290.CD-25-1483)
Supplement: Supplementary Table S1 — shows patient demographics and clinical characteristics for Cohort A. [file cd-25-1483_supplementary_table_s1_suppst1.pdf]

Supplementary Table 1. Cohort A patient demographics and clinical characteristics.

**Characteristic**		**Total (n=32)**
**Median age at diagnosis (range)**		65 (49-81)
**Sex—no (%)**	Male	17 (53)
	Female	15 (47)
**Race—no (%)**	White	31 (97)
	Other/unavailable	1 (3)
**Ethnicity—no (%)**	Not Hispanic or Latino	31 (97)
	Hispanic or Latino	1 (3)
**Number of prior therapies—no (%)**	1	12 (38)
	2	9 (28)
	3+	11 (34)
**Prior systemic therapy regimens—no (%)**	Platinum doublet chemotherapy	32 (100)
	PD-L1 inhibitor	26 (81)
	Lurbinectedin	11 (34)
	Irinotecan	7 (22)
