# Supplementary material for: Circulating Tumor Cells Predict Response to the DLL3-Targeting Bispecific Antibody Tarlatamab
Source: Cancer Discov. 2026 Jan 14;16(5):911–30. doi: 10.1158/2159-8290.CD-25-1483 (PMC13067943; doi:10.1158/2159-8290.CD-25-1483)
Supplement: Supplementary Table S2 — shows the exclusion criteria for analysis cohort. [file cd-25-1483_supplementary_table_s2_suppst2.pdf]

**Supplementary Table S2.** Cohort A exclusion criteria.

| **Patient ID** | **Exclusion Criteria** |
|---|---|
| MGHSCLC_005 | Transformed SCLC |
| MGHSCLC_009 | Active brain metastases |
| MGHSCLC 010 | Inadequate follow-up imaging for response assessment |
| MGHSCLC_012 | Baseline staining technical failure |
| MGHSCLC_015 | No baseline draw |
| MGHSCLC_019 | Transformed SCLC |
| MGHSCLC_020 | Inadequate follow-up imaging for response assessment |
| MGHSCLC_022 | Transformed SCLC |
| MGHSCLC 027 | EP-NEC |
| MGHSCLC_029 | No CTCs at baseline |
| MGHSCLC_032 | EP-NEC |
| MGHSCLC_036 | Active brain metastases |

EP-NEC: Extrapulmonary Neuroendocrine Carcinoma
