# Supplementary material for: Circulating Tumor Cells Predict Response to the DLL3-Targeting Bispecific Antibody Tarlatamab
Source: Cancer Discov. 2026 Jan 14;16(5):911–30. doi: 10.1158/2159-8290.CD-25-1483 (PMC13067943; doi:10.1158/2159-8290.CD-25-1483)
Supplement: Supplementary Table S3 — shows pre-treatment assessments of CTCs for Cohort A. [file cd-25-1483_supplementary_table_s3_suppst3.pdf]

Supplementary Table S3. Cohort A baseline CTC assessments for eligible cases (N=20).

**Patient ID**	**Baseline CTC count in 20 mL Blood**	**Baseline CTC count per mL Blood**	**Baseline CTC DLL3 expression**
MGHSCLC_001	1416	70.8	100%
MGHSCLC_002	209	10.45	97%
MGHSCLC_004	9	0.45	33%
MGHSCLC_007	13	0.65	8%
MGHSCLC_008	4	0.2	75%
MGHSCLC_011	28	1.4	7%
MGHSCLC_016	46	2.3	13%
MGHSCLC_017	185	9.25	7%
MGHSCLC_018	97	4.85	3%
MGHSCLC_021	15	0.75	80%
MGHSCLC_024	5	0.25	0%
MGHSCLC_028	34	1.7	79%
MGHSCLC_030	2	0.1	50%
MGHSCLC_033	24	1.2	88%
MGHSCLC_034	5	0.25	20%
MGHSCLC_037	247	12.35	10%
MGHSCLC_038	272	13.6	81%
MGHSCLC_039	204	10.2	10%
MGHSCLC_040	87	4.35	100%
MGHSCLC_041	85	4.25	45%
