# Supplementary material for: Circulating Tumor Cells Predict Response to the DLL3-Targeting Bispecific Antibody Tarlatamab
Source: Cancer Discov. 2026 Jan 14;16(5):911–30. doi: 10.1158/2159-8290.CD-25-1483 (PMC13067943; doi:10.1158/2159-8290.CD-25-1483)
Supplement: Supplementary Table S4 — shows the DLL3 IHC data for Cohort A. [file cd-25-1483_supplementary_table_s4_suppst4.pdf]

Supplementary Table S4. Cohort A DLL3 immunohistochemistry (IHC) in tumor biopsies.

Patient ID	DLL3 percent staining	DLL3 H-score
MGHSCLC_001	95%	285
MGHSCLC_002	90%	270
MGHSCLC 004	100%, 100%	300, 300
MGHSCLC_007	90%	180
MGHSCLC_008	80%	160
MGHSCLC_011	0%	0
MGHSCLC_016	100%	300
MGHSCLC_017	95%	285
MGHSCLC 018	100%, 90%	200, 270
MGHSCLC_021	100%	300
MGHSCLC_024	100%	300
MGHSCLC_028	70%	140
MGHSCLC_030	70%	140
MGHSCLC_033*	ND	ND
MGHSCLC 034*	ND	ND
MGHSCLC 037	100%	300
MGHSCLC_038	100%	300
MGHSCLC_039	100%	300
MGHSCLC_040	90%	270
MGHSCLC_041	80%	240

The DLL3 percent staining represents the fraction of cells within the biopsy showing any detectable membrane or cytoplasmic signal.

The DLL3 H-score is a semi-quantitative metric that integrates both staining intensity and proportion of positive cells, resulting in a score range from 0 to 300. The H-score is determined by staining intensity (range = 0-3; 0: absent, 1: weak, 2: moderate; 3: strong) multiplied by the percentage of positive cells (0-100%).

ND: Not done. *No IHC completed due to tissue exhaustion.
