# Supplementary material for: Circulating Tumor Cells Predict Response to the DLL3-Targeting Bispecific Antibody Tarlatamab
Source: Cancer Discov. 2026 Jan 14;16(5):911–30. doi: 10.1158/2159-8290.CD-25-1483 (PMC13067943; doi:10.1158/2159-8290.CD-25-1483)
Supplement: Supplementary Table S5 — lists the details for antibodies used in assay. This includes supplier, RRID, and working concentrations. [file cd-25-1483_supplementary_table_s5_suppst5.pdf]

Supplementary Table S5. Working concentration of antibodies for immunofluorescent staining.

Component	RRID	Supplier	Catalog Number	Working Concentration (µg/mL)
**Primary**<br><br>Rabbit anti-DLL3	AB_2799809	CST	71804S	0.375
Mouse anti-epCAM AF488	AB_10692105	CST	5198S	5
Mouse anti-panCK AF488	AB_836889	CST	4523S	5
Mouse anti-CK19 AF488	AB_2539532	Invitrogen	MA5-18158	5
Mouse anti CD16 AF647	AB_492976	Biolegend	302020	5
Mouse anti-CD45 AF647	AB_389336	Biolegend	304056	5
Mouse anti CD66b AF647	AB_2563171	Biolegend	305110	5
**Secondary**<br><br>Goat anti-Rabbit AF594	AB_2756445	Abcam	ab150084	5
