# Supplementary material for: Circulating Tumor Cells Predict Response to the DLL3-Targeting Bispecific Antibody Tarlatamab
Source: Cancer Discov. 2026 Jan 14;16(5):911–30. doi: 10.1158/2159-8290.CD-25-1483 (PMC13067943; doi:10.1158/2159-8290.CD-25-1483)
Supplement: Supplementary Figure S1 — shows representative images of DLL3 IHC staining in tumor biopsies. [file cd-25-1483_supplementary_figure_s1_suppsf1.pdf]

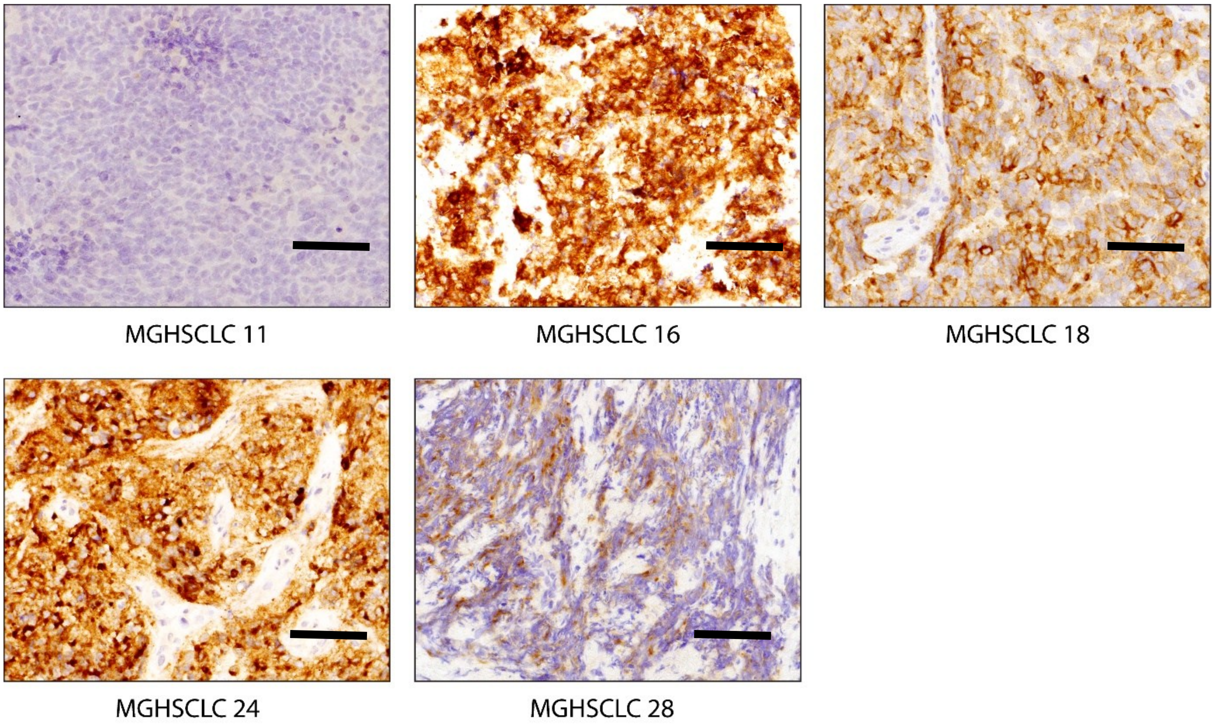

Supplementary Figure S1: Representative images of DLL3 IHC in tumor biopsies. IHC images showing DLL3 expression across five representative SCLC patient samples. Staining intensity and cellular localization demonstrate inter-patient heterogeneity, ranging from high, membrane-associated DLL3 expression to minimal or absent staining. Black scalebar is 100 µm.
