# Supplementary material for: Circulating Tumor Cells Predict Response to the DLL3-Targeting Bispecific Antibody Tarlatamab
Source: Cancer Discov. 2026 Jan 14;16(5):911–30. doi: 10.1158/2159-8290.CD-25-1483 (PMC13067943; doi:10.1158/2159-8290.CD-25-1483)
Supplement: Supplementary Figure S2 — shows the CTC-iCHIP workflow and images of staining optimization using control cancer cell lines. [file cd-25-1483_supplementary_figure_s2_suppsf2.pdf]

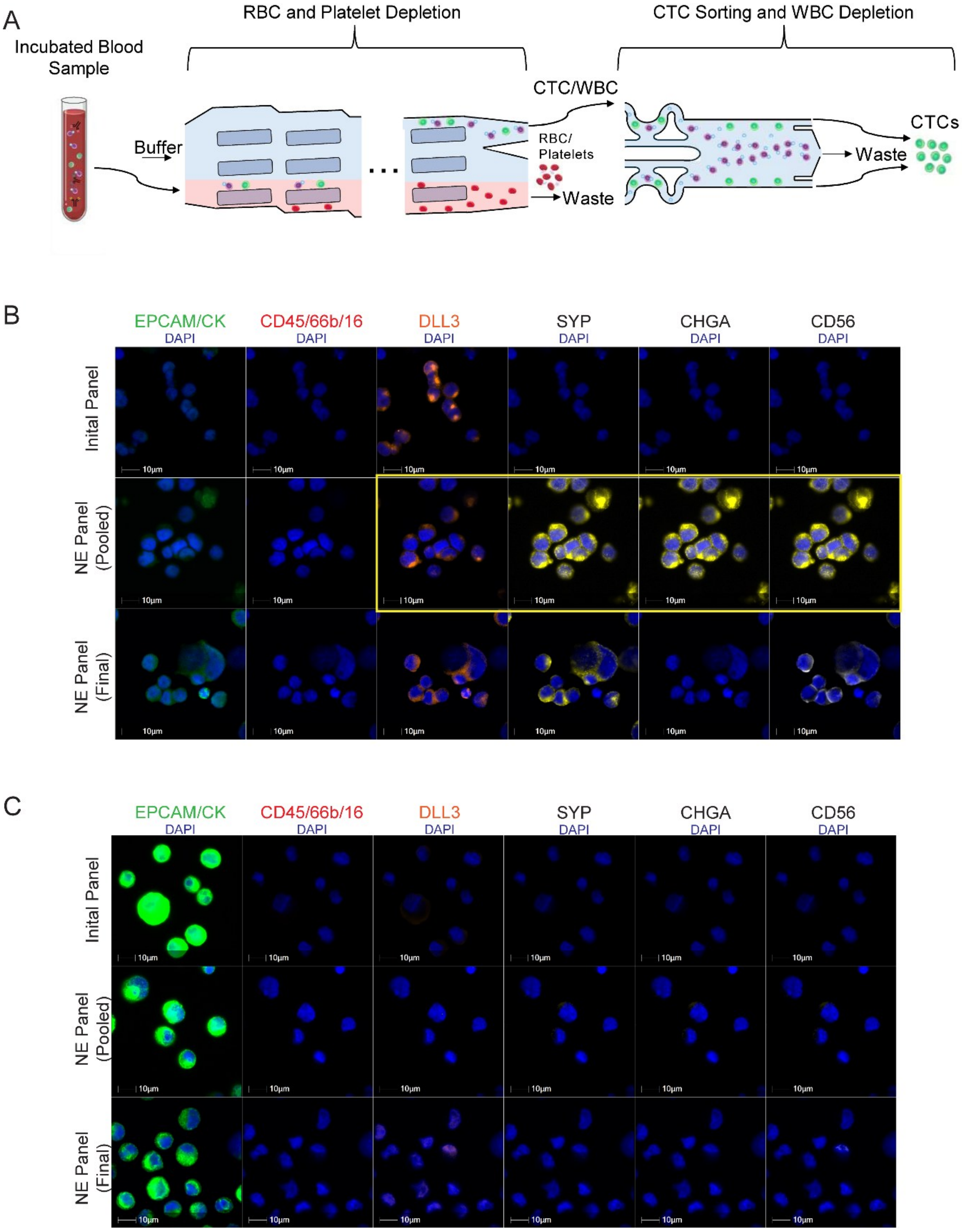

Supplementary Figure S2: CTC-iChip enrichment workflow and staining optimization using control cancer cell lines. (A) Schematic of the CTC-iChip workflow for rare cell enrichment. Whole blood is first processed through inertial focusing arrays to deplete smaller components such as red blood cells and platelets, based on size. The remaining nucleated cells, including CTCs and WBCs, are hydrodynamically aligned into a single flow stream and subsequently sorted

using magnetophoresis: WBCs prelabeled with magnetic beads are magnetically depleted (i.e., negative selection), yielding an enriched population of unlabeled CTCs. **(B-C)** Representative staining images of the neuroendocrine SCLC cell line NCI-H82 (B) and the epithelial (non-neuroendocrine) lung cancer cell line A549 (C). Both cell lines were stained using fluorescence-tagged antibody panels including DAPI (nuclear), EpCAM, pan-cytokeratin (CK) and CK19 (epithelial), CD45, CD66b, CD16 (hematopoietic), DLL3 (neuroendocrine), SYP (synaptophysin; neuroendocrine), CHGA (chromogranin A; neuroendocrine), and CD56 (neuroendocrine). The yellow box in (B) highlights positive staining for DLL3, SYP, CHGA, and CD56 in NCI-H82 cells, consistent with neuroendocrine marker expression. In contrast, A549 cells (C) lack expression of neuroendocrine markers but stain for epithelial epitopes. Notably, the neuroendocrine NCI-H82 cells show minimal expression of epithelial markers. Hematopoietic markers are negative (control).
