# Supplementary material for: Circulating Tumor Cells Predict Response to the DLL3-Targeting Bispecific Antibody Tarlatamab
Source: Cancer Discov. 2026 Jan 14;16(5):911–30. doi: 10.1158/2159-8290.CD-25-1483 (PMC13067943; doi:10.1158/2159-8290.CD-25-1483)
Supplement: Supplementary Figure S3 — is a gallery of CTC images. [file cd-25-1483_supplementary_figure_s3_suppsf3.pdf]

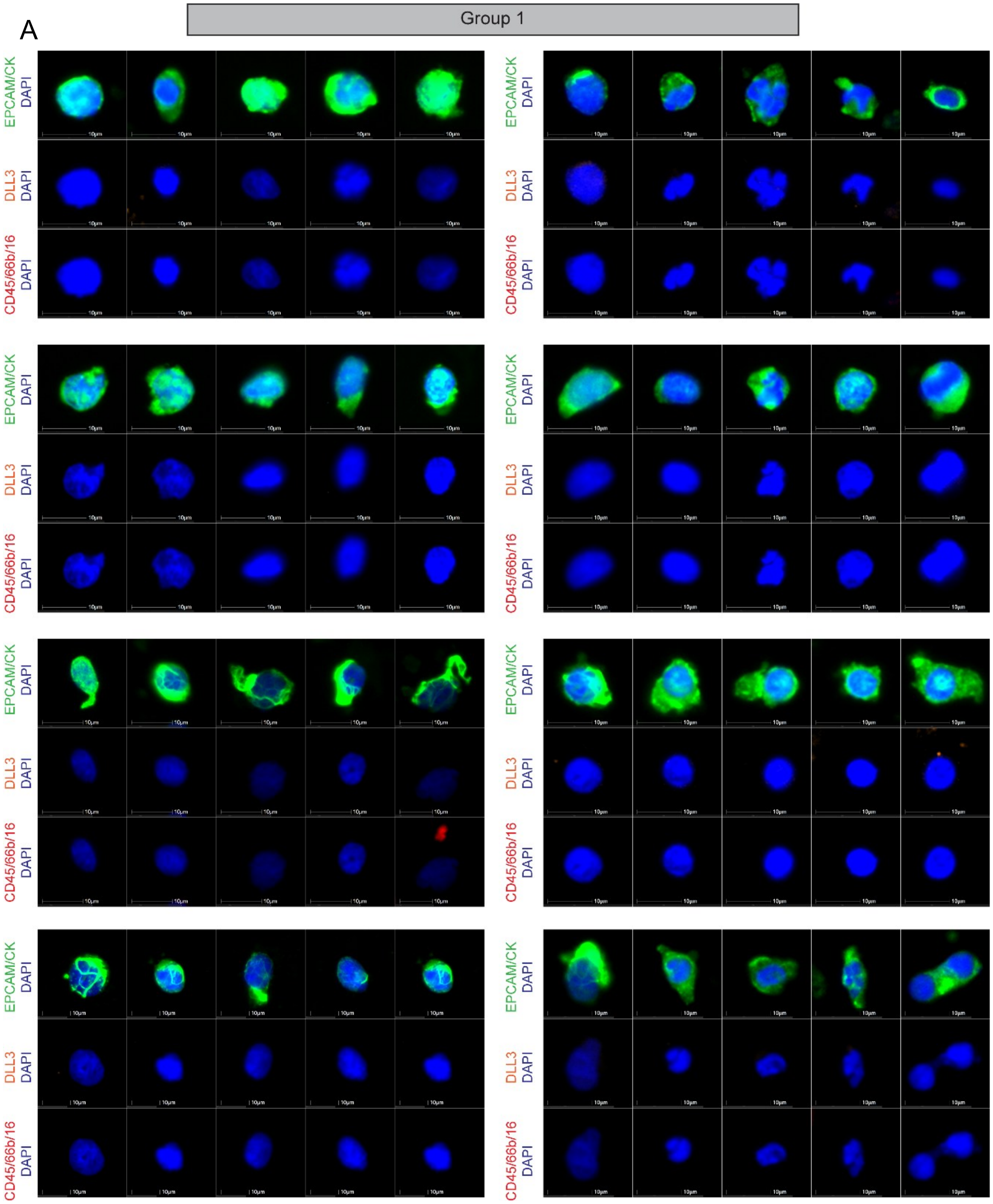

A
Group 1
EPCAM/CK DAPI
DLL3 DAPI
CD45/66b/16 DAPI
10µm

B

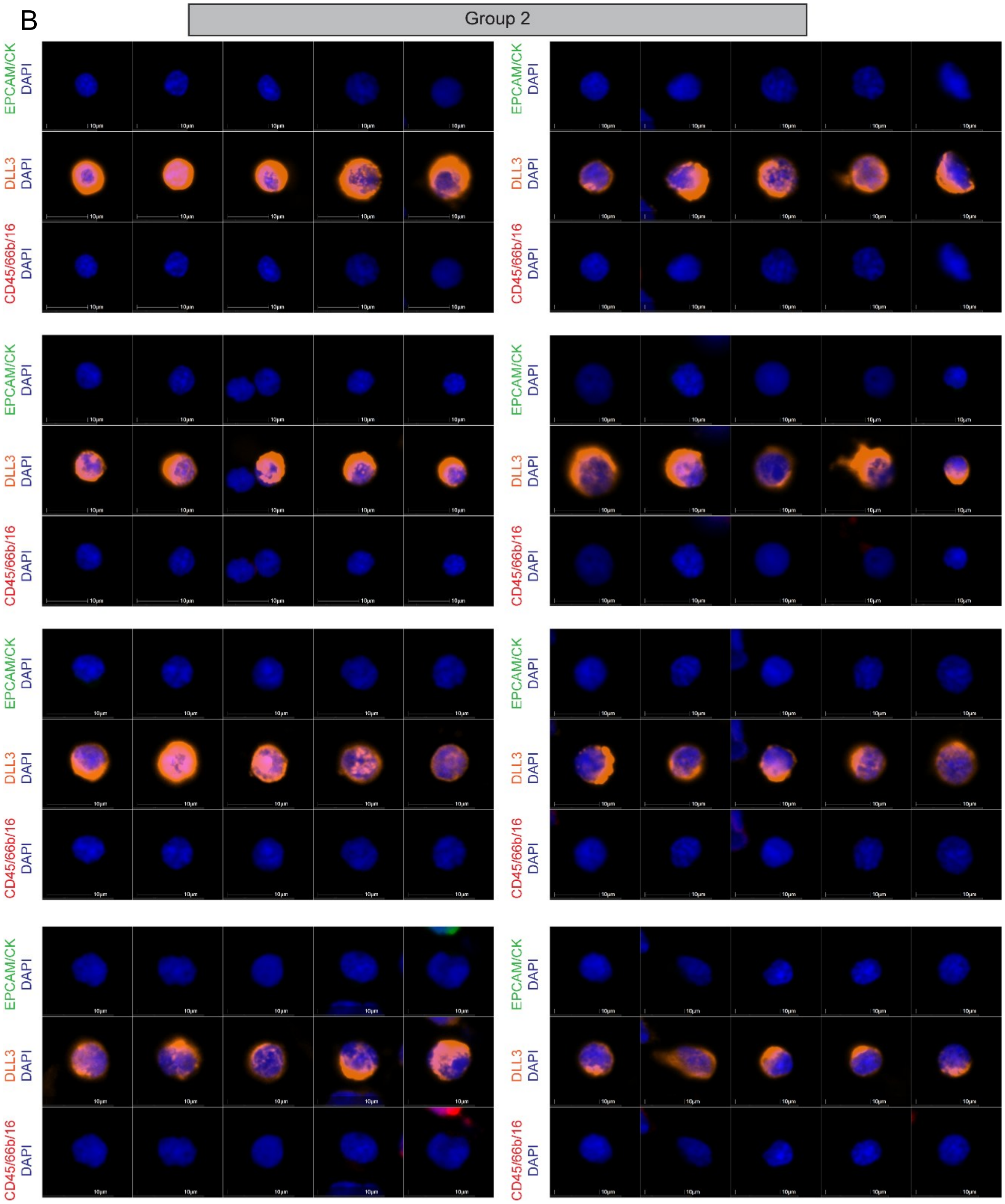

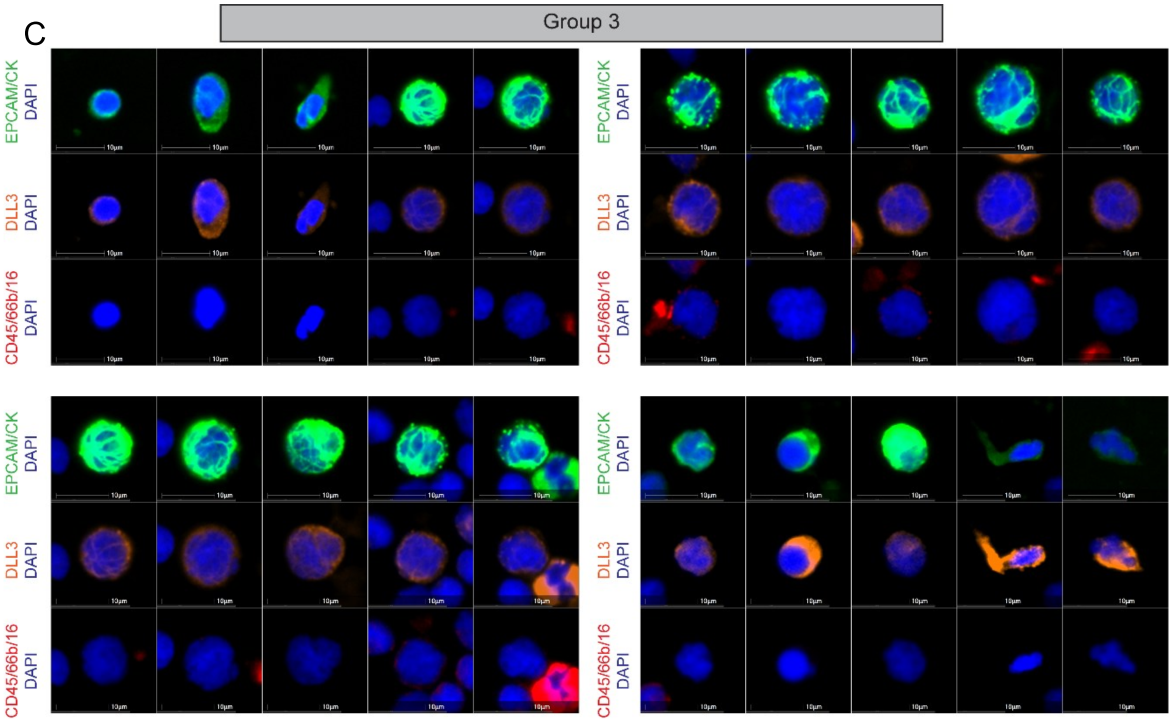

Supplementary Figure S3: Gallery of representative SCLC patient-derived CTCs (n = 100) showing distinct marker expression profiles. Cells were stained with DAPI (nuclear; blue), a cocktail of antibodies against EpCAM, pan-cytokeratin (CK), and CK19 (epithelial markers; green), an antibody against DLL3 (neuroendocrine marker; orange), and a cocktail of antibodies against hematopoietic cells (CD45, CD66b, and CD16; red, negative control). Images are grouped by phenotype: **(A)** Group 1 (40 CTCs from 8 patients), epithelial marker-positive only; **(B)** Group 2 (40 CTCs from 8 patients), DLL3-positive only; and **(C)** Group 3 (20 CTCs from 2 patients), dual-positive staining for epithelial markers and DLL3. The panel illustrates the quality of CTC staining and imaging, as well as the variable appearance of patient-derived CTCs expressing either epithelial markers, DLL3, or both.
