# Supplementary material for: Circulating Tumor Cells Predict Response to the DLL3-Targeting Bispecific Antibody Tarlatamab
Source: Cancer Discov. 2026 Jan 14;16(5):911–30. doi: 10.1158/2159-8290.CD-25-1483 (PMC13067943; doi:10.1158/2159-8290.CD-25-1483)
Supplement: Supplementary Figure S4 — shows the size distribution of the CTCs based on their marker expression profiles. [file cd-25-1483_supplementary_figure_s4_suppsf4.pdf]

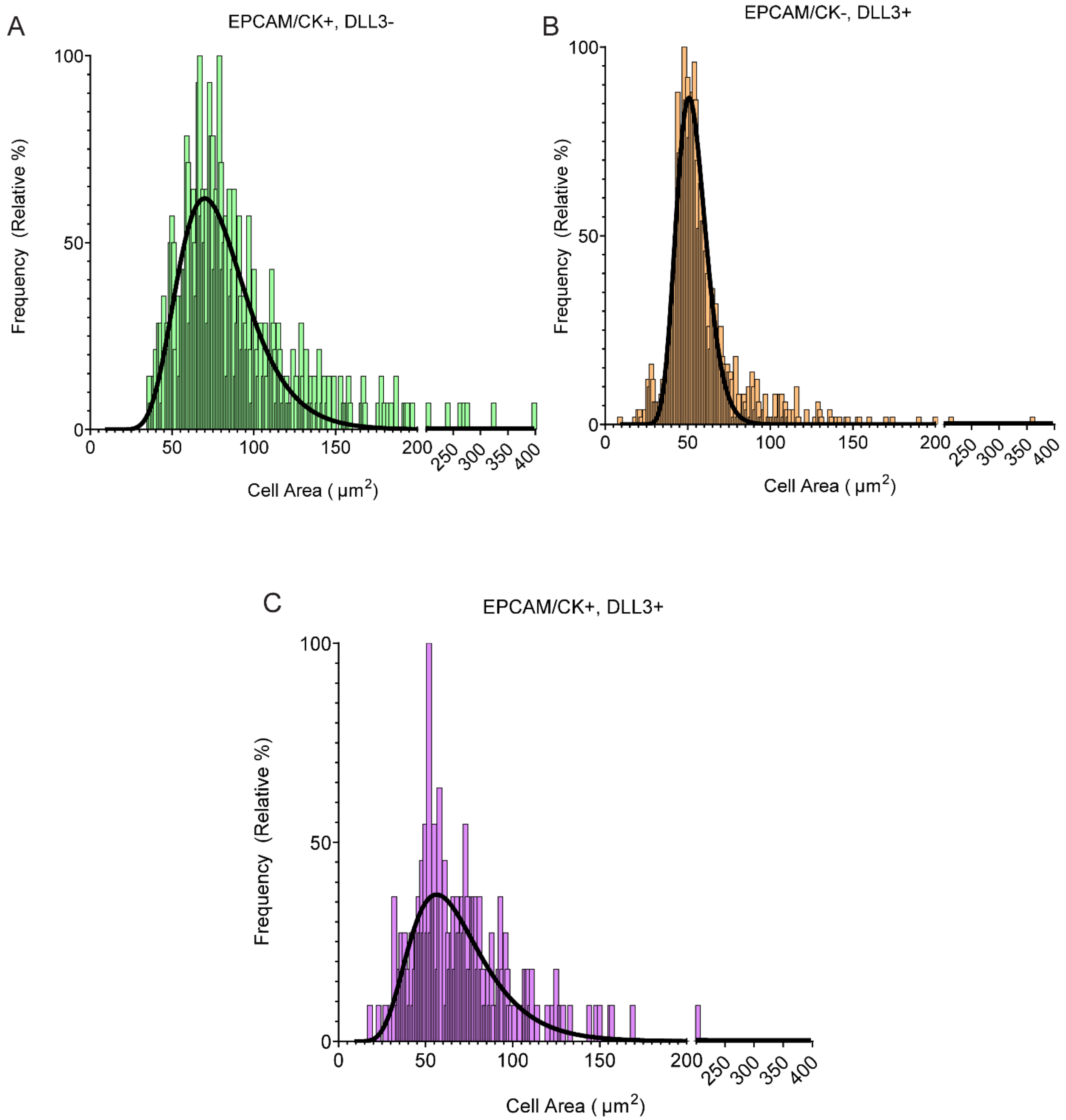

Supplementary Figure S4: Size distribution of CTCs based on marker expression profiles. Cell area is quantified for **(A)** individual CTCs expressing only epithelial markers (N = 932), **(B)** individual CTCs expressing only DLL3 (N = 1,727), and **(C)** individual CTCs co-expressing epithelial markers and DLL3 (N = 328). Size distributions are plotted as relative frequency (%) and fitted with a log-normal distribution using GraphPad Prism.
