# Supplementary material for: Circulating Tumor Cells Predict Response to the DLL3-Targeting Bispecific Antibody Tarlatamab
Source: Cancer Discov. 2026 Jan 14;16(5):911–30. doi: 10.1158/2159-8290.CD-25-1483 (PMC13067943; doi:10.1158/2159-8290.CD-25-1483)
Supplement: Supplementary Figure S5 — shows single-cell transcriptional landscape of SCLC tumor biopsies and CNV for Cohort B. [file cd-25-1483_supplementary_figure_s5_suppsf5.pdf]

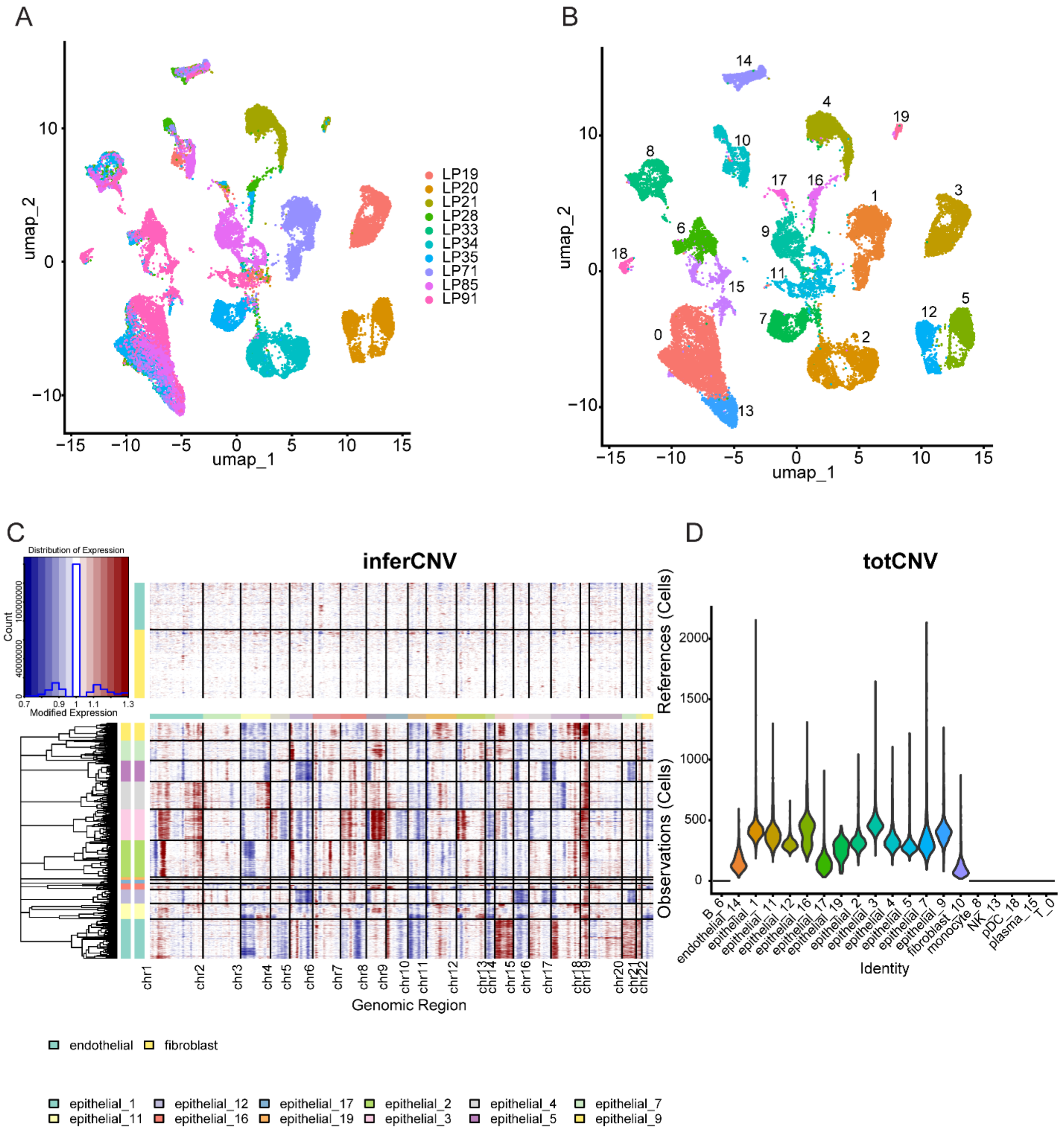

Supplementary Figure S5: Single-cell transcriptional landscape of SCLC tumor biopsies (Cohort B). (A-B) UMAP of transcriptional profiles from the 10 independent primary tumors annotated by either **(A)** patient identity (LP) or **(B)** transcriptional cluster number (1-19) to show both shared transcriptional patterns of non-malignant cells and tumor-specific clustering **(C)** Annotation of individual cancer cells within the 10 SCLC tumor cell populations, using RNA-based copy number variation (CNV) inference from epithelial cells expressing EpCAM transcripts**,** and using endothelial and fibroblast cells as “normal” diploid controls. **(D)** Violin plot of the CNV score for epithelial transcriptional clusters that are shown in panel (B). All epithelial transcriptional clusters have high CNV, with the exception of cluster 17, whose low CNV scores are compatible

with endothelial and fibroblast cells in the tumor microenvironment and is hence annotated as comprising non-malignant tumor-associated epithelial cells.
