# Supplementary material for: Circulating Tumor Cells Predict Response to the DLL3-Targeting Bispecific Antibody Tarlatamab
Source: Cancer Discov. 2026 Jan 14;16(5):911–30. doi: 10.1158/2159-8290.CD-25-1483 (PMC13067943; doi:10.1158/2159-8290.CD-25-1483)
Supplement: Supplementary Figure S6 — shows the CNV of SCLC tumor biopsies for Cohort C. [file cd-25-1483_supplementary_figure_s6_suppsf6.pdf]

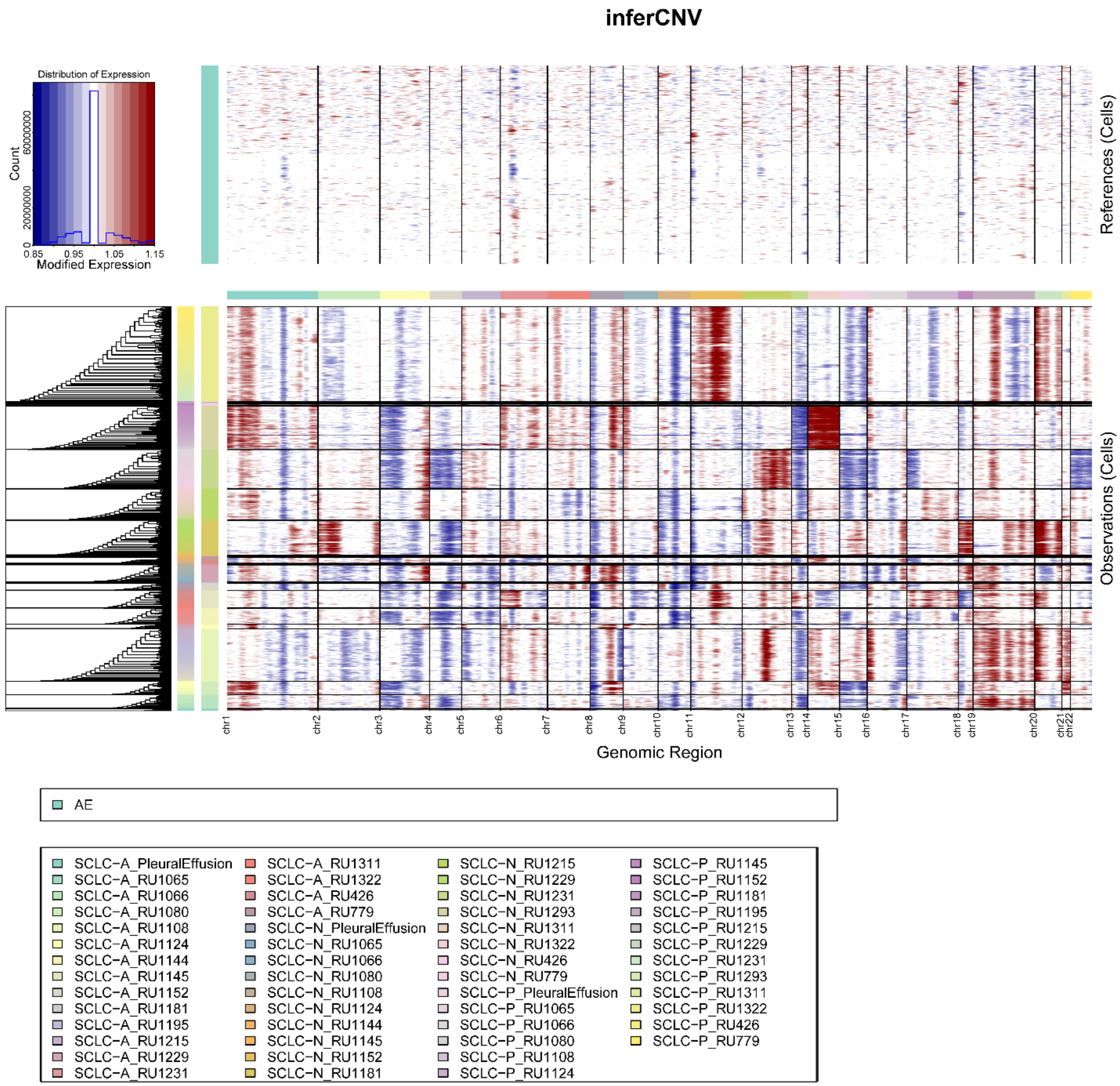

Supplementary Figure S6: Copy number inference of SCLC tumor biopsies (Cohort C). CNV profile of tumor cells in Cohort C was inferred using Alveolar Epithelial (AE) cells as reference, and tumor cells were grouped by SCLC subtype and patient identity. The inferred CNV profile identifies previously reported SCLC copy number patterns such as gains of chromosome 1p, 3q, 8q, 14, 18 and losses of 3p, 13q, 15q, 16q, and illustrates the presence of substantial intratumor heterogeneity.
