# Supplementary material for: Circulating Tumor Cells Predict Response to the DLL3-Targeting Bispecific Antibody Tarlatamab
Source: Cancer Discov. 2026 Jan 14;16(5):911–30. doi: 10.1158/2159-8290.CD-25-1483 (PMC13067943; doi:10.1158/2159-8290.CD-25-1483)
Supplement: Supplementary Figure S7 — Supplementary Figure 7 shows CNV of SCLC tumor biopsies for patient-2. [file cd-25-1483_supplementary_figure_s7_suppsf7.pdf]

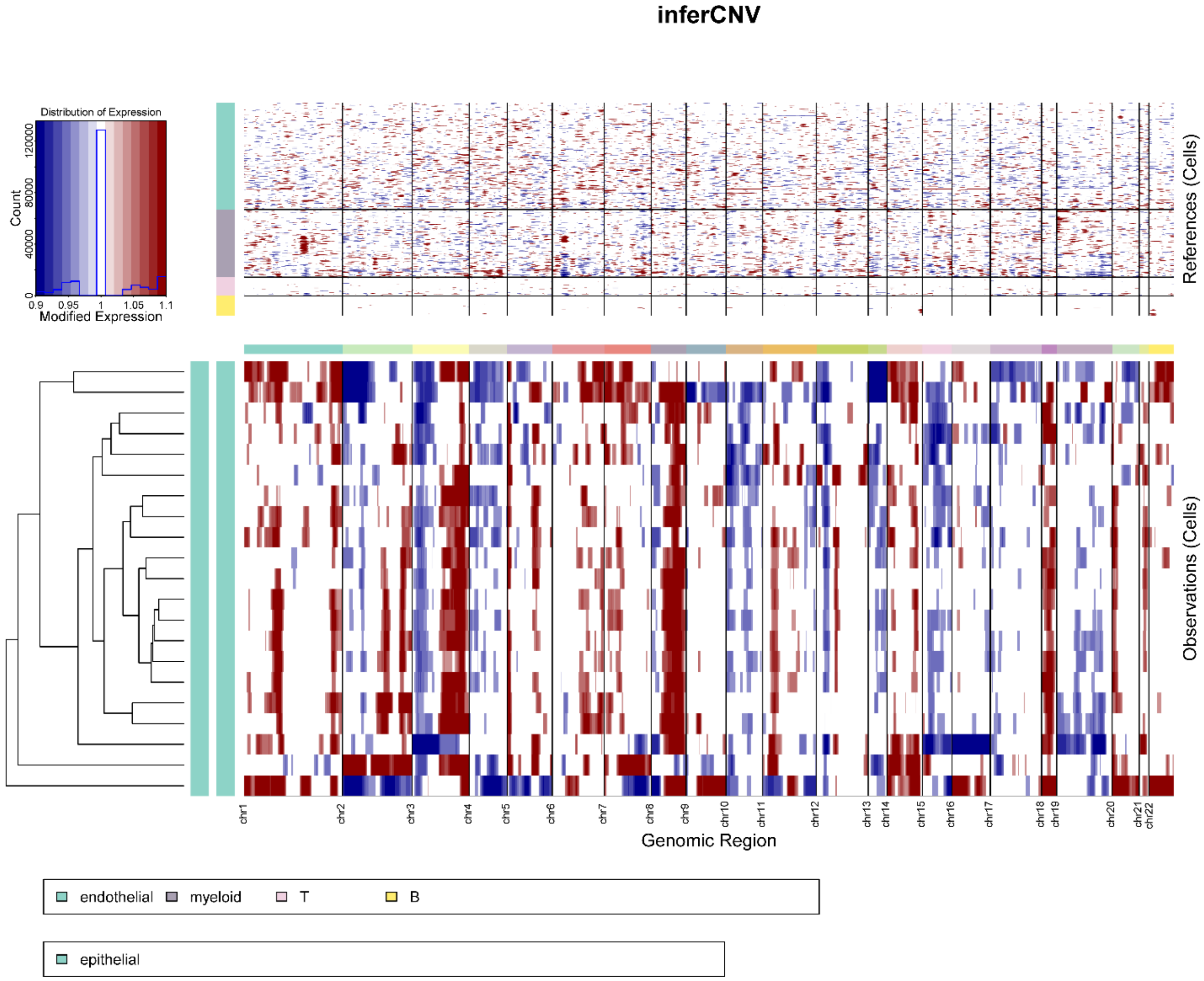

Supplementary Figure S7: Copy number inference of SCLC tumor biopsies (Patient-2). Tumor cells in patient-2 demonstrate SCLC associated copy number changes including gains of chromosome 3q, 8q, and 18, and losses of 3p, 4 and 13q. The CNV profile was inferred using stromal and immune cells as "normal" reference.
