# Supplementary material for: Circulating Tumor Cells Predict Response to the DLL3-Targeting Bispecific Antibody Tarlatamab
Source: Cancer Discov. 2026 Jan 14;16(5):911–30. doi: 10.1158/2159-8290.CD-25-1483 (PMC13067943; doi:10.1158/2159-8290.CD-25-1483)
Supplement: Supplementary Figure S8 — shows fraction of DLL3 negative tumor cells based on single-cell RNA-seq “drop out” of rare transcripts. [file cd-25-1483_supplementary_figure_s8_suppsf8.pdf]

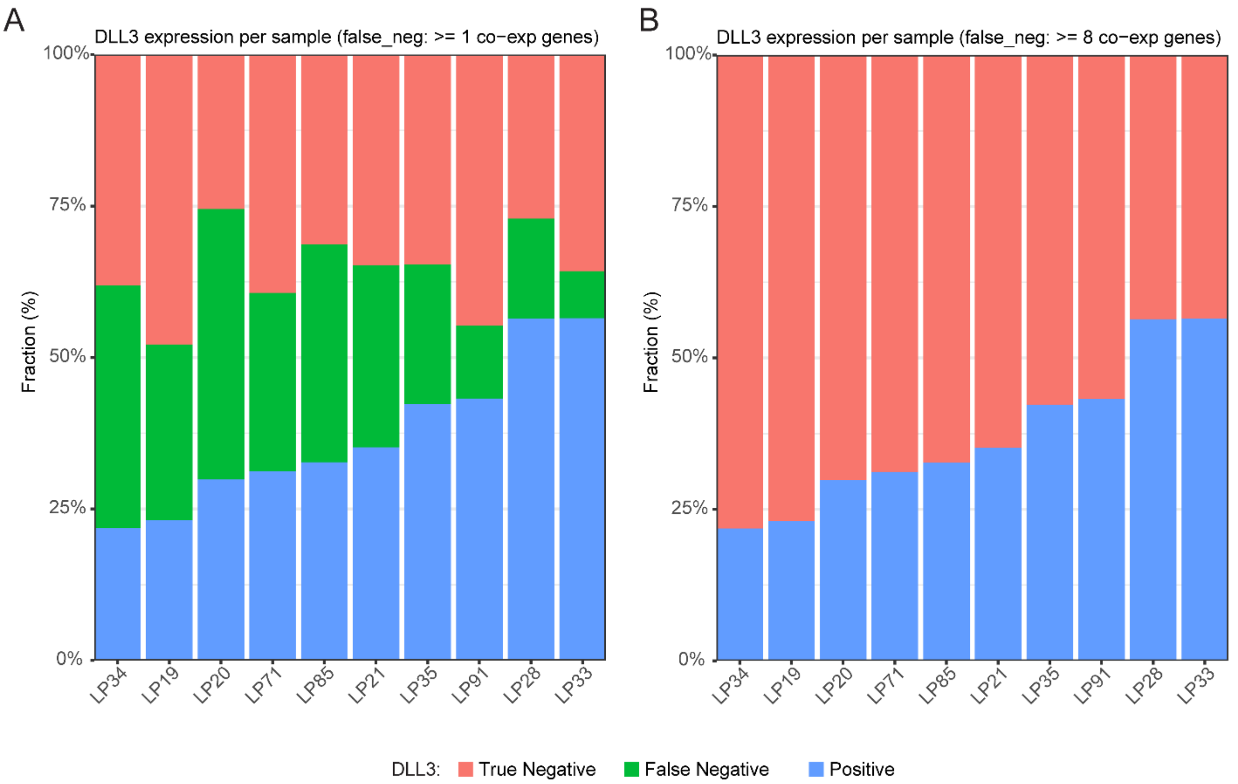


**Supplementary Figure S8: Calculation of range of DLL3 negative tumor cells attributed to single cell RNA-seq “drop out” of rare transcripts. (A)** Estimation of “false negative” DLL3 SCLC tumor cells attributable to “dropout” scRNA-seq, based on measurements of co-regulated Notch pathway genes (NOTCH1, NOTCH2, NOTCH3, NOTCH4, HES1, HEY1, JAG1, JAG2) using a network-based approach(1,2). Cells were classified based on DLL3 expression and co-expression of Notch pathway genes to estimate the proportion of true and false DLL3-negative cells. Cells with detectable DLL3 expression were classified as true positive (blue). Among DLL3 negative cells, those expressing only one other Notch pathway coregulated gene (left panel) or all eight other Notch pathway genes (right panel) were classified as potential false negatives (green), reflecting the possibility of scRNA-seq transcript dropouts. Cells lacking DLL3 and lacking either one of eight co-regulated Notch pathway genes were classified as likely true DLL3-negatives (red). **(B)** Independent of the stringency applied for calculation and correction of false negative reads, intratumoral DLL3 heterogeneity (i.e., coexistence of DLL3-positive and DLL3-negative SCLC tumor cells) was observed for every case in cohort B.
