# Supplementary material for: Circulating Tumor Cells Predict Response to the DLL3-Targeting Bispecific Antibody Tarlatamab
Source: Cancer Discov. 2026 Jan 14;16(5):911–30. doi: 10.1158/2159-8290.CD-25-1483 (PMC13067943; doi:10.1158/2159-8290.CD-25-1483)
Supplement: Supplementary Figure S9 — shows that the fraction of DLL3-positive tumor cells does not significantly correlate with the distribution of SCLC molecular subtypes in primary tumors across cohorts. [file cd-25-1483_supplementary_figure_s9_suppsf9.pdf]

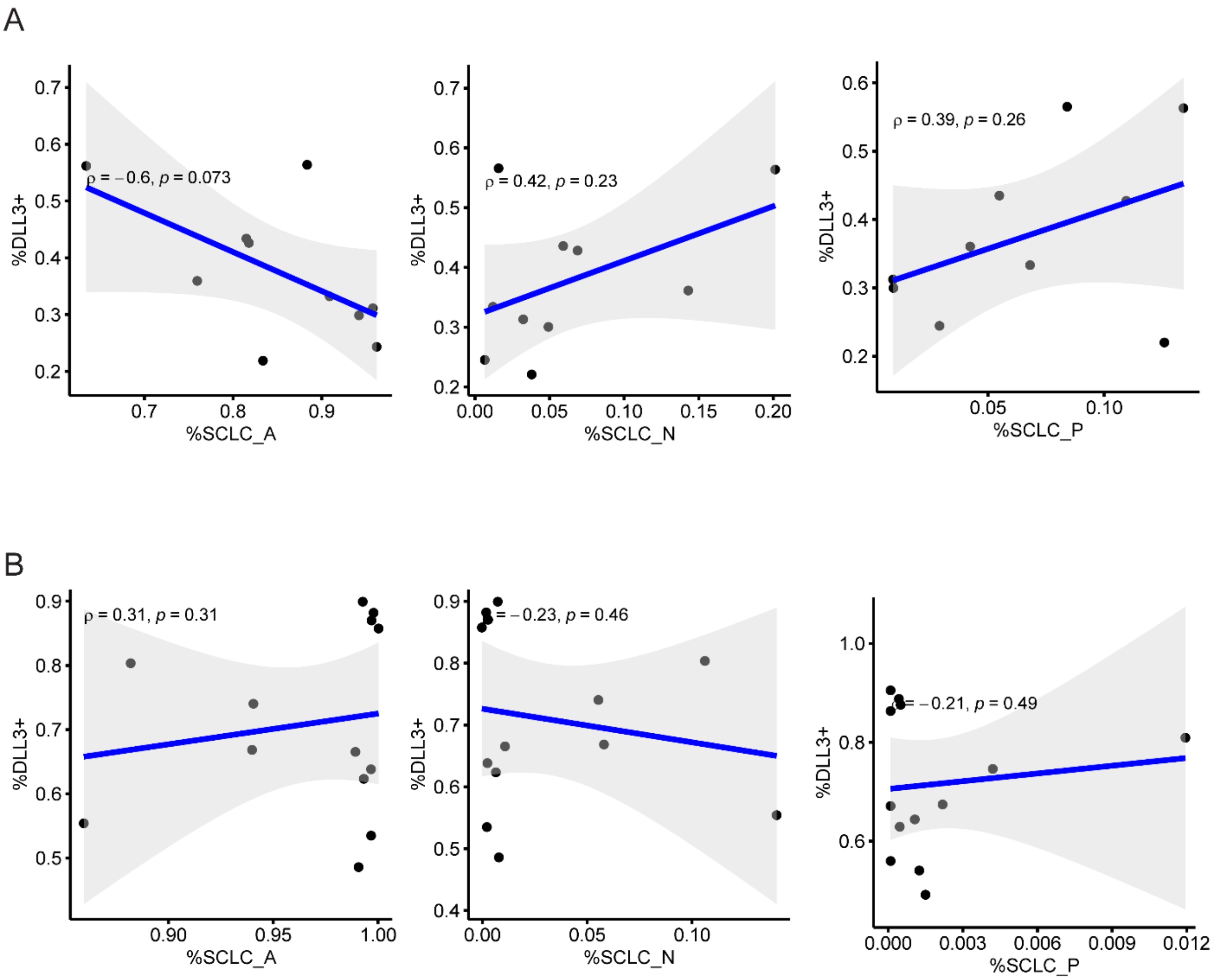

Supplementary Figure S9: Absence of significant correlation between the fraction of tumor cells with DLL3 expression and SCLC molecular subtypes in primary tumors. (A-B) Correlation plotted for the fraction of *DLL3*+ tumor cells within an individual tumor and the proportion of cells within that tumor bearing a specific molecular subtype (SCLC-A, SCLC-N, SCLC-P) for (A) Cohort B and (B) Cohort C with SCLC-A patients (3). Spearman correlation coefficient ρ and p values are shown, all with $p > 0.05$.
