# Supplementary material for: Circulating Tumor Cells Predict Response to the DLL3-Targeting Bispecific Antibody Tarlatamab
Source: Cancer Discov. 2026 Jan 14;16(5):911–30. doi: 10.1158/2159-8290.CD-25-1483 (PMC13067943; doi:10.1158/2159-8290.CD-25-1483)
Supplement: Supplementary Figure S10 — shows the fraction of DLL3 positive cells for subtype SCLC-A and SCLC-N. [file cd-25-1483_supplementary_figure_s10_suppsf10.pdf]

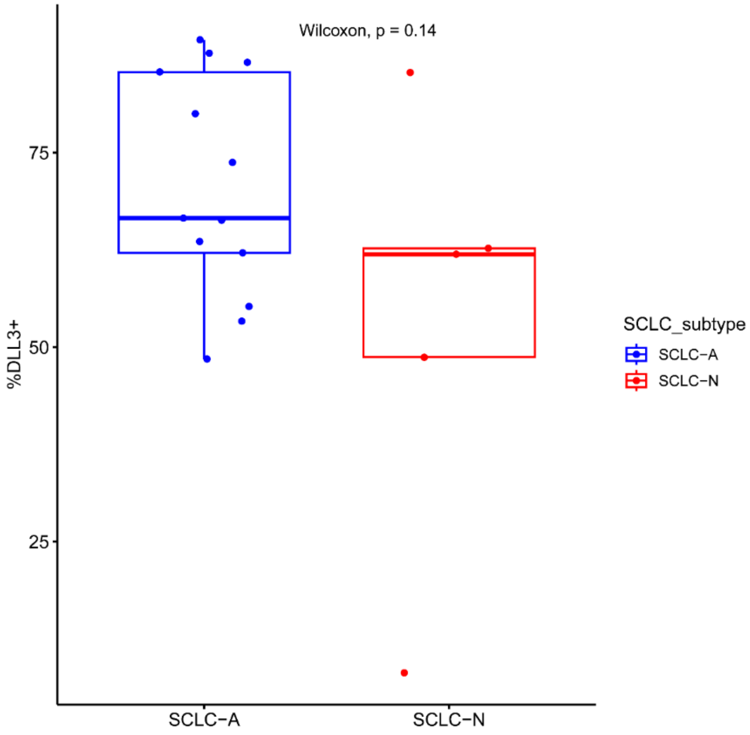

Supplementary Figure S10: Fraction of *DLL3*-positive cells in the two predominant SCLC molecular subtypes. Fraction of *DLL3*-positive cells within SCLC-A versus SCLC-N predominant tumors from Cohort C (Wilcoxon p = 0.14) (1).
