# Supplementary material for: Circulating Tumor Cells Predict Response to the DLL3-Targeting Bispecific Antibody Tarlatamab
Source: Cancer Discov. 2026 Jan 14;16(5):911–30. doi: 10.1158/2159-8290.CD-25-1483 (PMC13067943; doi:10.1158/2159-8290.CD-25-1483)
Supplement: Supplementary Figure S11 — shows pseudobulk DLL3 and ASCL1 expression plotted by SCLC molecular subtype, including correlation analysis and subtype-stratified comparisons across tumors. [file cd-25-1483_supplementary_figure_s11_suppsf11.pdf]

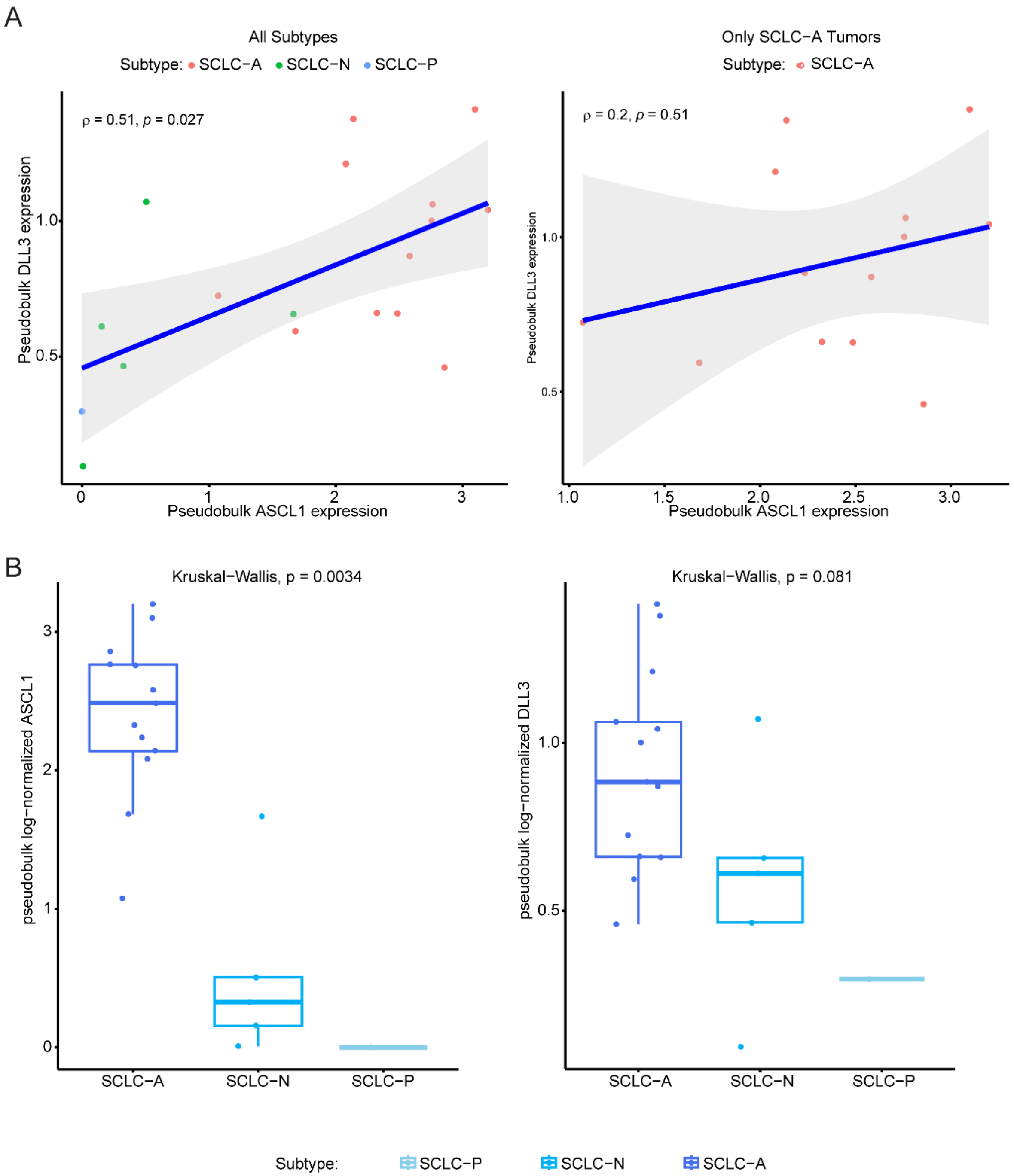

Supplementary Figure S11: Pseudobulk DLL3 and ASCL1 expression levels based on SCLC subtype. (A) Dot plot and correlation analysis of pseudobulk ASCL1 and DLL3 expression across molecular subtypes within cohort C. Consistent with prior reports, ASCL1 and DLL3 show a positive correlation when multiple SCLC subtypes (A, N, P) are considered together. However, this correlation disappears when analysis is restricted to SCLC-A tumors alone, indicating that the apparent association is driven by inter-subtype differences rather than coordinated regulation within a single subtype. **(B)** Box plot of pseudobulk DLL3 expression by molecular subtype (SCLC-A, SCLC-N, SCLC-P). In line with panel A, SCLC-A tumors (n = 13) show higher DLL3

expression than SCLC-N (n = 5) and SCLC-P (n = 1). For this analysis, single-cell RNA-seq data were analyzed at the pseudobulk level to generate a quantitative measurement of total DLL3 expression level for each tumor. P values for comparison of SCLC-A vs other molecular subtypes are 0.0034 (ASCL1) and 0.081 (DLL3).
