# Supplementary material for: Circulating Tumor Cells Predict Response to the DLL3-Targeting Bispecific Antibody Tarlatamab
Source: Cancer Discov. 2026 Jan 14;16(5):911–30. doi: 10.1158/2159-8290.CD-25-1483 (PMC13067943; doi:10.1158/2159-8290.CD-25-1483)
Supplement: Supplementary Figure S12 — shows isoform characterization of DLL3 including protein structure, expression and isoform profiles for specific patients. [file cd-25-1483_supplementary_figure_s12_suppsf12.pdf]

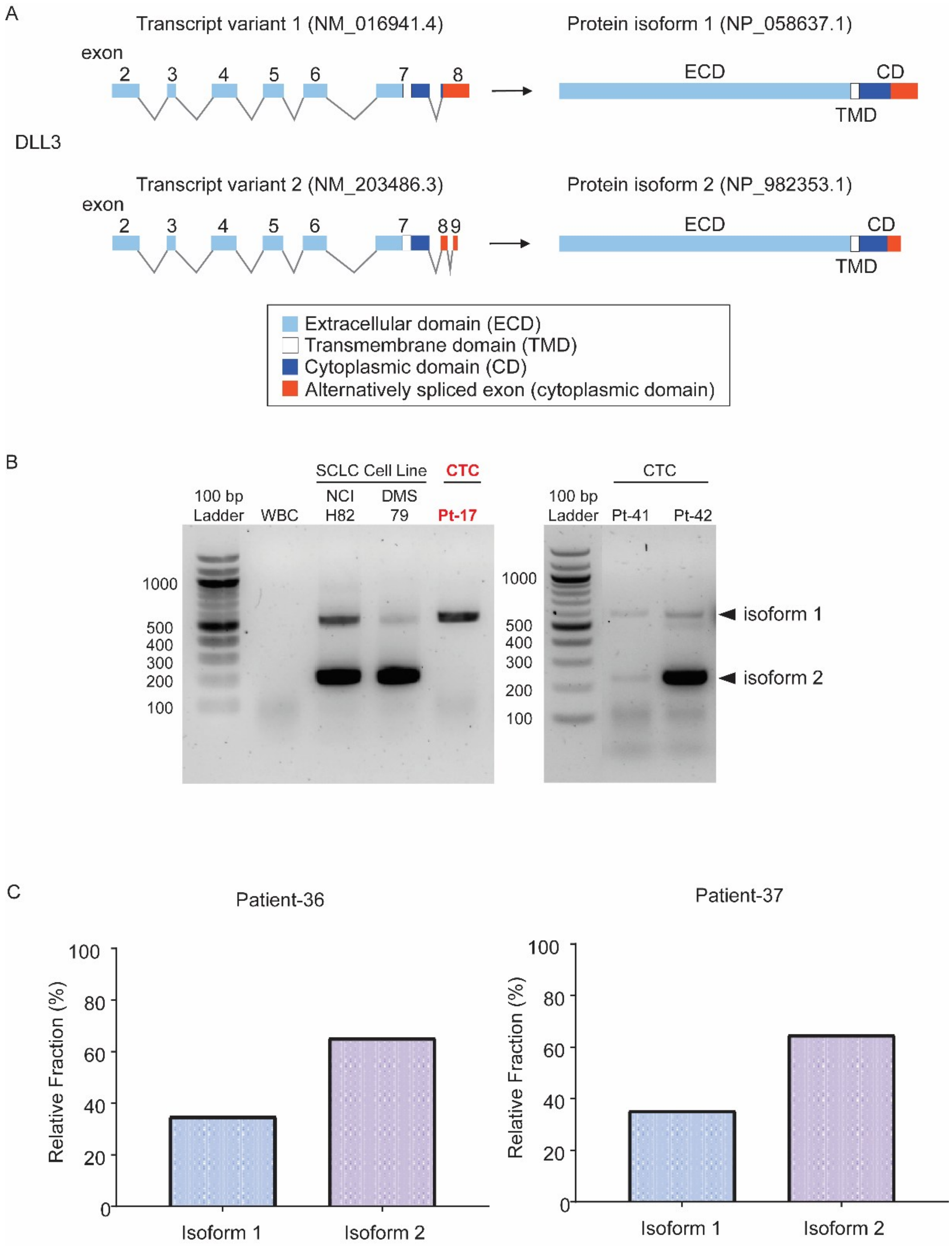

Supplementary Figure S12: DLL3 isoform characterization in SCLC: protein structure, expression, and patient-specific isoform profiles. (A) Schematic representation of DLL3 genomic structure showing the extracellular domain (ECD) recognized by tarlatamab, the transmembrane domain (TMD), and the cytoplasmic domain (CD). An intra-exon 8 alternative splicing event, within the cytoplasmic domain, generates two isoforms (1 and 2). The peptide sequence used to generate the diagnostic antibody used in this study (Cell Signaling Technology #71804) is located precisely at the 5’ flank of the splice junction, and it is preserved

in both isoforms; however, protein folding in the larger isoform 1 may influence its accessibility in immunofluorescence staining. **(B)** RT-PCR gel representing DLL3 isoform 1 and isoform 2 in two established SCLC cell lines (NCI-H82, DMS79), with predominance of the shorter isoform 2, and in two control patient-derived CTCs. CTCs from patient-42, which stain strongly for DLL3 (67% of CTCs), show a predominance of isoform 2; CTCs from patient-41, with low DLL3 staining (24% of CTCs), have lower total RNA signal for both isoforms, which are comparable in abundance. Patient-17 is remarkable for having had a sustained clinical response following treatment with tarlatamab, which recognizes the extracellular domain of DLL3, but also having a minimally detectable DLL3 signal on CTCs (7% of CTCs) using the diagnostic antibody recognizing the exon 8 splice flanking epitope within the cytoplasmic domain. RT-PCR in this unique case shows a complete absence of the shorter isoform 2 of DLL3. cDNA nucleotide sequencing of the entire cytoplasmic domain of DLL3, including exon 8 splice junctions, adjoining sequences and the spliced-out sequence revealed no mutations (not shown), implicating more distal or trans factors in this aberrant mRNA splicing. **(C)** Bar graph showing the relative proportion of DLL3 reads unique to isoform 1 versus isoform 2 within CTCs from patient-36 and patient-37. These cases had been analyzed by 10X single-cell RNA seq, making these precise determinations possible. Among all DLL3 mRNA reads in these two cases, a total of 2,268 allowed discrimination between the isoforms.
