# Supplementary material for: Circulating Tumor Cells Predict Response to the DLL3-Targeting Bispecific Antibody Tarlatamab
Source: Cancer Discov. 2026 Jan 14;16(5):911–30. doi: 10.1158/2159-8290.CD-25-1483 (PMC13067943; doi:10.1158/2159-8290.CD-25-1483)
Supplement: Supplementary Figure S13 — displays a gallery of CTFs in specific patient samples following the onset of tarlatamab therapy. [file cd-25-1483_supplementary_figure_s13_suppsf13.pdf]

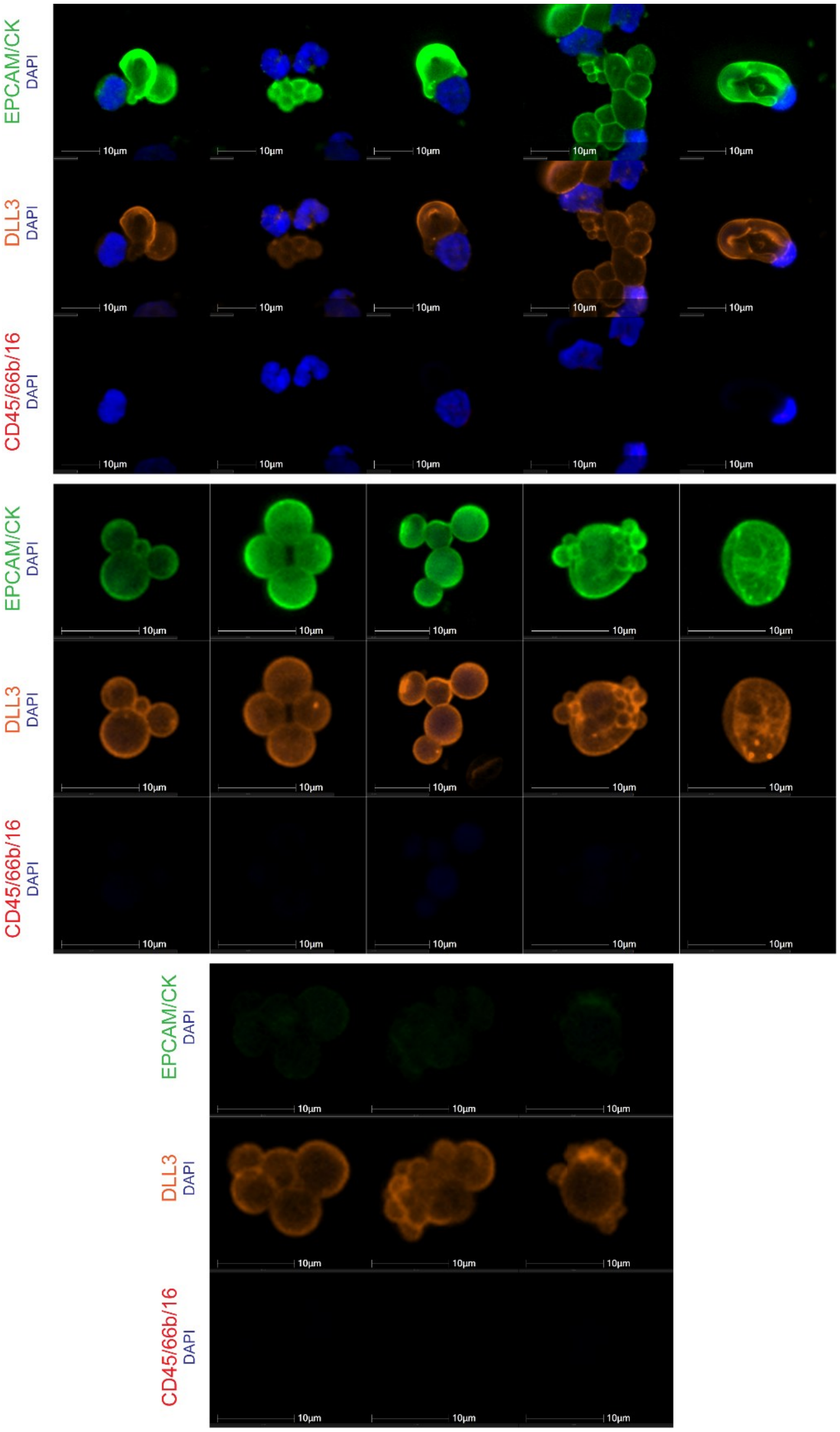

Supplementary Figure S13: Gallery of images of circulating tumor fragments (CTFs) detected in patient samples following initiation of tarlatamab therapy. CTFs were stained for epithelial markers (EPCAM, pan-CK, CK19, green), DLL3 (orange), nuclear stain (DAPI, purple), and hematopoietic markers (CD45, CD66b, CD16, red). Single CTFs and CTF clusters extruding their nucleus or nuclear fragments are shown in the upper panel, while anucleated CTFs and CTF

clusters are shown in the lower panels. The bottom panel shows anucleated CTFs expressing DLL3 only.
