# Supplementary material for: Circulating Tumor Cells Predict Response to the DLL3-Targeting Bispecific Antibody Tarlatamab
Source: Cancer Discov. 2026 Jan 14;16(5):911–30. doi: 10.1158/2159-8290.CD-25-1483 (PMC13067943; doi:10.1158/2159-8290.CD-25-1483)
Supplement: Supplementary Figure S14 — shows longitudinal data for patient 2 and the corresponding CTC and DLL3 fractions over time. [file cd-25-1483_supplementary_figure_s14_suppsf14.pdf]

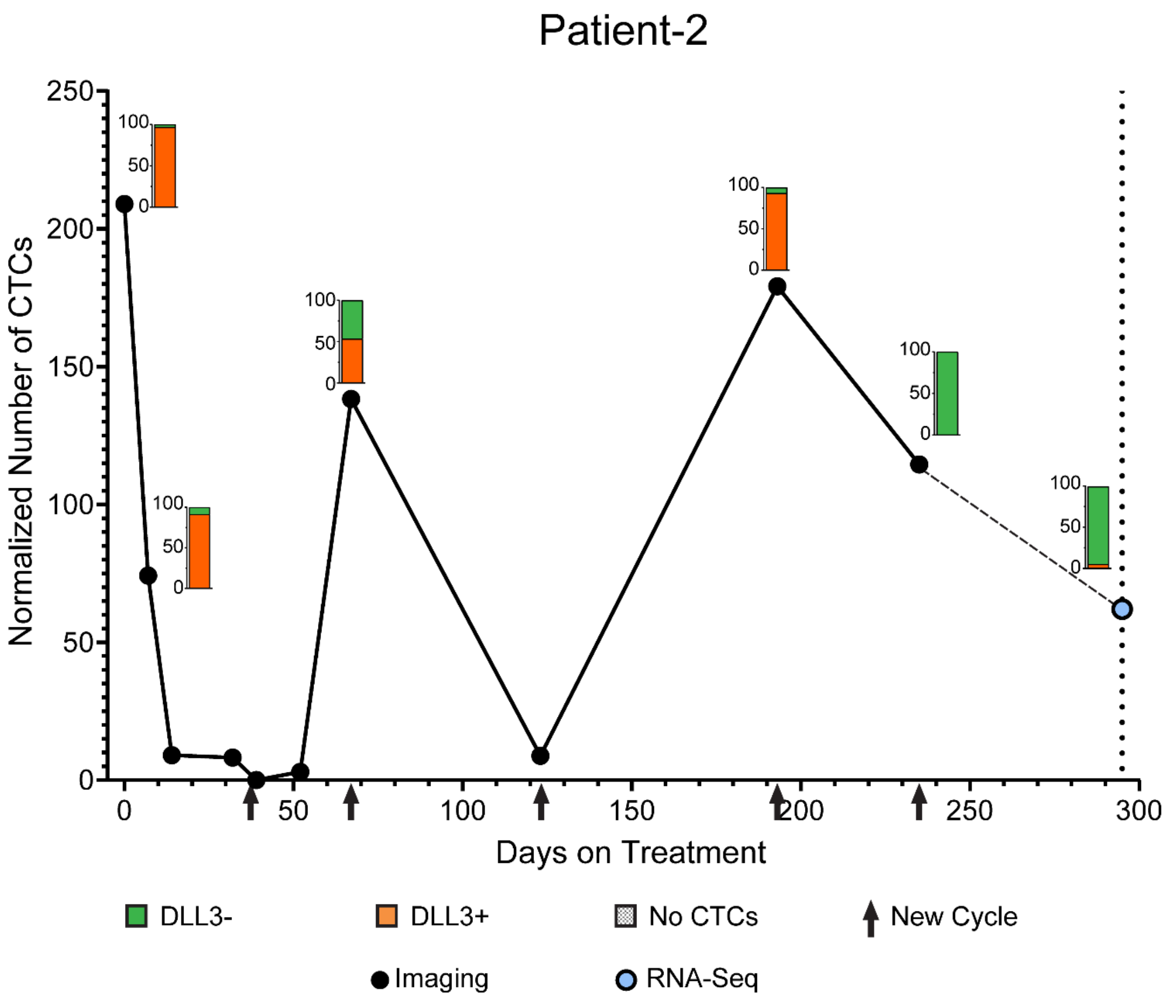

Supplementary Figure S14: Loss of DLL3 expression on CTCs at disease progression on tarlatamab (Patient-2). Longitudinal monitoring of patient-2, with highly abundant DLL3-positive CTCs at pretreatment baseline, a rapid response and decline in CTC numbers, followed by intermittent rebounds in CTC counts during the course of tarlatamab therapy (arrows). CTC counts are based on immunofluorescence imaging (black dots) and RNA-Sequencing (a light blue dot). Note that the dotted line indicates the clinically determined disease progression timepoint. At the closest timepoint to clinically determined disease progression, CTCs show a complete absence of the DLL3 epitope.
