# Supplementary material for: Circulating Tumor Cells Predict Response to the DLL3-Targeting Bispecific Antibody Tarlatamab
Source: Cancer Discov. 2026 Jan 14;16(5):911–30. doi: 10.1158/2159-8290.CD-25-1483 (PMC13067943; doi:10.1158/2159-8290.CD-25-1483)
Supplement: Supplementary Figure S15 — shows CD4+ T cells phenotypic profiling flow data for memory, activation and exhaustion. [file cd-25-1483_supplementary_figure_s15_suppsf15.pdf]

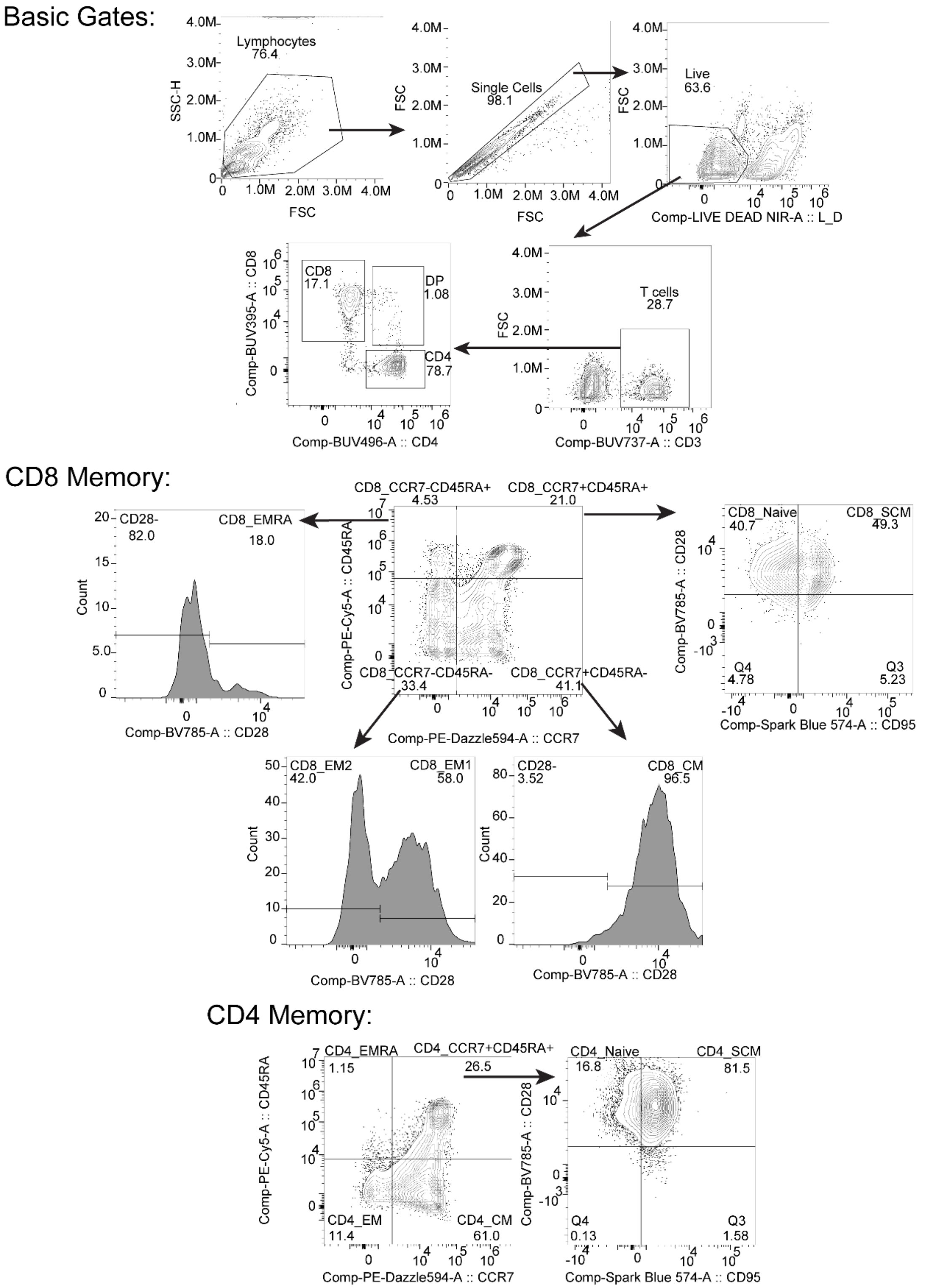

Supplementary Figure S15: Phenotypic profiling of memory, activation, and exhaustion markers in CD4⁺ T cells. Flow cytometry gating strategy is shown for the control sample HD2.
