# Supplementary material for: Circulating Tumor Cells Predict Response to the DLL3-Targeting Bispecific Antibody Tarlatamab
Source: Cancer Discov. 2026 Jan 14;16(5):911–30. doi: 10.1158/2159-8290.CD-25-1483 (PMC13067943; doi:10.1158/2159-8290.CD-25-1483)
Supplement: Supplementary Figure S16 — shows CD4+ T cells phenotypic profiling for individual markers in control sample HD2. [file cd-25-1483_supplementary_figure_s16_suppsf16.pdf]

Individual Markers:

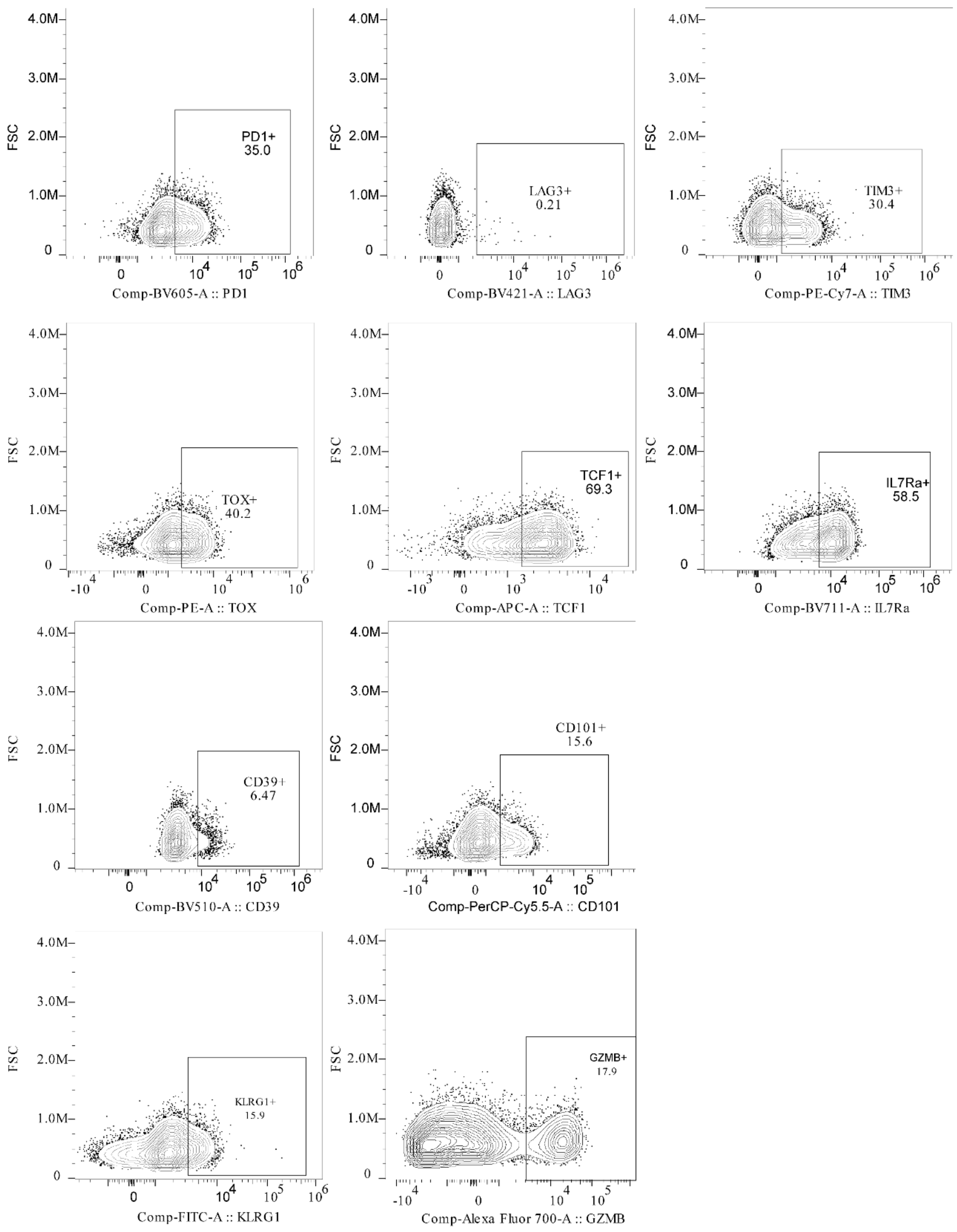

Supplementary Figure S16: Phenotypic profiling of individual markers in CD4$^+$ T cells. Flow cytometry gating strategy, showing individual markers for the control sample HD2.
