# Supplementary material for: Circulating Tumor Cells Predict Response to the DLL3-Targeting Bispecific Antibody Tarlatamab
Source: Cancer Discov. 2026 Jan 14;16(5):911–30. doi: 10.1158/2159-8290.CD-25-1483 (PMC13067943; doi:10.1158/2159-8290.CD-25-1483)
Supplement: Supplementary Figure S17 — shows graphs of CD4+ T cells phenotypic profiles for memory, activation and exhaustion. [file cd-25-1483_supplementary_figure_s17_suppsf17.pdf]

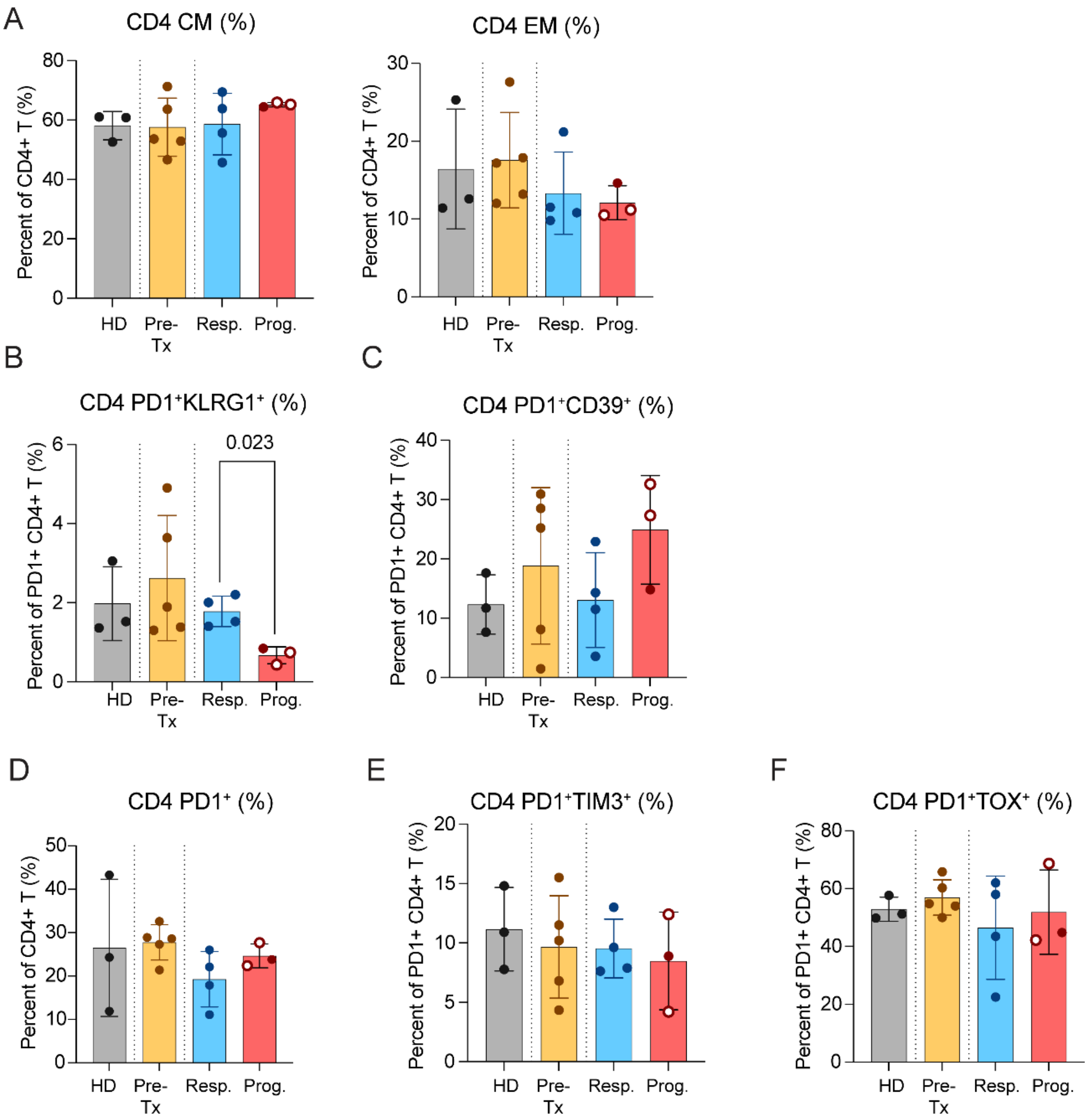

Supplementary Figure S17: Phenotypic profiling of memory, activation, and exhaustion markers in CD4⁺ T cells. Blood specimens analyzed from healthy donors (HD: gray, N=3), tarlatamab-untreated advanced SCLC (Pre-Tx: orange, N=5), SCLC cases at time of tarlatamab response (Resp: blue, N=4), and SCLC patients with acquired resistance to tarlatamab despite persistent DLL3 expression in CTCs (Prog: red, N=2, open circles denote two distinct measurements from one case). Multiple measurements from a single patient (where available) were averaged for statistical comparison using Student's unpaired t-test between Resp vs. Prog groups. **(A)** Plot showing percentage of central memory (CD45RA-CCR7+CD28+), and effector memory ($CD45RA^{-}CCR7^{-}CD28^{+/-}$) T cells within $CD4^{+}$ T cells. **(B)** Plot showing percentage of $KLRG1^{+}$ cells within activated ($PD\text{-}1^{+}$) $CD4^{+}$ T cells. **(C)** Plot showing percentage of $CD39^{+}$ cells within activated ($PD\text{-}1^{+}$) $CD4^{+}$ T cells. **(D)** Plot showing percentage of $PD1^{+}$ cells within total $CD4^{+}$ T cells. **(E)** Plot showing percentage of $TIM\text{-}3^{+}$ cells within activated ($PD\text{-}1^{+}$) $CD4^{+}$ T cells. **(F)** Plot showing percentage of TOX^{+} cells within activated ($PD\text{-}1^{+}$) $CD4^{+}$ T cells.
