# Supplementary material for: Circulating Tumor Cells Predict Response to the DLL3-Targeting Bispecific Antibody Tarlatamab
Source: Cancer Discov. 2026 Jan 14;16(5):911–30. doi: 10.1158/2159-8290.CD-25-1483 (PMC13067943; doi:10.1158/2159-8290.CD-25-1483)
Supplement: Supplementary Figure S18 — shows bar graphs which represent the expression of SEZ6 and B7H3 across SCLC tumors in Cohort B. [file cd-25-1483_supplementary_figure_s18_suppsf18.pdf]

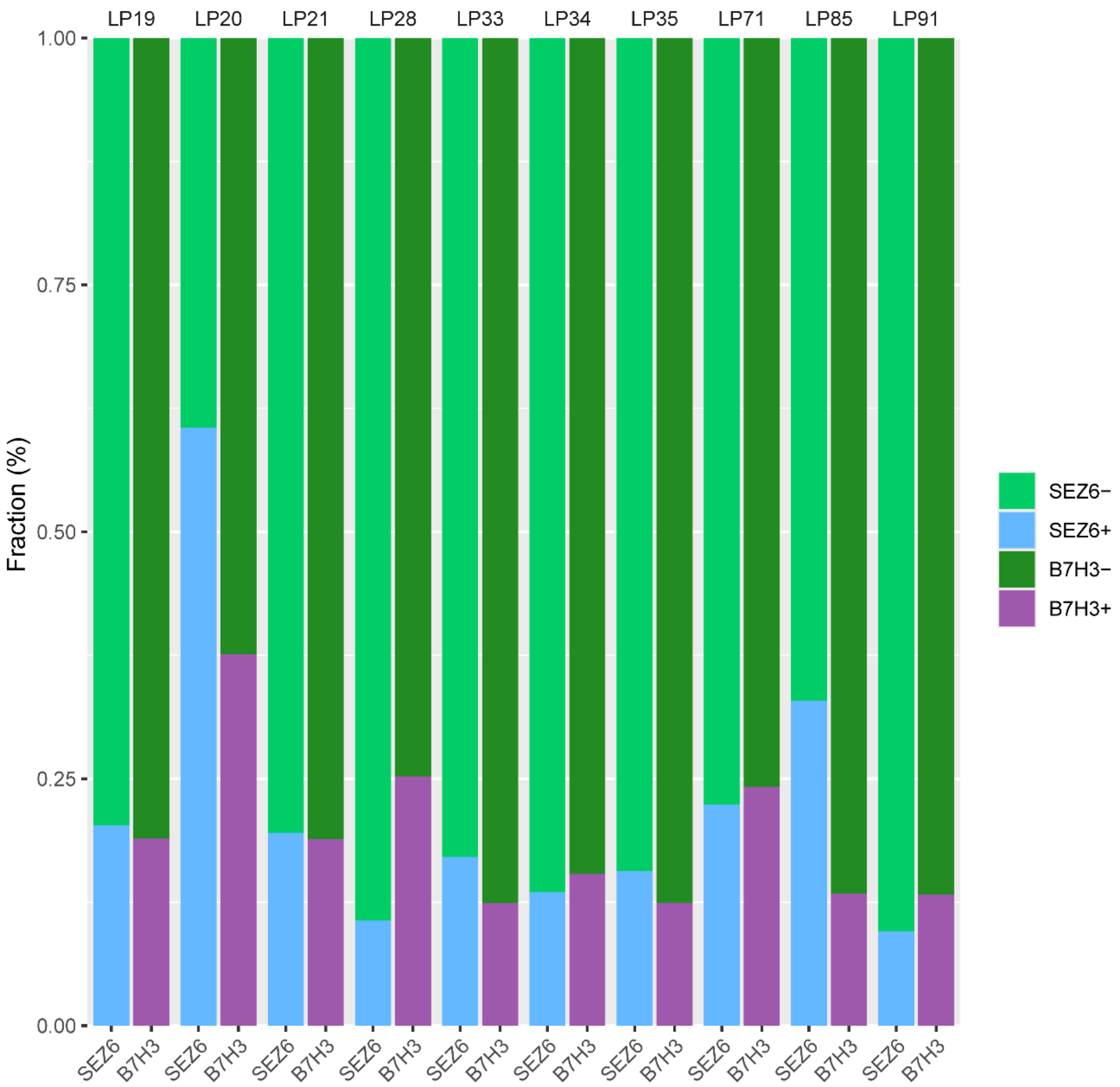

Supplementary Figure S18: Single-cell RNA expression of *SEZ6 and B7H3* across SCLC tumors. Bar graphs showing the fraction of single cells within a SCLC tumor (N=10, Cohort B) expressing SEZ6 and B7H3. Fractions indicate expression of SEZ6 negative (light green), SEZ6 positive (light blue), B7H3 negative (dark green), and B7H3 positive (purple).
