# Supplementary material for: Circulating Tumor Cells Predict Response to the DLL3-Targeting Bispecific Antibody Tarlatamab
Source: Cancer Discov. 2026 Jan 14;16(5):911–30. doi: 10.1158/2159-8290.CD-25-1483 (PMC13067943; doi:10.1158/2159-8290.CD-25-1483)
Supplement: Supplementary Figure S20 — shows shows bar graphs of expression of SEZ6 and B7H3 across SCLC tumors in Cohort C. [file cd-25-1483_supplementary_figure_s20_suppsf20.pdf]

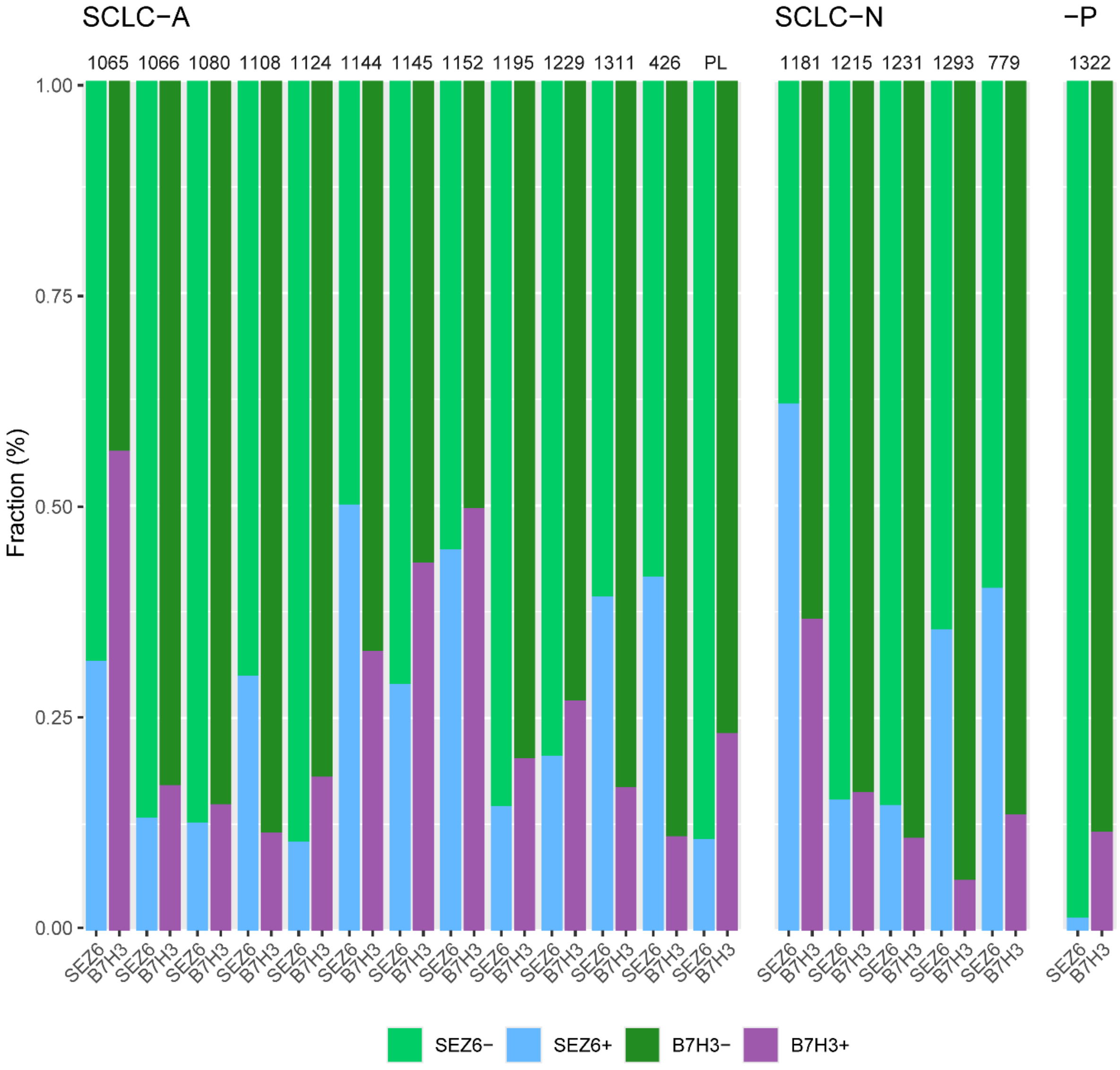


**Supplementary Figure S20: Single-cell RNA expression of *SEZ6 and B7H3* across SCLC tumors grouped by subtype in cohort C (previously published dataset) (1).** Bar graphs showing the fraction of single cells within a SCLC tumor (N=19, Cohort C) expressing SEZ6 and B7H3. Tumors are grouped by their predominant subtype: SCLC-A, SCLC-N, or SCLC-P. Fractions indicate expression of SEZ6 negative (light green), SEZ6 positive (light blue), B7H3 negative (dark green), and B7H3 positive (purple).
