# Supplementary material for: Circulating Tumor Cells Predict Response to the DLL3-Targeting Bispecific Antibody Tarlatamab
Source: Cancer Discov. 2026 Jan 14;16(5):911–30. doi: 10.1158/2159-8290.CD-25-1483 (PMC13067943; doi:10.1158/2159-8290.CD-25-1483)
Supplement: Supplementary Figure S21 — shows Venn diagrams of CNV-confirmed cancer cells and their coexpression of DLL3, SEZ6, B7H3 for tumors in Cohort C. [file cd-25-1483_supplementary_figure_s21_suppsf21.pdf]

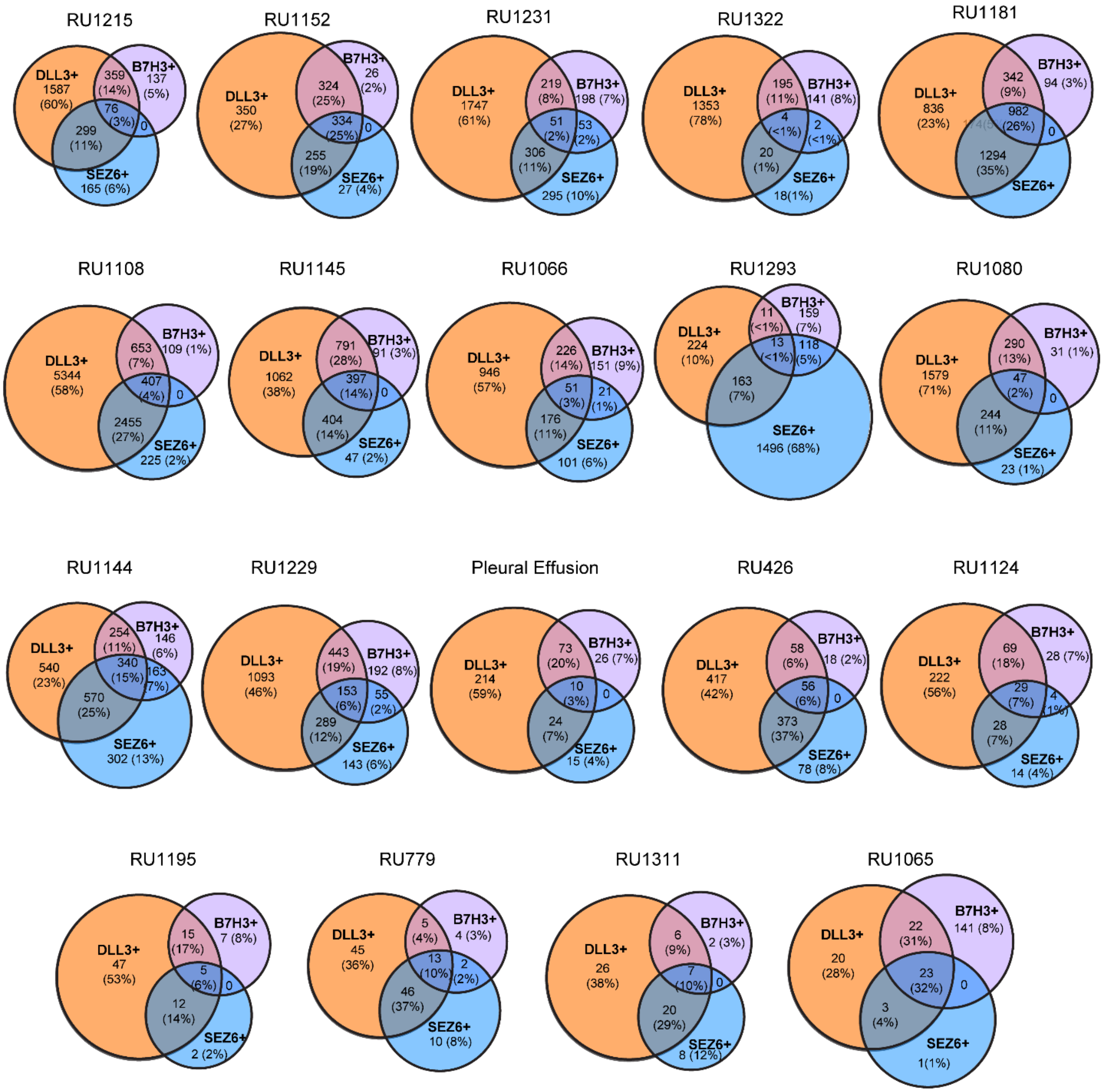


**Supplementary Figure S21: Coexpression of targetable SCLC epitopes *DLL3, SEZ6*, and *B7H3* in each tumor in Cohort C (previously published dataset) (1).** Venn diagram of Cohort C tumor cells with expression of any of the targetable epitopes showing the overlap in single cell expression for each of the three epitopes, *DLL3* (orange), *SEZ6* (blue), and *B7H3* (purple).
