# Supplementary material for: Circulating Tumor Cells Predict Response to the DLL3-Targeting Bispecific Antibody Tarlatamab
Source: Cancer Discov. 2026 Jan 14;16(5):911–30. doi: 10.1158/2159-8290.CD-25-1483 (PMC13067943; doi:10.1158/2159-8290.CD-25-1483)
Supplement: Supplementary Figure S22 — displays the coexpression of DLL3, SEZ6 and B7H3 for patient 36. [file cd-25-1483_supplementary_figure_s22_suppsf22.pdf]

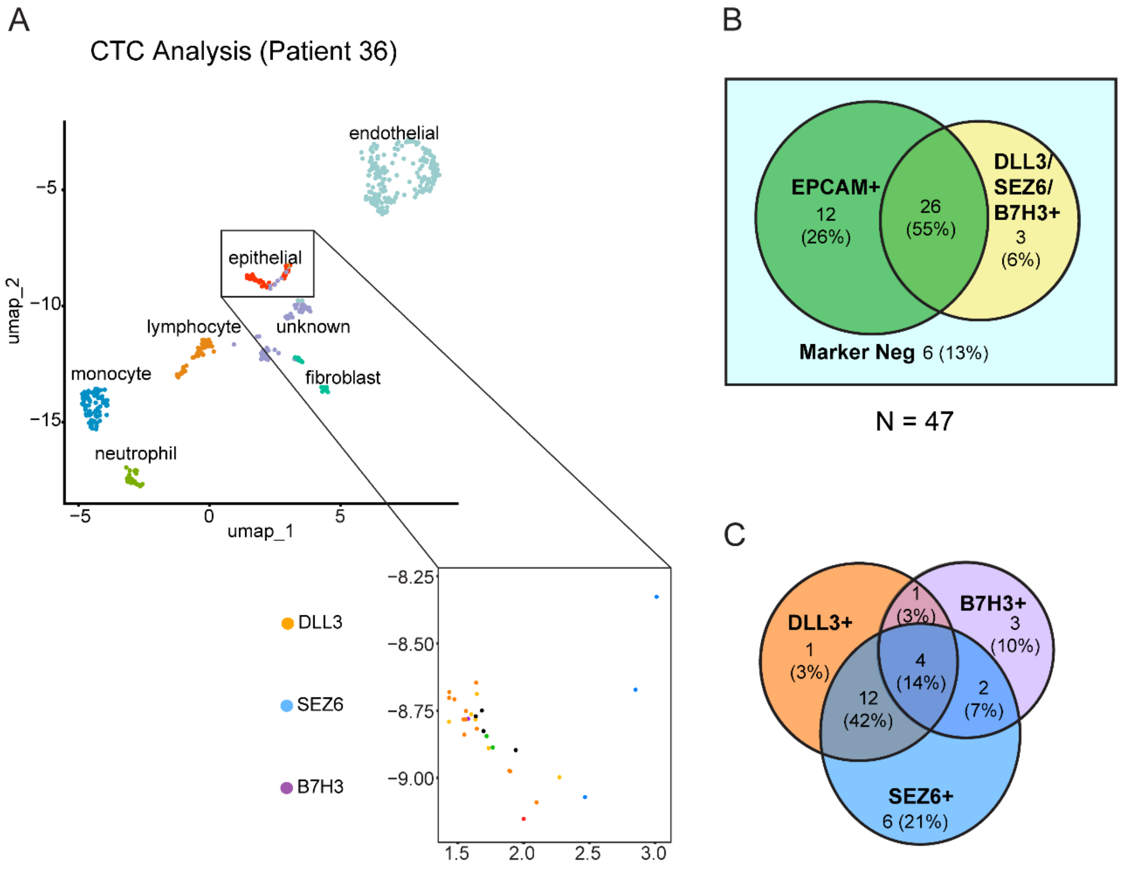

Supplementary Figure S22: Coexpression of targetable SCLC epitopes *DLL3, SEZ6*, and *B7H3* for patient-36. (A) UMAP of 10X single cell RNA-seq of CTC-enriched blood sample following microfluidic negative depletion of hematopoietic cells. CTCs (labeled as epithelial), identified by RNA-inferred CNV and expression of EpCAM, are shown in red, alongside neutrophils, monocytes, lymphocytes, fibroblasts, endothelial cells, and an unknown population without any canonical markers. The insert shows magnification of the CTC cluster, with expression of the targetable epitopes *DLL3*, *SEZ6,* and B7H3. **(B)** Venn diagram of all CNV-confirmed CTCs from patient-36 (N=47 CTCs), showing the distribution of expression of epithelial markers (green) versus any of the three targetable markers DLL3, SEZ6, B7H3 (yellow), with CTCs expressing neither shown in light blue**. (C)** Venn diagram showing the overlap in single cell expression of *DLL3*, *SEZ6*, and *B7H3* among the 29 CTCs from patient-36 expressing at least one of these epitopes.
