# Supplementary material for: Circulating Tumor Cells Predict Response to the DLL3-Targeting Bispecific Antibody Tarlatamab
Source: Cancer Discov. 2026 Jan 14;16(5):911–30. doi: 10.1158/2159-8290.CD-25-1483 (PMC13067943; doi:10.1158/2159-8290.CD-25-1483)
Supplement: Supplementary Figure S23 — shows Venn diagrams of the coexpression of DLL3, EpCAM, and cytokeratin in Cohort B, Cohort C, and patient 37. [file cd-25-1483_supplementary_figure_s23_suppsf23.pdf]

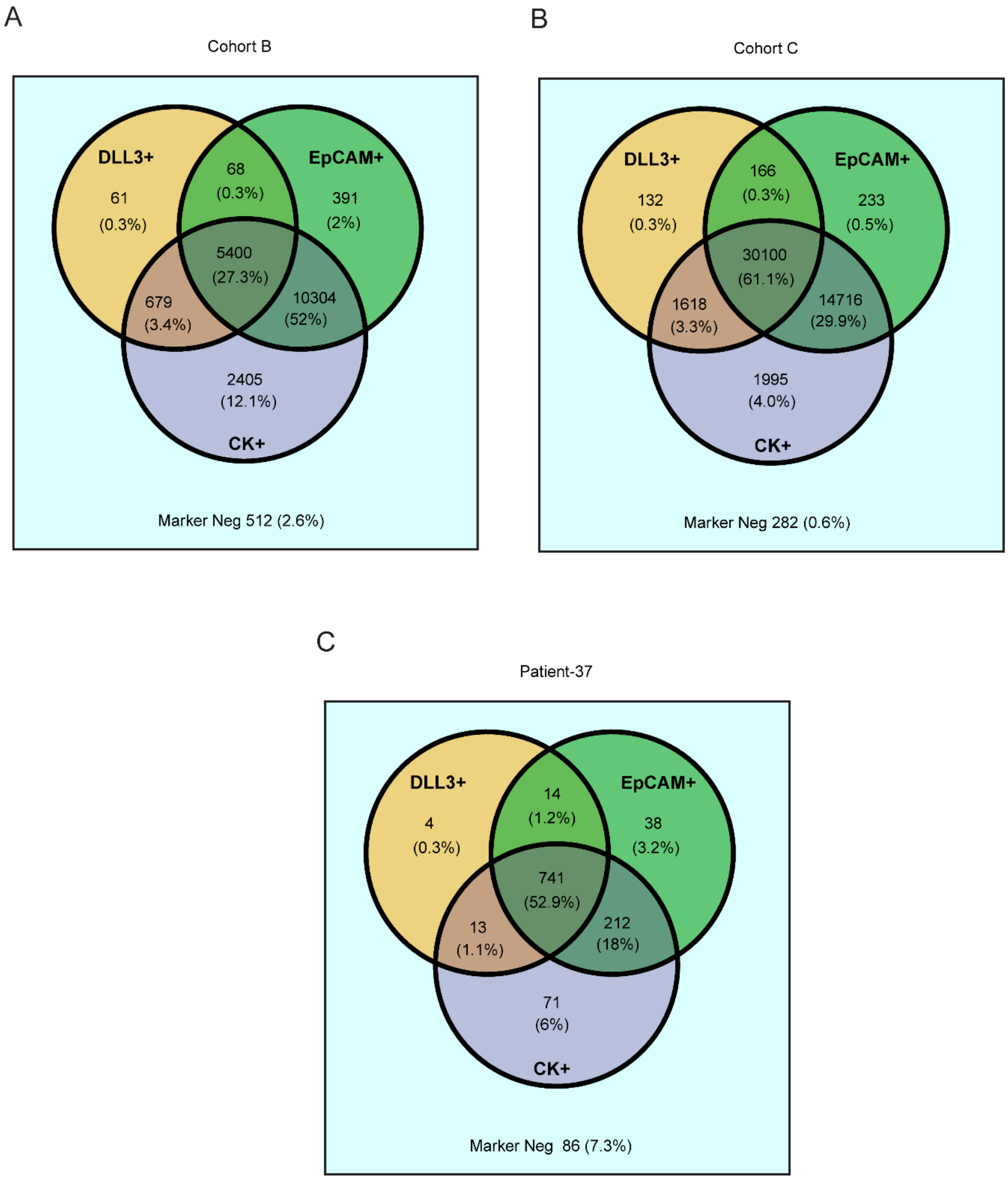

Supplementary Figure S23: Coexpression of DLL3, EpCAM, and cytokeratin (CK 4, 5, 6, 8, 10, 13, and 18) in (A) cohort B, (B) cohort C, and (C) patient-37. Venn diagram of all CNV-confirmed CTCs from patient-37 (N=1,179 CTCs), showing the distribution of expression of EpCAM (green), DLL3 (orange), and any cytokeratin (CK 4, 5, 6, 8, 10, 13, and 18, yellow). CTCs expressing neither of these markers are shown in light blue**.**
