# Supplementary material for: Circulating Tumor Cells Predict Response to the DLL3-Targeting Bispecific Antibody Tarlatamab
Source: Cancer Discov. 2026 Jan 14;16(5):911–30. doi: 10.1158/2159-8290.CD-25-1483 (PMC13067943; doi:10.1158/2159-8290.CD-25-1483)
Supplement: Supplementary Figure S24 — shows the distribution of DLL3 positive cells based on cell type for Cohort B and patient 37. [file cd-25-1483_supplementary_figure_s24_suppsf24.pdf]

A

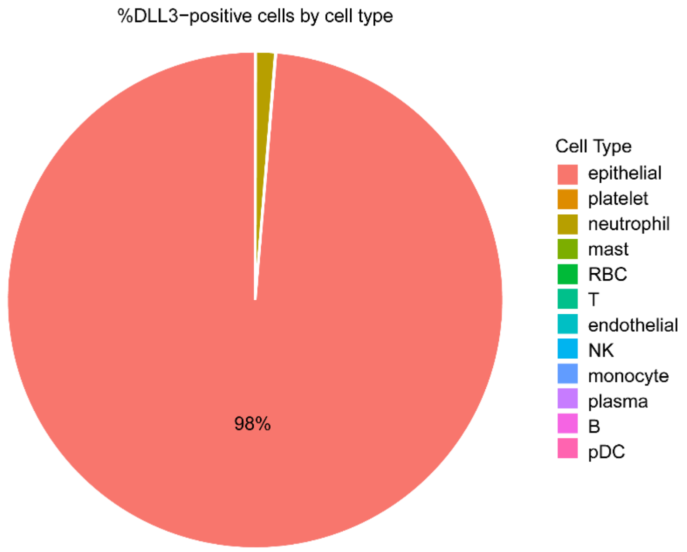

B

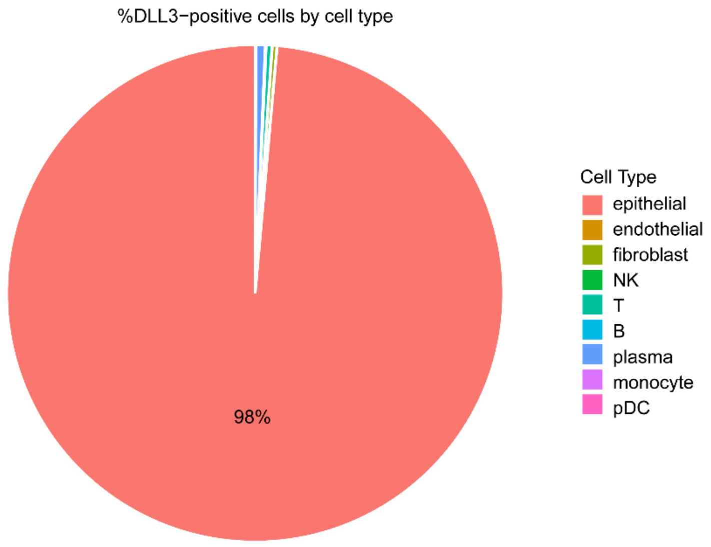

Supplementary Figure S24: DLL3-positive cell distribution across cell types in cohort B (A) and tumors from patient-37 (B). Virtually all DLL3+-positive cells are positive for aneuploidy (inferCNV) and express epithelial transcripts. Other cell identities (color code) were derived from RNA-seq analysis (inferCNV negative).
