# Supplementary material for: Circulating Tumor Cells Predict Response to the DLL3-Targeting Bispecific Antibody Tarlatamab
Source: Cancer Discov. 2026 Jan 14;16(5):911–30. doi: 10.1158/2159-8290.CD-25-1483 (PMC13067943; doi:10.1158/2159-8290.CD-25-1483)
Supplement: Supplementary References 1 — Supplementary References includes cited works for the supplementary figures. [file cd-25-1483_supplementary_references_1_suppsr1.pdf]

References:

1. Zand M, Ruan J. Network-Based Single-Cell RNA-Seq Data Imputation Enhances Cell Type Identification. Genes (Basel) **2020**;11(4) doi 10.3390/genes11040377.
2. Cheng Y, Ma X, Yuan L, Sun Z, Wang P. Evaluating imputation methods for single-cell RNA-seq data. BMC Bioinformatics **2023**;24(1):302 doi 10.1186/s12859-023-05417-7.
3. Chan JM, Quintanal-Villalonga Á, Gao VR, Xie Y, Allaj V, Chaudhary O*, et al.* Signatures of plasticity, metastasis, and immunosuppression in an atlas of human small cell lung cancer. Cancer Cell **2021**;39(11):1479-96.e18 doi 10.1016/j.ccell.2021.09.008.
